# Global Correlates of Cardiovascular Risk: A Comparison of 158 Countries

**DOI:** 10.3390/nu10040411

**Published:** 2018-03-26

**Authors:** Pavel Grasgruber, Jan Cacek, Eduard Hrazdíra, Sylva Hřebíčková, Martin Sebera

**Affiliations:** Faculty of Sports Studies, Masaryk University, Kamenice 5, 625 00 Brno, Czech Republic; jan.cacek@gmail.com (J.C.); hrazdira@fsps.muni.cz (E.H.); s.hrebickova@mail.muni.cz (S.H.); sebera@fsps.muni.cz (M.S.)

**Keywords:** cardiovascular diseases, risk factors, nutrition, ecological study

## Abstract

The aim of this study was a large-scale ecological analysis of nutritional and other environmental factors potentially associated with the incidence of cardiovascular diseases (CVDs) in the global context. Indicators of CVDs from 158 countries were compared with the statistics of mean intake (supply) of 60 food items between 1993 and 2011, obesity rates, health expenditure and life expectancy. This comparison shows that the relationship between CVD indicators (raised blood pressure, CVD mortality, raised blood glucose) and independent variables in the global context is influenced by various factors, such as short life expectancy, religiously conditioned dietary customs, the imprecision of some statistics and undernutrition. However, regardless of the statistical method used, the results always show very similar trends and identify high carbohydrate consumption (mainly in the form of cereals and wheat, in particular) as the dietary factor most consistently associated with the risk of CVDs. These findings are in line with the changing view of the causes of CVDs. Because only the statistics of raised blood glucose include people using medications and reflect true prevalence that is independent of healthcare, more objective data on the prevalence of CVDs are needed to confirm these observed trends.

## 1. Introduction

The view of the causes and prevention of cardiovascular diseases (CVDs) has been undergoing fundamental changes during recent years. Meta-analyses of observational studies and available clinical trials have not been able to find any reliable link between saturated fat and CVDs [1,2,3,4,5,6,7] which has been the pillar of official nutritional guidelines for more than three decades. A decrease of CVD prevalence/mortality occurs only when saturated fat is replaced by polyunsaturated fatty acids (PUFAs) which is more likely to be related to the beneficial effects of PUFAs on blood lipid profiles, rather than to any harmful role of saturated fat. Furthermore, even this conclusion was recently questioned and attributed to methodological flaws [8]. On the other hand, we are witnessing growing evidence of the fundamental roles of carbohydrates and high glycaemic index/load in the aetiology of CVDs [1,2,9,10,11,12].

Nutritional guidelines perpetuating the connection between saturated fat and CVDs were first introduced in the USA in 1977 and then in the United Kingdom in 1983 [13]. Although these recommendations were officially based on various sources, a key inspirational role can be attributed to the ‘Seven Countries Study’—an ecological research project that started in 1958 in seven countries of the world, from the USA to Japan. As we explained in our previous paper [14], the authors of this research concentrated on the statistics of coronary heart disease (CHD) mortality, without taking into account the poor quality of diagnosis in some areas of the world. Although their data suggested a strongly *positive* ecological correlation between CHD mortality and raised blood pressure, serum cholesterol and saturated fat intake [15], their following study, published in 1990, found a strongly *negative* relationship between stroke mortality and blood pressure/serum cholesterol [16]. Because the evidence from the contemporary clinical trials (interventional studies) was conflicting and insufficient [13], nutritional recommendations regarding saturated fat intake should have never been introduced.

During the last decade, more accurate and detailed statistical data on disease prevalence became easily accessible from internet databases. Concurrently, an internet version of the Food and Agriculture Organization Corporate Statistical Database (FAOSTAT) [17] enabled a mutual comparison of international statistics of food supply from which mean food consumption rates can be inferred. The FAOSTAT statistics of food supply are defined as ‘the total quantity of foodstuffs produced in a country added to the total quantity imported and adjusted to any change in stocks that may have occurred since the beginning of the reference period’. Thus, they express food availability (food disappearance) in a country within a given year and inevitably overestimate true food consumption, because a certain proportion of food is wasted, consumed by foreigners, animals, etc. However, in our own research, we observed that the FAOSTAT statistics of annual per capita food supply produced very impressive and meaningful findings, especially in relation to the basic components of diet (fat, protein, carbohydrates). For example, the correlation between male height in 93 countries and four animal proteins of the highest quality reached *r* = 0.84 (*p* < 0.001) [18].

With regard to such results, it is surprising that these statistics are still insufficiently utilized in nutrition science, because the currently used methodologies have very serious limitations. Most of the available knowledge comes from long-term observational studies that follow large samples of people for several decades, but their data are based on the self-reported consumption of selected food items. These studies, therefore, often produce unreliable and conflicting results. Controlled interventional (clinical) studies, which prescribe a specific diet and can establish causality, are very demanding and time-limited. In contrast, ecological (country-level) studies use official statistics that are available for a long period of time, and their accuracy can be far superior to self-reported information.

In our recent study dealing with food consumption and the prevalence of cardiovascular diseases (CVDs) in 42 European countries [14], we further verified the usefulness of this methodology and found biologically relevant correlations reaching up to *r* = 0.92 (*p* < 0.001)—a value that has hardly any analogy in the epidemiological literature. Although even strong ecological findings of this sort cannot conclusively establish causality at the individual level, they can be used as a starting point of medical hypotheses, and their validity can be supported by studies using different methodologies. The combination of different types of studies may therefore strengthen each other’s results, which is of key importance in a complicated field such as dietology. In this concrete case, our results agreed with the changing view of the causes of CVDs and pointed to high-glycaemic carbohydrates as the major CVD trigger. Distilled alcoholic beverages and sunflower oil emerged as other possible risk factors. In contrast, total fat and animal fat consumption were the most frequent negative correlates of CVD indicators, and additional statistics further highlighted high-fat dairy products (cheese), fruits (mainly oranges and mandarins) and tree nuts.

Because the ‘Seven Countries Study’ showed that the misapplication of ecological data can be a serious issue that undermines the very basis of this approach, particular attention should be paid to their accuracy. Furthermore, a traditional flaw of ecological studies conducted in the past was the use of a limited number of geographically and culturally incompatible countries, with a similarly limited number of independent variables that can be influenced by hidden confounding factors. Therefore, a well-conducted ecological study should utilize the maximum number of potentially significant variables and, if possible, it should compare the consistency of results across different regions and time periods.

In the present study, we expand our research of ecological correlates of CVDs to the rest of the world, using health and food statistics from 158 countries. Although the accuracy of data from developing countries may be lower, we hoped that the results would be consistent across different regions, and if they confirmed our findings from Europe, the support for their causal relationship with CVDs would further increase.

## 2. Methods

### 2.1. Statistics of Food Consumption

Similar to our previous study, we collected data on the mean food consumption (supply) from the FAOSTAT database [18] for the period 1993–2011 (in grams/day per capita). Our aim was to include as many food items from the FAOSTAT database as possible, but to limit spurious correlations, the minimum mean consumption was set at 5 grams/day. Data on some food items were missing in certain countries, but when their consumption rate in neighbouring countries was zero or close to zero, or when the data were apparently missing because of cultural reasons (the prohibition on alcohol and pork in Muslim countries), it was assumed that the consumption in the respective country is zero.

Fourteen food items (bananas, barley, cassava, grapefruit, lemons and limes, millet, onions, palm oil, pineapples, rye, sorghum, soybean oil, sweet potatoes, yams) had sufficiently high mean consumption rates, but were missing from too many countries for their consumption to be determined reliably. Therefore, these food items were excluded from the analysis. The data on oranges and mandarins were missing only in Myanmar. Given the important role of this food item in our previous study, the mean intake in Myanmar was estimated and computed as a mean of five neighbouring countries.

Altogether, the study included 60 food items. Fourteen of them were basic indicators of fat and protein intake, or their combinations (animal fat, animal protein, animal fat and animal protein, total energy, etc.). Another six basic indicators of the energy proportion were computed by us: % energy from carbohydrates in cereals (% CC energy), % energy from carbohydrates in starchy roots (% SRC energy), the combination of % CC energy and % SRC energy, % energy from carbohydrates and alcohol (% CA energy), % energy from alcoholic beverages (% alcoholic beverages energy), % plant food energy (excluding alcoholic beverages). Because the FAOSTAT database lists only information on fat and protein intake, the proportion of energy from carbohydrates was derived from fat and protein energy, assuming 9.0 kcal per gram of fat and 4.1 kcal per gram of protein.

Sixteen items designated the major food groups (e.g., alcoholic beverages, cereals, fruits, etc.). The remaining 24 items consisted of individual foods. Although their mean daily intakes are mostly low and are more likely to be tied to various confounders, their inclusion is definitely worthwhile, because they can potentially support results of studies based on different methodologies. The most important of them are wheat (198 g/day), potatoes (100 g/day), rice and beer (both 79 g/day).

### 2.2. Health Statistics

Health statistics were collected from the website of the World Health Organization (WHO) [19]. In some cases, there were differences in definitions, when compared with the data from Nichols et al. (for 2008) [20,21] which we used in our previous paper dealing with 42 European countries [14].

The statistics of raised blood pressure in the WHO database correlate very strongly with the data from Nichols et al. [20] (*r* = 0.81 in men, *r* = 0.93 in women; *p* < 0.001), but the difference is large enough to indicate that different sources or different methodologies were used (Figures S1 and S2). Because these WHO statistics are defined as the “age-standartized prevalence of raised blood pressure (systolic ≥140 or diastolic ≥90 mmHg) in adults aged >18 years”, they include a younger population, and they do not include people using blood pressure-lowering medications, which is also emphasized in the paper from which the input data were drawn [22]. As a result, the prevalence in Europe by WHO is lower by ~16% in men and ~17% in women, on average. Interestingly, the data of Nichols et al. correlate more strongly with food consumption in Europe, e.g., with total fat and animal protein (Figures S3–S6).

The statistics of the prevalence of raised cholesterol listed by Nichols et al. [20], which we used in our previous study dealing with CVDs in Europe, were also taken from the WHO database, as evidenced by the perfect correlation of both sources in both sexes (*r* = 1.00).

The data on CVD mortality in 42 European countries from Nichols et al. [21] are highly concordant with the statistics of CVD mortality by WHO (for 2012) (*r* = 0.95 in men, *r* = 0.93 in women; *p* < 0.001). However, there is a striking outlier—Georgia—in both sexes, and Albania in women (Figures S7 and S8). Remarkably, Georgia was also an outlier in all comparisons, including CVD mortality, in our previous study [14]. This strongly suggests that Nichols et al. underestimated CVD mortality in these two countries. Indeed, if we substitute the data of Nichols et al. with the WHO statistics, the correlation of total fat and animal protein with CVD mortality increases from *r* = −0.73 to *r* = −0.81 in men, and from *r* = −0.81 to *r* = −0.87 in women.

The data on the prevalence of raised blood glucose in 42 European countries from Nichols et al. [20] (for 2008) do not agree particularly well with the statistics of raised blood glucose from WHO (for 2010) (*r* = 0.65 in men, *r* = 0.86 in women; *p* < 0.001) (Figures S9 and S10). The statistics of WHO are defined as “the percent of the defined population with fasting glucose ≥126 mg/dL (7.0 mmol/L) or a history of diagnosis with diabetes or use of insulin or oral hypoglycaemic drugs” in the age category of >18 years. The statistics from Nichols et al. are defined as “the estimate of raised fasting blood glucose (≥7.0 mmol/L) or patients on medication (%)” in the age category of >25 years. This suggests that the methodology of data collection is not very different, but due to the targeting of higher age groups, the prevalence reported by Nichols et al. might be somewhat higher (which is indeed the case). Still, the statistics of WHO show stronger and very impressive correlations with nutrition in Europe, especially with % CC energy and % SRC energy (*r* = 0.72 in men, *r* = 0.92 in women) or total fat (*r* = −0.72 in men, *r* = −0.88 in women; *p* < 0.001).

### 2.3. Socioeconomic Statistics

The data on health expenditure, GDP (gross domestic product) per capita and life expectancy were obtained from the World Bank [23]. Not all these data were available from all countries. Therefore, a reasonable compromise must have been made between the number of examined variables and the number of included countries. Eventually, eight variables were selected. This means that together with the FAOSTAT statistics, there were nine factors in the main analysis that were available from 158 countries (Table 1; for complete statistics, see Supplementary dataset, Sheets S1–S3). The GDP per capita was not used in the study because its inclusion would decrease this number to 155, and it is very strongly associated with health expenditure per capita (*r* = 0.85, *p* < 0.001) which is a more meaningful correlate (confounder) of health statistics. The statistics of physical activity and smoking prevalence were largely incomplete, but due to their importance, they were analyzed independently, in samples of 123 and 115 countries, respectively.

### 2.4. Statistical Analyses

Altogether, the main statistical analysis included 68 variables: 60 food items, six health indicators divided by sex (raised blood cholesterol, CVD mortality, raised blood pressure, raised blood glucose, obesity, mean BMI (Body mass index), health expenditure per capita and life expectancy divided by sex. Statistical analyses were performed using the software SPSS Statistics 24.0.(IBM, Inc., Armonk, NY, USA) Raised blood cholesterol, CVD mortality, raised blood pressure and raised blood glucose were selected as the main dependent variables in the study. The correlates of life expectancy, obesity and BMI will be examined in detail in a separate paper.

At first, we calculated simple (unadjusted) Pearson linear correlations with the total sample of 158 countries. To examine the consistency of findings across different parts of the world, Pearson linear correlations were also computed in Europe (42 countries), the world outside Europe (116 countries), North Africa/Asia/Oceania (47 countries), America (29 countries) and Sub-Saharan Africa (40 countries). Considering that the WHO database does not indicate the quality of data, a similar comparison was performed with countries divided according to health expenditure per capita: above 500 USD, above 1000 USD, between 500–2000 USD and above 2000 USD. In the case of raised blood pressure and CVD mortality, we also computed partial correlations adjusted for the most likely confounding factors (health expenditure, smoking) in a multiple regression. Eventually, Pearson linear correlations were performed even with the health indicators, which were not incorporated in the main analysis because of missing data from multiple countries (physical activity, smoking prevalence).

Subsequently, a factor analysis with all 68 variables (or 75 variables, respectively, when the division by sex is taken into account) was performed. The factor analysis groups variables according to certain similar characteristics (factors) and graphically visualizes their mutual relationships in two- or three-dimensional plots. This solves a whole range of problems associated with multicollinearity—the key statistical problem in the present study.

Other tools that we used for the reduction of multicollinearity were the ridge regression, LASSO (least absolute shrinkage and selection operator) regression and elastic net regression. These regression methods are aimed at identifying the best predictors out of a set of variables that are mutually highly correlated. They work with all independent variables at once and are based on the penalization (artificial lowering) of *beta* regression coefficients. The changing size of the penalization creates different models with different prediction errors, and a model with the lowest prediction error (ideally using low penalization) is selected as optimal. In the results of the ridge regression, all variables are ranked according to the size of their beta coefficients. The LASSO regression is more selective and with increasing penalization, it shrinks *beta* coefficients in the majority of variables to zero. The elastic net regression is basically a combination of these two methods [24]. In all cases, optimal models with the lowest prediction errors were used, computed via the bootstrapping method. Bootstrapping works with random combinations of independent variables with replacement, creates many additional models for each penalization level, and then also computes their mean result. This helps to eliminate various anomalies (for a more detailed explanation, see SPSS Statistics version 24.0, ibm.com).

Finally, we performed an analogy of fixed-effects models and examined temporal changes in the relationship between the actual CVD incidence and food consumption in single years. This procedure can potentially identify a time period that was critical for the development of CVDs. In addition, it can also reveal long-term collinearity between some food items which would help in identifying confounding factors. The inter-item collinearity was examined via the regression slope test that compares the slope of two regression trend lines. The higher the probability value (*p*-value) in this test, the more two trend lines run parallel to each other [25].

## 3. Results

### 3.1. Pearson Linear Correlations

Detailed results of the Pearson linear correlations are presented in Table 2, Table 3, Table 4 and Table 5 and in Tables S1–S8. Partial (adjusted) correlations of raised blood pressure and CVD mortality are displayed in Table 6.

#### 3.1.1. Raised Blood Pressure

**Figure 1:**
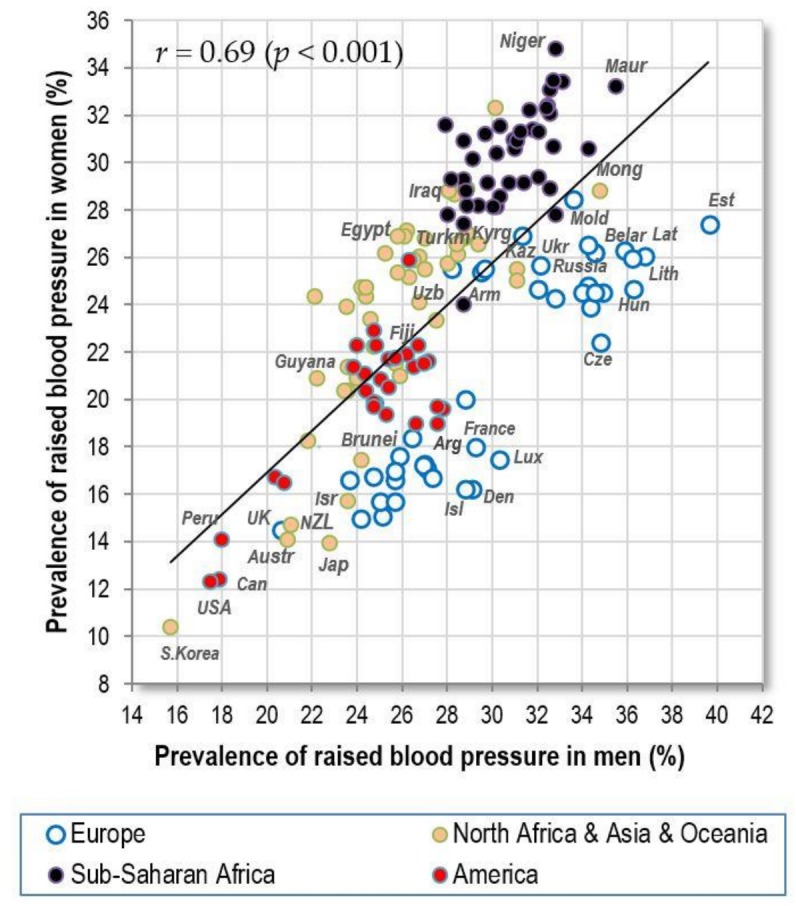
Relationship between the prevalence of men‘s and women’s raised blood pressure (%; WHO, 2010).

**Figure 2:**
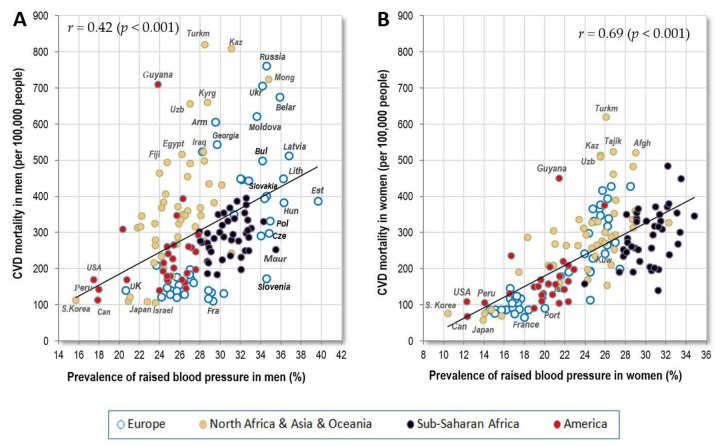
(**A**) Relationship between the prevalence of men’s raised blood pressure (%; WHO, 2010) and CVD mortality (WHO, 2012); (**B**) Relationship between the prevalence of women’s raised blood pressure (%; WHO, 2010) and CVD mortality (WHO, 2012).

The prevalence of raised blood pressure is the highest in men from Eastern Europe and Sub-Saharan Africa, and in women from Sub-Saharan Africa. The men’s and women’s values quite strongly correlate (*r* = 0.69, *p* < 0.001), but the prevalence in men from less developed countries of Asia, Africa and America does not differ from that in women. This contrasts with the situation in Europe and in some other highly developed countries (USA, Japan, South Korea, Israel etc.) where men have a much higher prevalence of raised blood pressure than women (Figure 1). As a result, the correlation between raised blood pressure and CVD mortality is much weaker in men (*r* = 0.42, *p* < 0.001) than in women (*r* = 0.69, *p* < 0.001) (Figure 2A,B).

**Figure 3:**
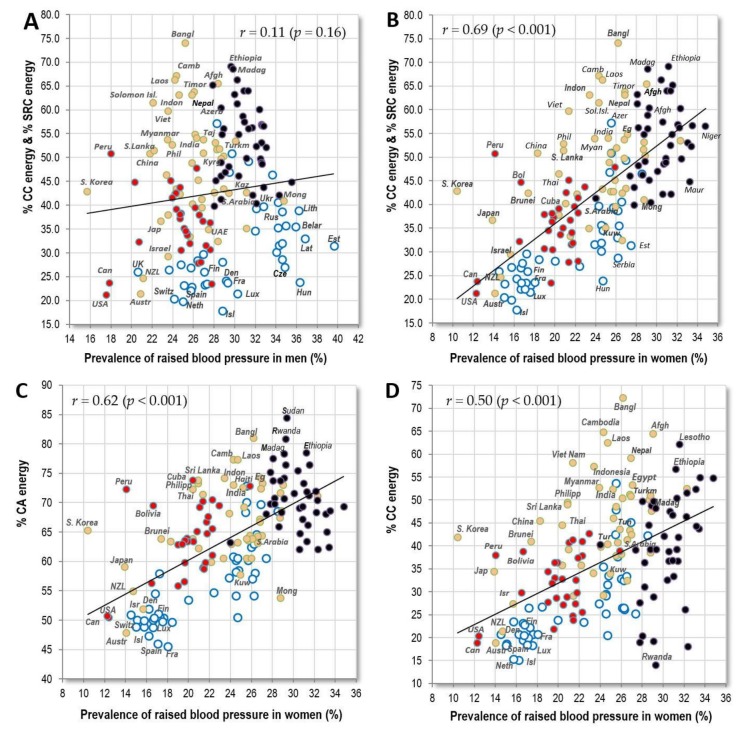

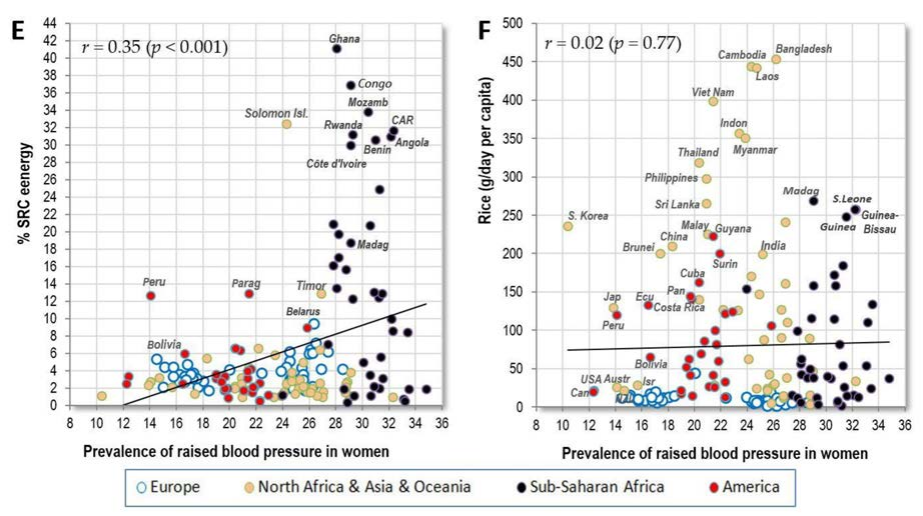
(**A**) Relationship between the prevalence of men’s raised blood pressure (%; WHO, 2010) and the mean proportion of carbohydrate energy from cereals and starchy roots (% CC energy and % SRC energy) in the diet (FAOSTAT, 1993–2011); (**B**) Relationship between the prevalence of women‘s raised blood pressure (%; WHO, 2010) and the mean proportion of carbohydrate energy from cereals and starchy roots (% CC energy and % SRC energy) in the diet (FAOSTAT, 1993–2011); (**C**) Relationship between the prevalence of women’s raised blood pressure (%; WHO, 2010) and the mean proportion of energy from carbohydrates and alcohol (% CA energy) in the diet (FAOSTAT, 1993–2011); (**D**) Relationship between the prevalence of women’s raised blood pressure (%; WHO, 2010) and the mean proportion of carbohydrate energy from cereals (% CC energy) in the diet (FAOSTAT, 1993–2011); (**E**) Relationship between the prevalence of women’s raised blood pressure (%; WHO, 2010) and the mean proportion of carbohydrate energy from starchy roots (% SRC energy) in the diet (FAOSTAT, 1993–2011); (**F**) Relationship between the prevalence of women’s raised blood pressure (%; WHO, 2010) and the mean consumption of rice (g/day per capita; FAOSTAT, 1993–2011).

The explanation for this discrepancy may lie in the substantially shorter life expectancy of men relative to women, particularly in the former USSR republics (Figure S11). In other words, many men in certain countries do not reach the critical age when this CVD indicator starts to manifest itself. In any case, men’s statistics of raised blood pressure do not correlate with CVD mortality in America and Sub-Saharan Africa (Table 2). Their relationships with food consumption are also quite weak (compare Figure 3A,B), and in Sub-Saharan Africa, they even tend to go in the opposite direction than in women. Therefore, whatever the reason, men’s statistics appear to be less credible. However, if we are to identify any meaningful factor that influences men’s global statistics, it is apparently alcohol, particularly in its distilled form (Figures S12 and S13). In fact, the drinking distilled alcohol is probably indirectly reflected even by the natural substrates of its home production in Eastern Europe (starchy roots, % SRC energy, potatoes) which are themselves sources of high-glycaemic carbohydrates.

Table 3 shows that women’s raised blood pressure has the strongest positive correlation with the proportion of carbohydrate energy coming from cereals and starchy roots (*r* = 0.69, *p* < 0.001) (Figure 3B), followed by % plant food energy (*r* = 0.64, *p* < 0.001) and % energy from carbohydrates and alcohol (*r* = 0.62, *p* < 0.001) (Figure 3C). Even after adjusting for health expenditure, these food items remain some of the few that have a significantly positive relationship with women’s raised blood pressure (Table 6). Cereals make up the largest proportion in the diet of the tropical belt of Asia and in Sub-Saharan Africa (Figure 3D). Starchy roots are consumed mainly in Sub-Saharan Africa (Figure 3E). These findings thus go in the same direction as those from our previous study in which we observed a link between CVD and high carbohydrate consumption [14].

Interestingly, the nations of South and East Asia have a disproportionately lower prevalence of raised blood pressure, despite a high proportion of cereals in their diet and irrespective of their economic prosperity. For example, Japan and South Korea have a similar prevalence to ‘Western’ nations with a two-fold lower proportion of energy from cereal carbohydrates. Figure 3F shows that all these Asian nations consume cereals in the form of rice, whereas others consume them mostly as wheat and maize (Figures S14 and S15).

**Figure 4:**
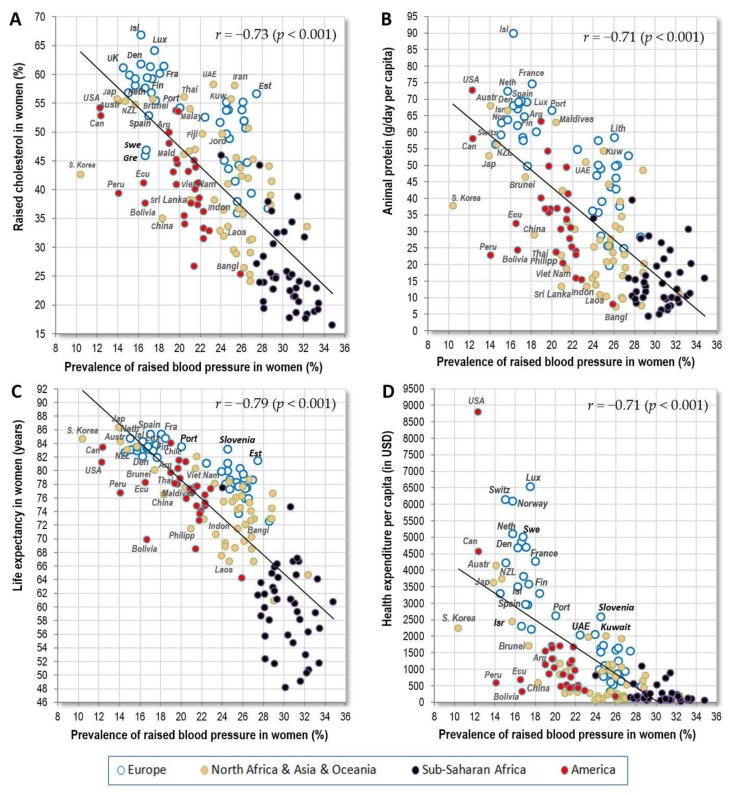
(**A**) Relationship between the prevalence of women’s raised blood pressure (%; WHO, 2010) and the prevalence of women’s raised cholesterol (%) (WHO, 2008); (**B**) Relationship between the prevalence of women’s raised blood pressure (%; WHO, 2010) and the mean consumption of animal protein (g/day per capita; FAOSTAT, 1993–2011); (**C**) Relationship between the prevalence of women’s raised blood pressure (%; WHO, 2010) and women‘s life expectancy (World Bank, 2012). (**D**) Relationship between the prevalence of women’s raised blood pressure (%; WHO, 2010) and health expenditure per capita for 2012 (in USD).

The strongest negative correlates of women’s raised blood pressure are displayed in Figure 4A–D: raised cholesterol (*r* = −0.73) and animal protein (*r* = −0.71) among nutrition-related variables, and life expectancy (*r* = −0.79) with health expenditure (*r* = −0.71, *p* < 0.001) among socioeconomic statistics. All these results are again similar to those from Europe, but the global connection between raised blood pressure and raised cholesterol is now even slightly stronger, and the main negative dietary correlate in Europe was total fat and animal protein. At the same time, the correlation of raised blood pressure with health expenditure has a strikingly different shape than those for the other three variables. In addition, each of these three variables retains high significance after adjusting for health expenditure (*p* < 0.001) (Table 6). This minimizes the possibility that health expenses could work as a significant confounding factor.

The strongly negative relationship between raised blood pressure and life expectancy indirectly indicates that women’s statistics of raised blood pressure reflect very well the prevalence of CVDs which are the major cause of death worldwide [26]. In contrast, we do not find such a strong relationship in men (*r* = −0.39, *p* < 0.001), and the graphic comparisons show quite convincingly that men’s statistics of raised blood pressure in developing countries are deeply underestimated, especially in Sub-Saharan Africa (Figures S16 and S17).

#### 3.1.2. Raised Cholesterol

**Figure 5:**
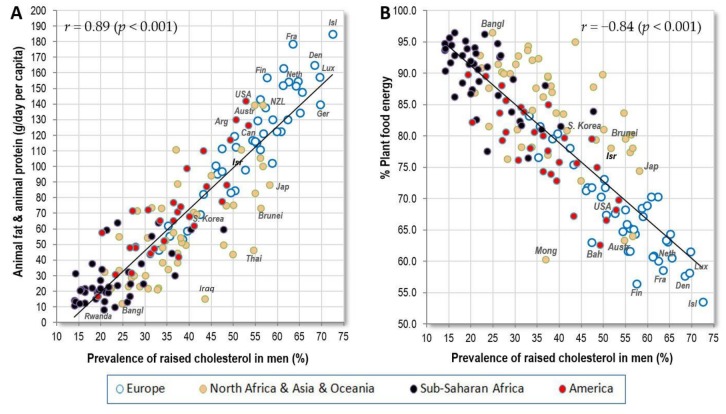
(**A**) Relationship between the prevalence of men’s raised cholesterol (%; WHO, 2010) and the mean consumption of animal fat and animal protein (g/day per capita; FAOSTAT, 1993–2011); (**B**) Relationship between the prevalence of men’s raised cholesterol (%; WHO, 2010) and the mean proportion of plant food energy in the diet (FAOSTAT, 1993–2011).

The strongest nutritional predictors of raised cholesterol in the present study are very similar to those observed in Europe [14]: animal fat and animal protein (*r* = 0.89 in men, *r* = 0.85 in women; *p* < 0.001) (Figure 5A) and % plant food energy (*r* = −0.84 in men, *r* = −0.80 in women; *p* < 0.001) (Figure 5B). These exceptional results have a very solid biological basis, because saturated fat from animal sources is the main trigger of total cholesterol, whereas carbohydrates (coming overwhelmingly from plant sources) decrease it the most [27]. Total cholesterol thus largely expresses the proportion of animal food in the diet. It also follows that the statistics of raised cholesterol can be regarded as very reliable, and they can be used as one of the key pillars of this study.

#### 3.1.3. Cardiovascular Mortality

**Figure 6:**
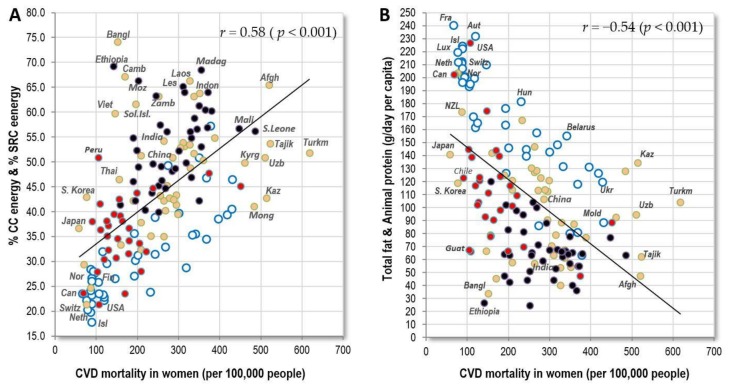

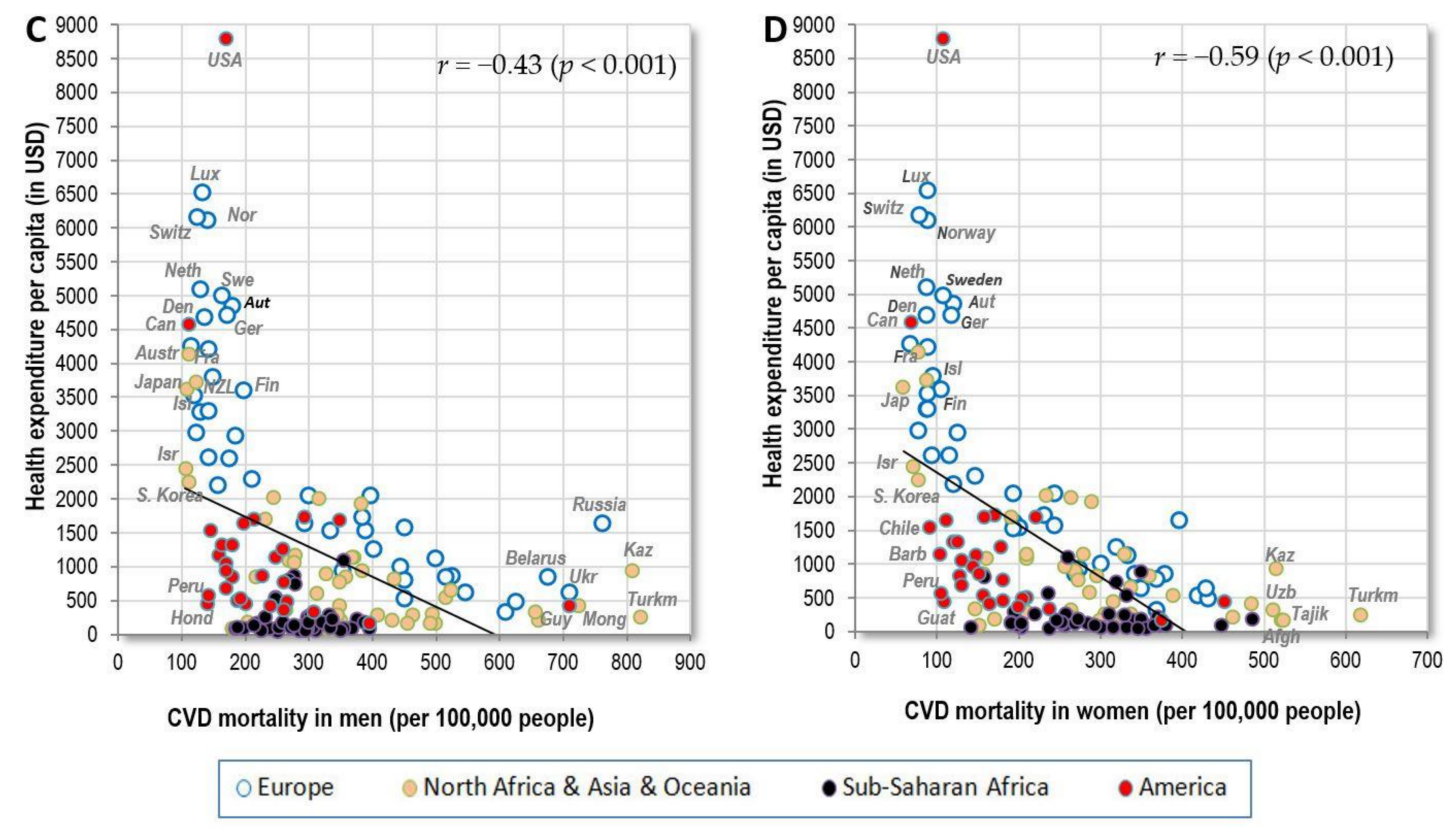
(**A**) Relationship between women’s CVD mortality (WHO, 2012) and the mean proportion of carbohydrate energy from cereals and starchy roots (% CC energy & % SRC energy) in the diet (FAOSTAT, 1993–2011); (**B**) Relationship between women’s CVD mortality (WHO, 2012) and the mean consumption of total fat and animal protein (g/day per capita; FAOSTAT, 1993–2011); (**C**) Relationship between men’s CVD mortality (WHO, 2012) and health expenditure per capita for 2012 (in USD; (**D**) Relationship between women’s CVD mortality (WHO, 2012) and health expenditure per capita for 2012 (in USD).

The highest rates of CVD mortality in men occur in the former USSR republics and Central Asia, but even in Egypt, Iraq and Guyana. In women, Central Asia is by far the most affected region. In general, the relationships between CVD mortality and exogenous factors are substantially weaker than in the case of raised blood pressure, although the trends are similar. The strongest positive correlates are cereals and raised blood pressure in men (both *r* = 0.43), and raised blood pressure in women (*r* = 0.69, *p* < 0.001). Out of all dietary factors, % CC energy and % SRC energy in women reach the highest *r*–values (*r* = 0.58, *p* < 0.001) (Figure 6A). Another important factor that could explain the strikingly high CVD mortality in Central Asia is high salt intake, but these statistics are still based only on rough estimates from a limited number of countries [28].

Total fat and animal protein, which have a very impressive negative relationship to CVD mortality in Europe, even higher than health expenditure (Table 2 and Table 3), show only moderate *r*-values in the total sample (*r* = −0.30 in men, *r* = −0.54 in women; *p* < 0.001) because CVD mortality in non-European countries is lower than expected from the European trend line (Figure 6B). As a result, the list of global negative correlates in both sexes is dominated by the indicator of healthcare–health expenditure (*r* = −0.43 in men, *r* = −0.59 in women; *p* < 0.001) (Figure 6C,D). Although the adjustment for health expenditure completely erases the significance of total fat and animal protein in women (*p* = 0.21), that of % CC energy and % SRC energy is retained (*p* < 0.001), and wheat reaches the highest partial correlation, followed by cereals (Table 6). Similar to Europe, health expenses above 2000 USD per capita do not bring any additional benefits, but reduced CVD mortality in highly developed countries (e.g., Luxembourg, Ireland) obviously blurs relationships with certain food items, such as alcohol, that are otherwise associated weakly positively with raised blood pressure (Figures S18 and S19).

Figures S20–S23 display correlations of CVD mortality with raised cholesterol (*r* = −0.24 in men, *p* = 0.003; *r* = −0.52 in women, *p* < 0.001) and life expectancy (*r* = −0.33 in men, *r* = −0.54 in women; *p* < 0.001) which are also lower than in raised blood pressure. At the same time, we can clearly observe disproportionately lower rates of CVD mortality outside Europe, especially in Sub–Saharan Africa. Understandably, the statistics of CVD mortality in developing countries are distorted because of the much higher premature mortality from other causes (infectious diseases, malnutrition, violence, etc.), but this fact does not explain why life expectancy correlates more strongly with CVD prevalence (raised blood pressure). Therefore, these results are somewhat puzzling and raise doubts about the quality of CVD mortality statistics. Indeed, with growing health expenditure (and hence increasing data accuracy), this relationship starts to reverse. Among the 31 countries with health expenditure above 2000 USD per capita, the correlation between CVD mortality and life expectancy increases up to *r* = −0.84 in men and *r* = −0.82 in women (*p* < 0.001), whereas in the case of raised blood pressure vs. life expectancy, it is much lower (*r* = −0.43, *p* = 0.015 in men; *r* = −0.54, *p* = 0.002 in women). It is also noteworthy that CVD mortality in Sub-Saharan Africa does not correlate with a single variable in men and does so with only three variables in women. In contrast, the data on raised blood pressure often come from internationally sponsored nationwide surveys (e.g., WHO-sponsored STEPS surveys [22]), and despite their persisting imperfection, they should be more trustworthy.

#### 3.1.4. Raised Blood Glucose

**Figure 7:**
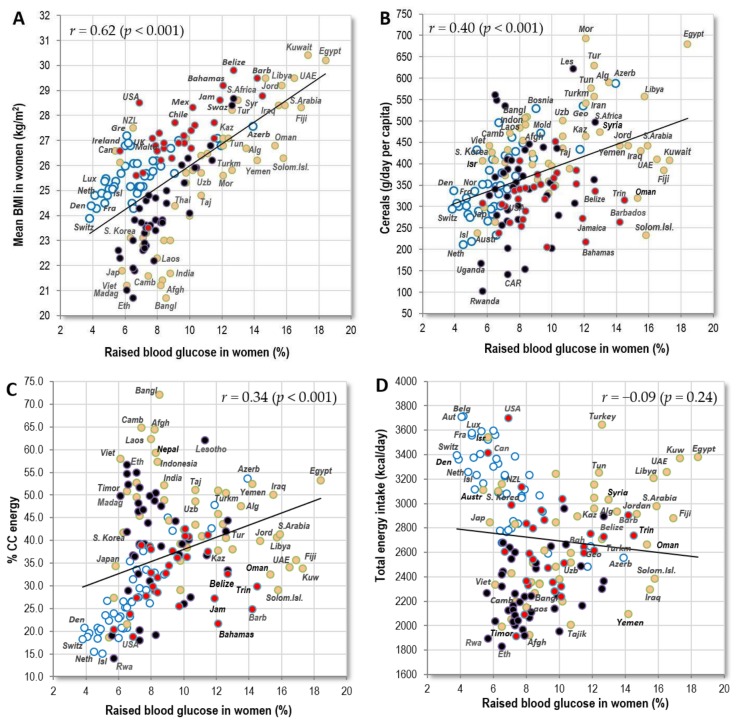

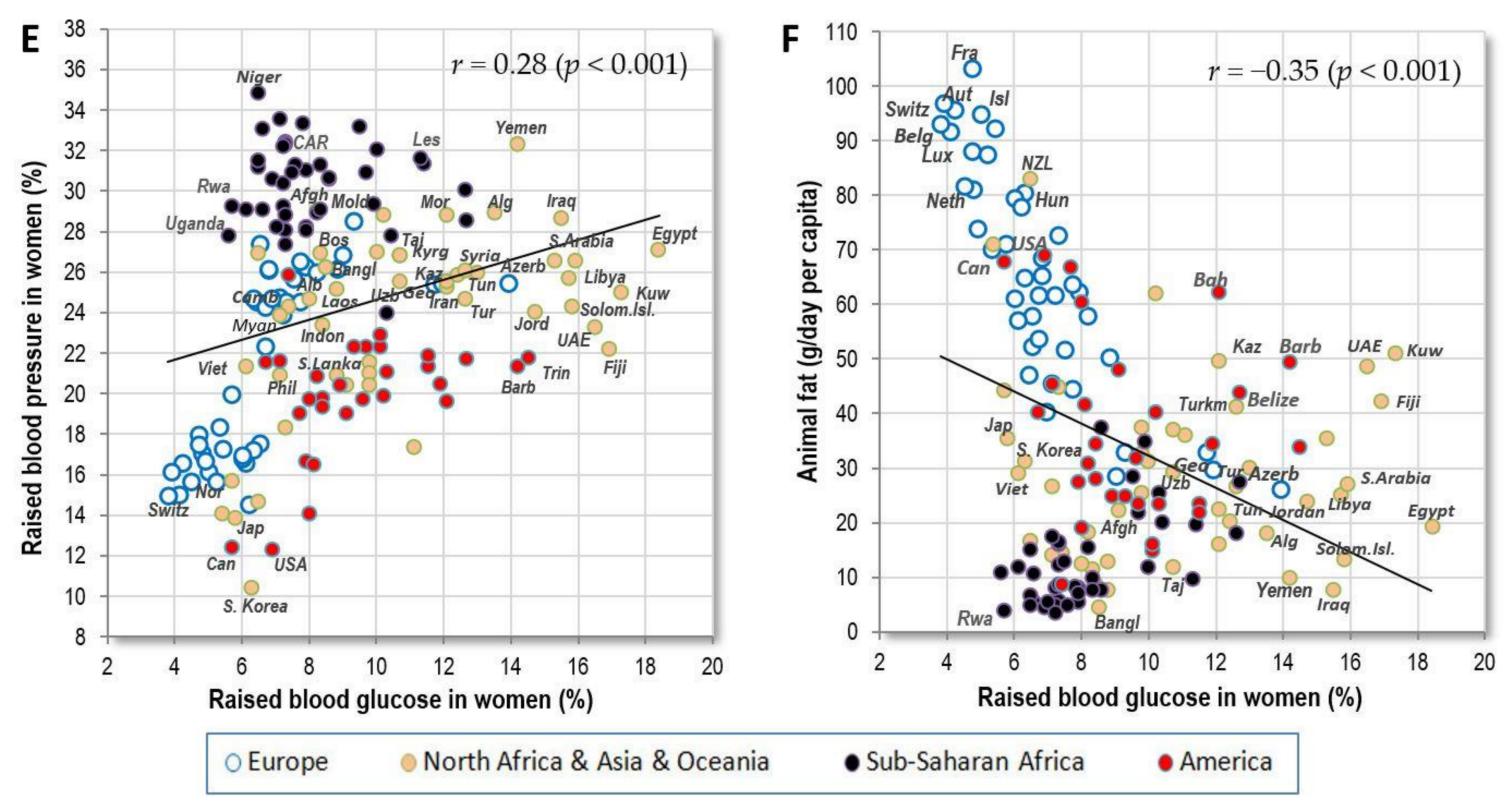
(**A**) Relationship between women’s raised blood glucose (%; WHO, 2010) and the mean BMI in women (WHO, 2010); (**B**) Relationship between women’s raised blood glucose (%; WHO, 2010) and the mean consumption of cereals (g/day per capita; FAOSTAT, 1993–2011); (**C**) Relationship between women’s raised blood glucose (%; WHO, 2010) and the mean proportion of carbohydrate energy from cereals (% CC energy) in the diet (FAOSTAT, 1993–2011); (**D**) Relationship between women’s raised blood glucose (%; WHO, 2010) and the mean total energy intake (kcal/day, FAOSTAT, 1993–2011); (**E**) Relationship between women’s raised blood glucose (%; WHO, 2010) and women’s raised blood pressure (%; WHO, 2010); (**F**) Relationship between women’s raised blood glucose (%; WHO, 2010) and the mean consumption of animal fat (g/day per capita; FAOSTAT, 1993–2011).

In the global comparison, raised blood glucose is the most common factor in Muslim, Pacific and Caribbean countries. It is most strongly associated with indicators of obesity, such as high BMI (*r* = 0.40 in men, *r* = 0.62 in women; *p* < 0.001) (Figure 7A) and with the consumption of cereals (*r* = 0.35 in men, *r* = 0.40 in women; *p* < 0.001) (Figure 7B), especially in the form of wheat (*r* = 0.43 in men, *r* = 0.31 in women; *p* < 0.001). Total energy intake from cereal carbohydrates (CC energy), which was not included among the independent variables in this study, produces very similar correlations (*r* = 0.36 in men, *r* = 0.44 in women; *p* < 0.001). These results are not surprising because the links between raised blood glucose, obesity, type 2 diabetes and CVDs are well established [29]. High-glycaemic foods, such as refined cereals, fit into this context as well.

The strongest negative correlates of raised blood glucose are alcoholic beverages and pork, which is a finding that makes little sense at first glance. However, it is easy to explain, when we realize that both these foods are prohibited in Muslim countries, who consume the highest amount of cereals in the world and suffer from very high rates of obesity. Inevitably, zero consumption of alcoholic beverages and pork is linked to the highest rates of raised blood glucose. Therefore, it is a purely spurious correlation (Figures S24 and S25).

Outside Europe, the proportion of energy from carbohydrate sources correlates either neutrally or weakly *negatively* with raised blood glucose. Consequently, the relationship between these factors and raised blood glucose is only slightly positive in the global comparison (Figure 7C). The explanation for this paradox lies in the fact that outside Europe, the proportion of carbohydrates in the diet increases with malnutrition and poverty. Some countries, such as Afghanistan, Bangladesh, Cambodia and Laos, consume >60% of total energy intake in the form of cereal carbohydrates, but they otherwise suffer from malnutrition and a very low caloric intake (Figure 7D). This means that their absolute intake of cereals is only moderate, and due to widespread undernutrition, the prevalence of obesity is very low. As a result, these countries have a rather low prevalence of blood glucose, but not necessarily blood pressure (Figure 7E). If we consider only wealthier, well-nourished countries with health expenditure above 500 USD, the correlation coefficients increase dramatically (compare Figures S26–S29). For example, the importance of animal fat disproportionately grows from *r* = −0.12 to −0.53 in men and from *r* = −0.35 to *r* = −0.70 in women (Figure 7F, Figures S28 and S29).

In summary, raised blood glucose manifests in relatively wealthy countries, where the *absolute* intake of energy from cereals is high and, in addition, the rates of obesity are high as well. Its low prevalence has no clear common denominator because it is associated with high fat intake (in Europe and highly developed countries in general) on one hand, and undernutrition/low obesity rates in developing countries on the other hand.

### 3.2. Pearson Linear Correlations in Items with Insufficient Data

This comparison primarily concerns smoking prevalence, which is available for 115 countries (Tables S9 and S10). Remarkably, in this limited sample (Table 7), current smoking of any tobacco product is the strongest positive correlate of men’s CVD mortality out of all variables examined (*r* = 0.53, *p* < 0.001), followed by raised blood pressure, cereals and wheat. Wheat retains high significance (*p* < 0.001) even after adjusting for health expenditure and smoking rates (Table 6), and markedly improves a regression model based on the current smoking of any tobacco product and health expenditure (from adj. *r*^2^ = 0.389 to adj. *r*^2^ = 0.497). Furthermore, daily smoking of any tobacco product approaches significance as a correlate of men’s raised blood pressure (*r* = 0.18, *p* = 0.054).

In contrast with men, women’s smoking prevalence correlates negatively with CVD risk, especially current smoking of cigarettes (*r* = −0.48, *p* < 0.001 with raised blood pressure; *r* = −0.40, *p* < 0.001 with CVD mortality). As already explained in our previous paper [14], this sex-related discrepancy in results must be ascribed to the much lower smoking prevalence in women (11.5%) than in men (34.1%), combined with the fact that women smoke mainly in wealthy countries with the high occurrence of factors that emerge as protective (compare Figures S30–S33).

These results show that smoking is a very significant confounder of CVD mortality in men, but not in women. In fact, current smoking of cigarettes is the strongest and most robust correlate of men’s CVD mortality in 39 European countries (*r* = 0.86, *p* < 0.001). However, these relationships are much weaker outside Europe, despite a comparably high smoking prevalence. For example, the association between men’s CVD mortality and the current smoking of any tobacco product in 76 non-European countries is relatively negligible (*r* = 0.36, *p* = 0.002). This case again supports the idea that men’s CVD statistics from non-European countries should be viewed as problematic. In any case, adjusting for health expenditure and smoking highlights alcohol and potatoes as the dietary factors most strongly associated with men’s CVD risk (Table 6).

Besides the negative role in relation to CVDs, smoking can also contribute to a lower prevalence of obesity which is an effect notoriously known from observational studies [30]. This explains why low rates of men’s obesity were positively tied with CVD risk in Europe in our previous paper (unlike women’s obesity) [14]. In the present study, current smoking of any tobacco product has only a slightly negative relationship with men’s obesity (*r* = −0.10, *p* = 0.31), and has a weak, positive correlation with obesity rates in women (*r* = 0.33, *p* < 0.001) which further illustrates the insignificance of women’s smoking as a health indicator at the ecological level.

The frequency of physical activity was self-reported and available from 123 countries. It is also a very generalized and ambiguous term. In theory, the percentage of physically inactive men and women should correlate positively with CVD risk, but the opposite is true (Table S11). However, Figures S34 and S35 show that a slight positive tendency of this sort may exist in Muslim countries, which have the highest levels of physical inactivity in the world and the highest rates of obesity.

### 3.3. Consistency of Findings across Regions, Sex and Health Expenditure Level

If we compare the frequency of significant correlates across five predefined regions in both sexes (see Table 2 and Table 3), carbohydrate intake (primarily from cereals and wheat in particular) is always most consistently associated with the risk of raised blood pressure and CVD mortality (Table 8). In the case of raised blood glucose, indicators of obesity occupy first place, followed by refined sugar and wheat.

As already shown above, health expenditure is an unlikely confounder of raised blood pressure. The fact that it is the most consistent correlate (nine times) must be ascribed to the eccentrically high health expenses in highly developed countries which create significantly negative, but visually weak correlations across multiple regions. Furthermore, state-organized activities aimed at CVD prevention routinely have a limited effect [31] and usually target smoking, alcohol and the lowering of blood cholesterol (via the decrease of saturated fat intake), which is at best questionable, because saturated fat actually improves key CVD parameters, such as HDL (high-density lipoprotein)-cholesterol and triglyceride levels [27]. All these lifestyle changes would also be reflected in our data and could not work as hidden confounding factors.

The comparison of countries divided according to health expenses (Table 4 and Table 5) shows similar trends, with a single eccentric exception in the category 500–2000 USD per capita where animal products (especially dairy) appear among the strongest positive correlates, together with potatoes, alcoholic beverages and sunflower oil. In most categories, we also observe a positive connection of CVD risk with distilled beverages (in men) and sunflower oil (in both sexes) which resembles our results in Europe. These positive correlations are apparently driven only by European countries in which the consumption of these two food items reaches a sufficient level (Figures S12, S13, S18, S19 and S36–S39).

In the age category above 2000 USD per capita, very few significant correlations can be found. This is mainly due to the outlier positions of Japan and South Korea that consume more carbohydrate sources than other developed countries, but achieve the same CVD statistics. Because health expenditure above 2000 USD per capita has only a negligible effect on CVD mortality, it would be interesting to examine what variables come to the foreground within the 31 countries in this group. Twenty-three of these countries are from Europe, six from North Africa, Asia and Oceania, and two from America. Although the *r*-values are not always significant, the order of variables is clearer and more meaningful than in the total sample—with raised blood pressure, raised blood glucose and carbohydrate sources as positive correlates, and life expectancy, fat, protein and major animal products as negative correlates (Table 9).

Adjusted partial correlations (Table 6) show that men’s raised blood pressure is most strongly associated with alcohol, and men’s CVD mortality with potatoes, but the foodstuffs most consistently correlated with men’s CVD risk are potatoes, sunflower oil and dairy products. In women, only % CC energy and % SRC energy and plant protein always retain significance. Besides that, we observe a sex-related discrepancy similar to the category of health expenditure of 500–2000 USD per capita, with animal products correlating positively with men’s CVD risk, but negatively with women’s risk. However, there are several foodstuffs that reach significance in both sexes, and the direction of their relationship is the same. Above all, it is fruits and rice that correlate negatively with all eight dependent variables. Oranges and mandarins, and fish and seafood reach seven significant correlations. The most consistent positive correlate is sunflower oil (seven-times), followed by wheat, potatoes and milk (six-times).

### 3.4. Factor Analysis

**Figure 8:**
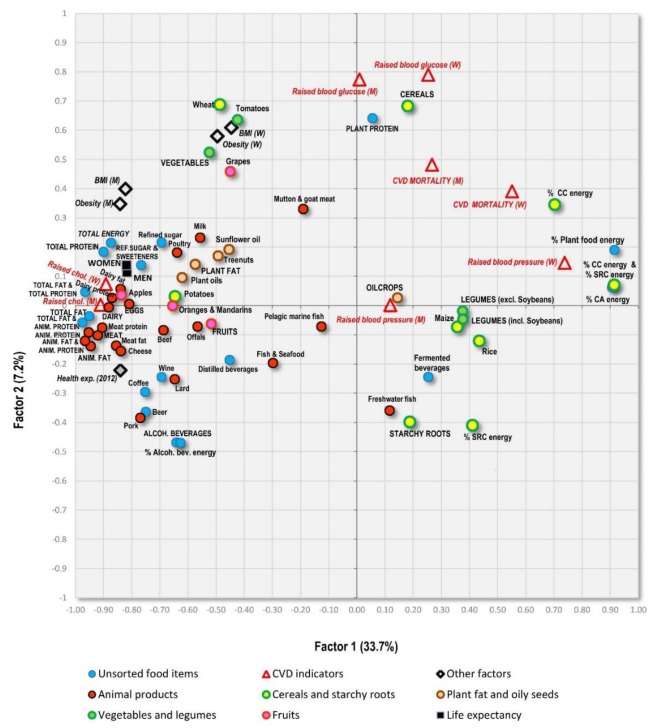
Factor analysis including 75 variables in 158 countries (Factor 1 vs. Factor 2) explaining 40.9% variability; Abbreviations: % CC energy = the mean proportion of carbohydrate energy from cereals; % SRC energy = the mean proportion of carbohydrate energy from starchy roots; % CA energy = the mean proportion of energy from carbohydrates and alcohol.

**Figure 9:**
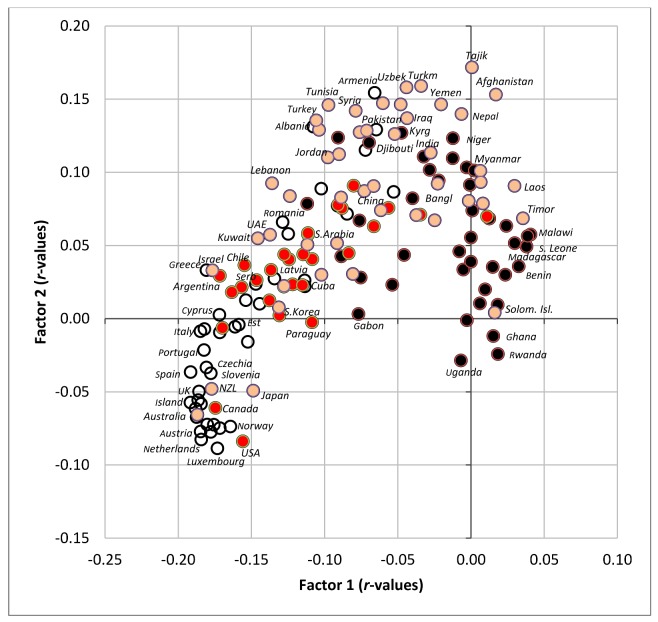
Projection of 158 countries on the factor plane of Figure 8.

Factor analyses are perhaps the best tool for the examination of multicollinearity, because they can graphically visualize mutual relationships among a large amount of variables. They were performed with 75 variables and all 158 countries. Factor 1 explains the largest proportion of variability (33.7%) and divides the countries according to the consumption of fat/protein/animal foods (which are accompanied by low CVD risk) and carbohydrates/plant foods (which are accompanied by high CVD risk) (Figure 8). These extremes are represented by countries, such as Iceland, Spain and Finland on one hand, and Malawi, Sierra Leone and Madagascar on the other (Figure 9).

Factor 2 explains 7.2% variability and divides the countries mainly according to the consumption of alcoholic beverages/pork/starchy roots on one hand, and cereals/women’s obesity on the other hand. The first extreme is represented especially by Luxembourg, the USA and the Netherlands, and the second extreme by Muslim countries (Tajikistan, Turkmenistan, Uzbekistan). As a result, Factor 2 highlights the main food items linked to high CVD risk in the Northeastern section of Figure 8: cereals and a high proportion of carbohydrates in the diet. The opposite, Southwestern section is dominated by a diet typical of wealthy ‘Western’ countries, consisting of animal products, alcohol and fruits, accompanied by high health expenditure. However, long life expectancy is more closely associated with food items such as dairy products, lean meat (poultry), eggs, refined sugar and sweeteners, various plant fat sources, and generally a high consumption of fat and protein.

**Figure 10:**
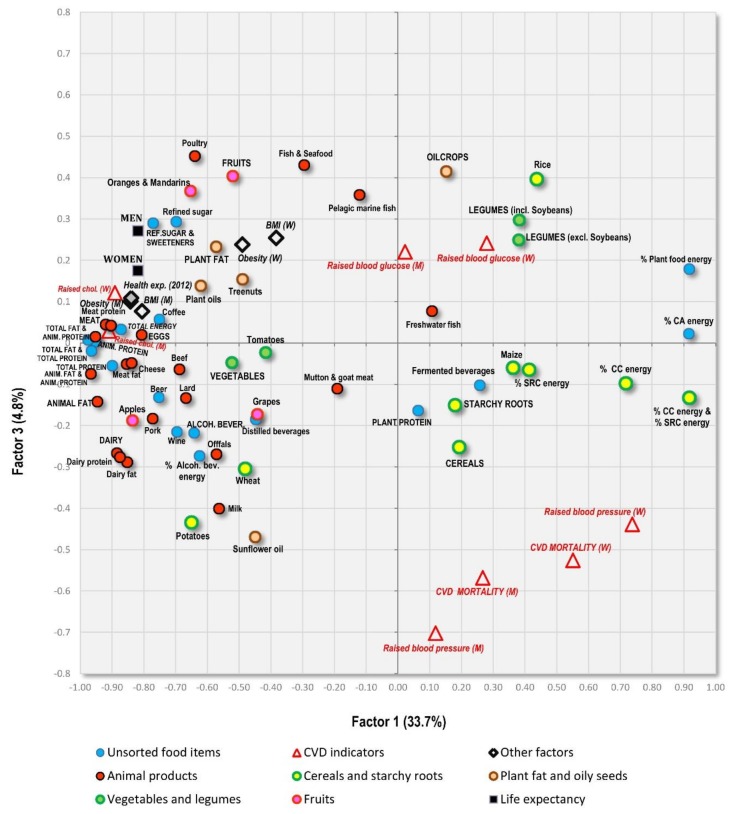
Factor analysis including 75 variables in 158 countries (Factor 1 vs. Factor 3) explaining 38.5% variability; Abbreviations: % CC energy = the mean proportion of carbohydrate energy from cereals; % SRC energy = the mean proportion of carbohydrate energy from starchy roots; % CA energy = the mean proportion of energy from carbohydrates and alcohol.

**Figure 11:**
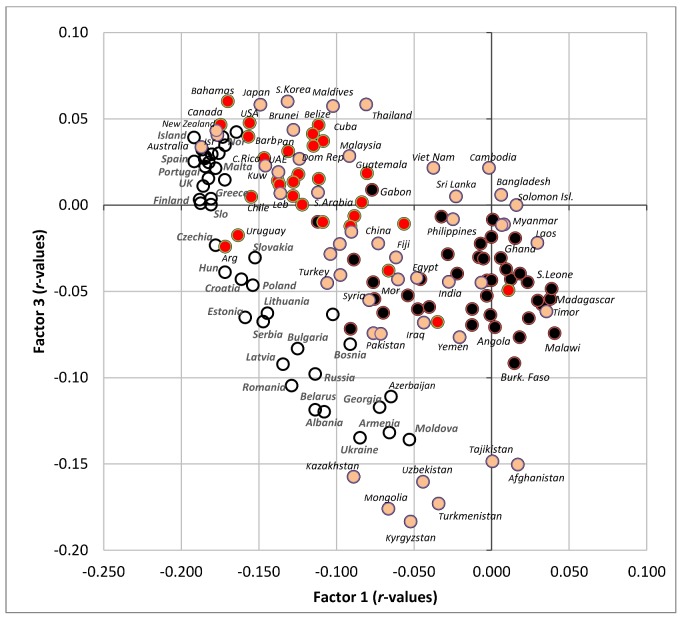
Projection of 158 countries on the factor plane of Figure 10.

Factor 3 explains only 4.8% variability (Figure 10). The Southeastern part of the graph highlights countries with high CVD mortality/high prevalence of raised blood pressure, but only a moderate/low prevalence of raised blood glucose (Figure 11). This combination is typical of many poor developing countries with a high proportion of carbohydrates in the diet, but otherwise suboptimal nutrition, as already shown in Figure 7C,D. The opposite, Northwestern part of the graph includes a diet rich in fruits, meat, eggs, coffee, refined sugar and sweeteners, and plant fat sources. This food composition is again typical mainly of the wealthiest countries of the Western world, but partly even East Asia, the Near East and Latin America. These countries are also characterized by high life expectancy, high health expenditure and higher rates of obesity.

Additional factor analyses are displayed in Figures S40–S45. When only 92 wealthier countries with health expenses above 500 USD per capita are selected (Figures S40 and S41), the position of individual items is apparently closer to the situation that we previously documented in Europe, with a ‘Mediterranean-like’ dietary pattern standing in opposition against CVDs. Figure S42 visualizes the association of men’s raised blood pressure with animal products in the category of health expenditure of 500–2000 USD per capita. However, it also shows that men’s raised blood pressure is found among sunflower oil, potatoes and alcoholic beverages, which our study repeatedly identifies as being foodstuffs associated with CVD risk. Interestingly, women’s raised blood pressure and CVD mortality lie midway between sunflower oil and carbohydrate sources. Figure S43 displays a factor analysis of 115 countries, after the inclusion of smoking, and clearly shows the close connection between men’s smoking and men’s CVD mortality.

An alternative factor analysis that only works with 116 non-European countries (Figure S44) identifies a group of variables associated with low rates of raised blood glucose and high rates of undernutrition in the Southeastern section (rice, maize, legumes, starchy roots, freshwater fish, etc.). The same variables also emerged in Table 8. Figure S45 includes only 51 non-European countries with health expenditure above 500 USD per capita and displays a similar pattern, except for raised blood glucose which moves to the Northeastern section, close to CVD mortality and raised blood pressure. Apparently, this is due to the exclusion of impoverished countries in which low blood glucose is determined by undernutrition.

In both these non-European comparisons, CVD indicators stand in stronger opposition to animal products and not to the ‘Mediterranean-like’ pattern. The most likely explanation is that some components of the ‘Mediterranean diet’ (especially dairy products) are not consumed very frequently outside Europe. However, a thing that both these nutritional styles have in common is the low proportion of carbohydrates.

### 3.5. Penalized Regression Analyses

Three penalized regression models (ridge, LASSO, elastic net) were computed in the case of women’s raised blood pressure, CVD mortality and raised blood glucose. They included 64 variables (60 food items, mean BMI, obesity, raised cholesterol, health expenditure), and their frequency in these regression models (among the top 10 variables with the highest absolute *beta* coefficients) is displayed in Table 10. Variables that appeared in these models at least twice are further divided according to the sign of their *beta* coefficients (positive/negative), which usually agrees with their *r*-values in Pearson correlations.

Because these models select common denominators out of a large number of mutually correlated variables, they can identify proxies for certain dietary patterns which may not necessarily have causal relationships to CVD risk. Indeed, the causal roles of some items (alcohol, pork, pelagic marine fish) are again unlikely, but the results generally do not differ from those based on other statistical methods in this study, with various carbohydrate sources (mainly cereals) being the variables most consistently associated with CVD risk. Penalized regression analyses performed with 92 countries (health expenditure above 500 USD per capita) produce somewhat different results than the whole sample analyses, but there are several variables emerging in both analyses, in all six regression models: fish and seafood, and oranges and mandarins in the case of raised blood pressure; sunflower oil in the case of CVD mortality; and mean BMI in the case of raised blood glucose.

### 3.6. Temporal Changes of Correlation Coefficients

The examination of long-term correlations between CVD indicators and food supply is the most meaningful in the category of health expenditure of 500–2000 USD per capita that includes 61 countries (18 countries from Europe). It is here where variables with small consumption rates (distilled beverages and sunflower oil) are the most prominent, and hence, their possible spurious association with CVDs can best be observed. In this country sample, we also observe eccentric positive correlations of animal products (especially dairy) with CVD risk which also emerge in partial correlations (Table 6) and strikingly differ from those found in women.

**Figure 12:**
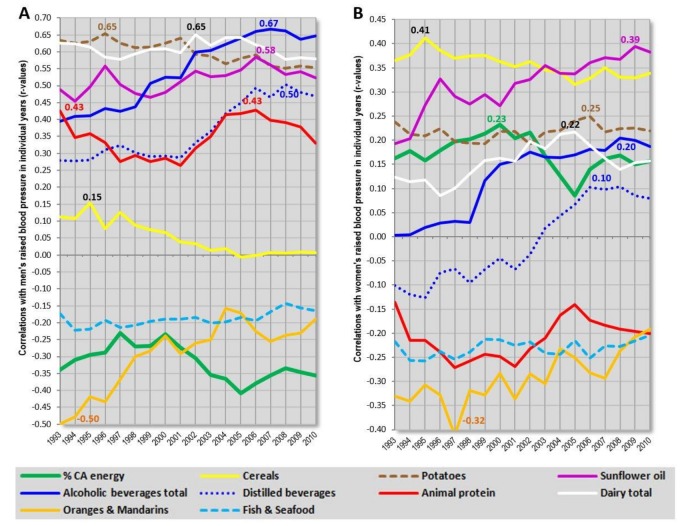
(**A**) Temporal changes in correlation coefficients (*r*-values) between men’s raised blood pressure (2010) and 10 food items. Countries with health expenditure of 500–2000 USD per capita (*n* = 61); (**B**) Temporal changes in correlation coefficients (*r*-values) between women’s raised blood pressure (2010) and 10 food items. Countries with health expenditure of 500–2000 USD per capita (*n* = 61); Abbreviations: % CA energy = the mean proportion of energy from carbohydrates and alcohol.

Figure 12A,B display the relationship between the incidence of raised blood pressure (2010) and 10 food items from the period 1993–2010. At first glance, this comparison shows that dairy products in this sample are associated with potatoes. Provided that the level of significance in the regression slope test is set at *p* > 0.05, the trend lines of potatoes and dairy do not reach particularly high *p*-values (*p* = 0.001 in men, *p* = 0.045 in women), but these two food items are quite strongly correlated even at the level of mean consumption (*r* = 0.65, *p* < 0.001), and cluster together in factor analyses (see especially Figure S42). Because potatoes serve as a substrate for the production of distilled beverages, and, according to our experience, they often emerge in the context of alcohol in ecological comparisons, it is very likely that they reflect the consumption of homemade distilled alcohol in the former USSR. In fact, countries consuming large amounts of alcohol are largely identical with those consuming the most dairy and potatoes in this sample (compare Figures S46–S49). Furthermore, potatoes are also sources of high-glycaemic starch, and hence, their position as a CVD risk factor would be potentiated.

The much stronger association of alcoholic beverages and potatoes with men’s raised blood pressure would definitely make good sense because men are more frequent alcohol drinkers than women. At the same time, the potentially protective nature of dairy products is not mutually exclusive with their positive, spurious correlation with alcohol-related men’s CVD prevalence because heavy alcohol drinkers are unlikely to consume large amounts of milk or even other dairy. The higher role of alcohol in men would also explain, why other potential CVD triggers (carbohydrates) recede to the background. In contrast, women’s raised blood pressure correlates most strongly with cereals and sunflower oil. Because their trend lines are completely different (*p* < 0.001), and both these items are unrelated, even at the level of mean consumption (*r* = 0.19, *p* = 0.14), this picture supports the causal role of sunflower oil. Nevertheless, if more accurate data on men’s CVD prevalence from non-European countries were available, the significance of carbohydrate sources would probably be much stronger even in men.

Another mutual link can be observed between animal protein, sunflower oil, and alcoholic beverages, but only the slopes of the former two in men, and the latter two in women reach the level of significance (*p* > 0.05). 

Also noteworthy is the fact that the trend lines of sunflower oil and alcohol are cumulative and peak several years before the collection of raised blood pressure statistics. This indicates an acute effect which would certainly not be surprising in the case of alcohol binge drinking which is a serious social problem in the former USSR. In contrast, the trend lines of carbohydrates (especially cereals) tend to increase with increasing time which points to a chronic effect.

An additional analysis with 91 countries (health expenditure above 500 USD per capita) shows virtually the same inter–item relationships and trends (Figures S50 and S51). Although, even here, the long-term connection between dairy and potatoes in men is less persuasive when expressed statistically (*p* < 0.001), it is almost perfectly linear in women (*p* = 0.93).

## 4. Discussion

To our knowledge, the present study is the first that has compared the complete global statistics of CVD prevalence with the nutrition statistics from the FAOSTAT database. The results show that the contemporary CVD statistics have certain limitations that must be taken into account during the interpretation of the results. In particular, men’s statistics of CVD mortality seem to be very unreliable. In addition, there are some specific confounding factors, especially the religious ban on alcohol and pork in Muslim countries, which simultaneously consume the highest amount of cereals and wheat in the world, and suffer from very high obesity rates. Besides this, the analysis of potential CVD risk factors is not complete, due to the lack of data on smoking prevalence (which was limited to 115 countries). Salt (sodium) consumption could be another important risk factor, but these statistics were analyzed elsewhere [28] and are based only on urinary excretion or estimated dietary intake from 66 countries. Therefore, our study should be taken mainly as a sort of pioneering one in this regard, and due to its ecological (country-level) methodology, it should primarily be viewed as descriptive.

Still, after an exhaustive analysis, we can say that in all the statistical comparisons that have been made, the indicators of CVDs always show the most consistent association with high carbohydrate consumption, especially in the form of high-glycaemic cereals, in particular wheat. Other suspect variables are alcohol (mainly in its distilled form) and sunflower oil, but their roles are limited to Europe where their consumption rates are sufficiently high.

Although the ecological design cannot prove causality at the level of individuals, the results can be very beneficial when combined with findings based on different methodologies because they overcome their own specific drawbacks (the accuracy of input data in observational studies, the short-term character of controlled interventional studies). It is true that the connection between CVDs and carbohydrates has not been universally accepted yet, but both epidemiological and mechanistic evidence is growing, and this fundamental problem has attracted wide public attention. Actually, our results—including the link between raised cholesterol and *lower* CVD risk—have been almost perfectly replicated by the recent global PURE (Prospective Urban and Rural Epidemiological) study [32,33], the most shared scientific work in the world in 2017 [34]. Excessive alcohol drinking is also recognized as an important factor of CVD risk [35] and can explain the recent epidemic of CVDs in men from the former USSR republics [20].

The role of sunflower oil is less clear. Even its leading position in the penalized regression models is not a guarantee of a true causal relationship because these models may sometimes select a mere proxy (common denominator) for certain dietary patterns. In fact, sunflower oil is consumed mainly in Eastern European countries, where both carbohydrate consumption and alcohol drinking reach the highest rates in Europe. Still, a meaningful rationale of this finding does exist and may lie in the high content of linoleic acid in sunflower oil and its proatherogenic properties [36]. This problem is a subject of ongoing debate [37].

The list of negative correlates is less consistent in details because it may differ according to regional dietary habits. It is quite understandable that some foodstuffs which have a strong role in Europe (dairy products, but also tree nuts) do not reach such a status elsewhere because their consumption rates are relatively negligible. However, the factor analyses repeatedly display a very similar picture, with CVD indicators standing in opposition against high fat/protein consumption and animal products. The most frequently highlighted foodstuffs (fruits, oranges and mandarins, fish and seafood) also emerged in the sample of 42 European countries [14]. Because the spectrum of foods used in this study was practically exhaustive, these relationships cannot be explained by any hidden dietary confounder. Provided that the ‘saturated fat hypothesis’ is correct, we would have to assume that in every part of the world, some other exogenous factor (most likely healthcare) is always able to completely reverse the relationship between nutrition and CVD. While this might apply to CVD mortality, where the effect of healthcare is the most direct, it cannot relate to the statistics of raised blood glucose that include the use of medications. The fact that we confirmed the well-established connection between raised blood glucose and obesity/high-glycaemic foods demonstrates that the ecological methodology produces biologically valid and meaningful results, provided that good quality data are available.

The statistics of raised blood pressure also reflect CVD prevalence, but they do not include the use of blood pressure-lowering drugs. This may skew the true prevalence rates of this fundamental CVD indicator. Nevertheless, our data from Europe show that when the use of medications is taken into account, the observed trends are even stronger, particularly in men. Furthermore, the most precise statistics (that are available from a limited number of countries) show that the prevalence of raised blood pressure is higher (and increasing) in low-income countries [38]. Therefore, we expect that when more objective data on raised blood pressure or CVD events are available, the geographical pattern of CVD prevalence will be confirmed.

Another noteworthy finding is the disproportionately lower prevalence of (women’s) raised blood pressure in Asian countries consuming rice as the main cereal in their diet. Interestingly, this finding was supported by adjusted partial correlations (Table 6), and manifests even in factor analyses (Figure S41). It is therefore tempting to speculate that rice offers some health advantage, when compared with wheat and other cereals. In the available literature, there is some limited evidence that the beneficial effects of rice—at least in comparison with wheat—could be real [39,40,41] and this problem merits further research. On the other hand, the seemingly protective role of refined sugar in some analyses has a more prosaic explanation. Although its glycaemic index (65) is lower than in white wheat bread (75) or boiled potatoes (77) [42], even more important is the fact that its mean consumption (9.6% of total energy) lags far behind high glycaemic plant starches (% CC energy & % SRC energy) (42.6%), and these two variables have a strongly negative relationship (*r* = −0.76, *p* < 0.001).

## 5. Conclusions

In summary, our study shows that the divergent association of basic nutritional components with CVD indicators, which we found in our previous study [14], holds true both for Europe and regions outside Europe. More convincing results will be possible when more precise data on CVD prevalence are available. Because self-reported dietary questionnaires from observational studies are increasingly perceived as the dead end of nutritional science [43], such high-quality data are urgently needed. Results of detailed ecological analyses such as the present one can subsequently constitute a very useful contribution to the recent debate regarding the changing paradigm in cardiology.

## Figures and Tables

**Table 1:** Variables used in this study.

Variables Used in the Main Analysis	Description	Countries (*n*)	Source
Food supply (FAOSTAT, 1993–2011)	60 food items	175	FAO [17]
Raised blood pressure (2010)	>18 years, age-standardized; systolic ≥ 140 or diastolic ≥ 90 (mmHg)	192	WHO [19]
Raised cholesterol (2008)	>25 years, age-standardized; ≥5.0 mmol/L	189	WHO [19]
CVD mortality (2012)	Age-standardized, per 100,000 population	172	WHO [19]
Raised blood glucose (2010)	>18 years, age-standardized; ≥126 mg/dL (7.0 mmol/L) history of diabetes, or on medication	192	WHO [19]
Obesity (BMI > 30 kg/m^2^) (2010)	>18 years, age-standardized	192	WHO [19]
Mean BMI (kg/m^2^) (2010)	>18 years, age-standardized	192	WHO [19]
Health expenditure per capita (2012)	PPP, constant 2011 international USD	188	World Bank [23]
Life expectancy (2012)	Life expectancy at birth	200	World Bank [23]
All variables used		158	
Variables Used in the Supplementary Analysis	Description	Countries (*n*)	Source
Insufficiently active adults (2010)	>18 years, age-standardized	123	WHO [19]
Smoking of any tobacco product (2013)	>15 years, age-standardized	115 (both sexes)	WHO [19]
Daily smoking of any tobacco product (2013)	>15 years, age-standardized	115 (both sexes)	WHO [19]
Smoking of cigarettes (2013)	>15 years, age-standardized	115 (both sexes)	WHO [19]
Daily smoking of cigarettes (2013)	>15 years, age-standardized	115 (both sexes)	WHO [19]

Abbreviations: FAO = Food and Agriculture Organization; FAOSTAT = Food and Agriculture Organization Corporate Statistical Database; WHO = World Health Organization; CVD = cardiovascular disease; BMI = Body mass index; PPP = Purchasing power parity; USD = the United States dollar.

**Table 2:** Pearson linear correlations in men: regional differences.

Variable	Raised Blood Pressure (Men)					CVD Mortality (Men)					Raised Blood Glucose (Men)				
Region	Europe	World Outside Europe	North Africa & Asia & Oceania	America	Sub-Saharan Africa	Europe	World Outside Europe	North Africa & Asia & Oceania	America	Sub-Saharan Africa	Europe	World Outside Europe	North Africa & Asia & Oceania	America	Sub-Saharan Africa
*n* (countries)	42	116	47	29	40	42	116	47	29	40	42	116	47	29	40
Fruits (Total)	−0.60	−0.35	−0.18	−0.01	−0.34	−0.78	−0.29	−0.38	−0.19	−0.02	−0.44	0.08	0.20	0.06	−0.15
Apples	−0.18	−0.37	−0.13	−0.18	−0.09	−0.39	−0.07	−0.19	−0.25	0.04	−0.43	0.33	0.13	0.28	0.58
Grapes	−0.12	−0.24	0.05	−0.33	−0.15	−0.05	0.25	0.20	−0.29	0.10	0.05	0.40	0.25	0.22	0.51
Oranges and Mandarins	−0.58	−0.37	−0.13	0.12	−0.03	−0.74	−0.26	−0.42	−0.12	0.04	−0.34	0.21	0.29	0.20	0.31
Alcoholic Beverages (Total)	−0.19	−0.13	−0.55	−0.22	−0.15	−0.59	−0.29	−0.37	−0.31	0.24	−0.47	−0.25	−0.31	0.00	0.05
Beer	−0.14	−0.39	−0.51	−0.32	0.01	−0.48	−0.38	−0.38	−0.31	0.07	−0.38	−0.09	−0.29	−0.01	0.67
Distilled Beverages	0.47	−0.33	−0.33	0.27	−0.11	0.32	−0.12	−0.12	0.28	−0.09	0.16	−0.11	−0.40	0.25	0.21
Fermented Beverages	0.16	0.20	−0.51	−0.42	−0.17	−0.13	−0.02	−0.28	−0.20	0.20	−0.05	−0.23	−0.13	−0.16	−0.25
Wine	−0.33	−0.08	−0.25	0.21	0.29	−0.58	−0.16	−0.14	−0.13	0.24	−0.44	−0.02	−0.16	−0.01	0.64
Coffee	−0.36	−0.45	−0.25	−0.35	0.05	−0.69	−0.36	−0.46	−0.21	0.13	−0.52	0.03	−0.02	−0.15	0.06
Refind Sugar and Sweeteners (Total)	−0.15	−0.44	−0.13	0.05	0.12	−0.34	−0.30	−0.36	−0.18	−0.17	−0.58	0.33	0.31	0.52	0.54
Refined Sugar	−0.23	−0.34	0.01	0.32	0.13	−0.25	−0.24	−0.24	−0.12	−0.16	−0.55	0.37	0.39	0.58	0.53
Vegetables (Total)	−0.17	−0.33	−0.11	−0.21	−0.02	0.07	0.11	−0.08	−0.15	0.08	0.07	0.44	0.29	0.10	0.24
Tomatoes	−0.33	−0.13	0.21	−0.23	0.10	0.01	0.19	0.15	−0.32	0.11	0.06	0.52	0.50	−0.14	0.34
Legumes (Excluding Soybeans)	−0.08	0.10	0.00	0.08	−0.25	−0.19	−0.22	−0.23	0.00	−0.15	−0.04	−0.16	0.31	−0.08	−0.44
Legumes (Including Soybeans)	−0.08	0.02	−0.23	0.08	−0.28	−0.21	−0.27	−0.43	−0.02	−0.16	−0.05	−0.18	0.21	−0.10	−0.46
Cereals (Total)	0.29	0.02	0.29	−0.08	0.14	0.61	0.37	0.37	0.02	0.09	0.59	0.32	0.20	0.12	0.15
Maize	0.15	0.08	−0.04	0.00	−0.50	0.24	−0.19	−0.06	−0.20	−0.08	0.19	−0.24	−0.13	−0.02	−0.13
Rice	−0.36	−0.24	−0.43	0.00	0.26	−0.36	−0.04	−0.35	0.40	0.31	−0.22	−0.16	−0.45	−0.13	0.03
Wheat	0.01	−0.08	0.49	−0.05	0.19	0.38	0.44	0.50	−0.07	−0.16	0.51	0.59	0.47	0.20	0.59
Starchy Roots (Total)	0.28	0.21	−0.17	−0.19	−0.26	0.31	−0.01	0.05	0.05	0.13	−0.04	−0.23	0.08	−0.52	−0.20
Potatoes	0.28	−0.31	0.19	−0.57	−0.44	0.31	0.09	0.25	−0.33	−0.04	−0.04	0.11	0.09	−0.14	−0.14
Oilcrops	−0.44	−0.24	−0.36	0.04	0.19	−0.33	0.06	−0.11	0.65	0.18	−0.24	0.15	0.08	0.12	0.04
Plant Oils	−0.48	−0.14	0.04	−0.32	0.27	−0.58	−0.17	−0.18	−0.35	0.05	−0.53	0.26	0.35	−0.13	0.25
Sunflower Oil	0.28	0.04	0.31	0.28	−0.17	0.40	0.17	0.33	−0.09	0.00	−0.06	0.27	0.30	−0.03	0.40
Tree Nuts	−0.62	−0.26	−0.14	−0.48	−0.17	−0.55	−0.06	−0.27	−0.08	0.07	−0.42	0.20	0.08	−0.04	−0.11
Plant Protein	0.07	0.02	0.09	−0.23	−0.12	0.37	0.22	0.11	−0.05	0.09	0.46	0.33	0.38	0.00	−0.03
Plant fat	−0.53	−0.12	−0.06	−0.31	0.32	−0.66	−0.16	−0.24	−0.30	0.14	−0.51	0.32	0.39	0.00	0.29
% CC Energy	0.34	0.12	0.11	0.03	0.11	0.71	0.31	0.22	0.27	0.02	0.73	−0.02	−0.29	−0.06	−0.01
% SRC Energy	0.41	0.28	−0.18	−0.15	−0.22	0.49	−0.02	0.03	0.13	0.12	0.10	−0.27	0.03	−0.56	−0.21
% CC Energy and % SRC Energy	0.40	0.33	0.03	−0.04	−0.16	0.77	0.29	0.22	0.31	0.20	0.72	−0.24	−0.27	−0.31	−0.32
% CA energy	0.47	0.21	−0.08	0.09	−0.48	0.80	0.10	0.05	0.30	−0.05	0.68	−0.28	−0.22	−0.08	−0.42
% Alcoholic Beverages Energy	0.11	−0.17	−0.50	0.16	−0.26	−0.35	−0.27	−0.27	−0.01	0.13	−0.31	−0.30	−0.40	0.09	0.02
% Plant Food Energy	0.21	0.30	−0.02	−0.06	−0.31	0.67	0.10	−0.01	0.17	0.10	0.65	−0.13	0.06	−0.15	−0.49
Fish and Seafood	−0.33	−0.32	−0.47	−0.15	0.23	−0.51	−0.15	−0.39	0.33	0.13	−0.24	0.09	−0.13	0.43	0.44
Pelagic Marine Fish	−0.08	−0.15	−0.27	0.00	0.26	−0.21	−0.09	−0.18	−0.20	0.11	−0.12	0.14	0.01	0.52	0.40
Freshwater Fish	−0.30	−0.09	−0.33	−0.38	0.00	−0.34	−0.11	−0.27	−0.16	−0.03	−0.32	−0.29	−0.45	−0.39	−0.14
Meat (Total)	−0.37	−0.34	−0.02	−0.05	0.31	−0.74	−0.20	−0.20	−0.18	0.01	−0.56	0.22	0.11	0.15	0.72
Beef	−0.40	−0.24	−0.01	0.02	0.10	−0.47	−0.14	0.06	−0.22	−0.13	−0.46	−0.02	−0.07	−0.18	0.36
Pork	−0.15	−0.51	−0.63	−0.22	0.26	−0.57	−0.30	−0.35	−0.25	0.19	−0.52	−0.20	−0.49	−0.10	0.29
Poultry	−0.26	−0.38	−0.05	−0.01	0.00	−0.54	−0.29	−0.44	0.01	0.14	−0.29	0.39	0.33	0.55	0.70
Mutton and Goat Meat	−0.38	0.23	0.47	0.26	0.45	−0.29	0.34	0.31	0.04	−0.14	0.02	0.36	0.29	0.26	0.26
Meat Fat	−0.41	−0.28	0.00	0.20	0.30	−0.67	−0.05	−0.01	−0.12	0.07	−0.51	0.18	−0.02	0.15	0.58
Meat Protein	−0.42	−0.29	0.04	−0.02	0.30	−0.75	−0.18	−0.16	−0.18	−0.01	−0.56	0.23	0.13	0.13	0.71
Dairy (Total, Excluding Butter)	−0.42	−0.19	0.36	−0.24	0.32	−0.61	0.01	0.21	−0.19	−0.22	−0.63	0.25	0.22	−0.01	0.34
Cheese	−0.51	−0.34	−0.08	−0.31	0.20	−0.73	−0.27	−0.30	−0.24	−0.17	−0.60	0.17	0.09	0.20	0.26
Milk	0.10	−0.02	0.44	−0.02	0.35	0.26	0.20	0.50	−0.13	−0.20	0.05	0.20	0.19	−0.18	0.29
Dairy Fat	−0.46	−0.12	0.39	−0.20	0.36	−0.55	0.05	0.28	−0.22	−0.20	−0.52	0.26	0.26	0.04	0.27
Dairy Protein	−0.35	−0.16	0.38	−0.17	0.35	−0.58	0.03	0.21	−0.17	−0.21	−0.61	0.27	0.24	0.04	0.33
Eggs (Total)	0.08	−0.52	−0.28	−0.18	−0.07	−0.19	−0.29	−0.40	−0.51	0.09	−0.55	0.24	0.14	−0.15	0.45
Lard	0.02	−0.36	−0.25	−0.13	0.27	−0.26	−0.25	−0.11	−0.21	−0.09	−0.35	−0.06	−0.23	0.14	0.11
Offal	0.08	0.03	0.17	0.07	0.45	−0.16	−0.01	0.01	−0.08	−0.08	−0.19	0.09	0.00	−0.24	0.50
Animal Fat	−0.38	−0.36	0.01	−0.05	0.40	−0.71	−0.10	−0.04	−0.24	−0.09	−0.71	0.24	0.07	0.14	0.51
Animal Protein	−0.42	−0.37	−0.09	−0.10	0.41	−0.75	−0.17	−0.23	−0.15	−0.06	−0.61	0.28	0.10	0.17	0.71
Animal Fat and Animal Protein	−0.41	−0.37	−0.04	−0.07	0.42	−0.75	−0.14	−0.14	−0.20	−0.08	−0.69	0.26	0.09	0.16	0.64
Total Fat	−0.52	−0.31	−0.04	−0.20	0.49	−0.80	−0.16	−0.18	−0.31	0.06	−0.72	0.35	0.30	0.09	0.53
Total Protein	−0.44	−0.32	−0.03	−0.18	0.23	−0.67	−0.03	−0.14	−0.16	0.02	−0.48	0.43	0.31	0.16	0.51
Total Fat and Animal Protein	−0.50	−0.34	−0.06	−0.16	0.51	−0.81	−0.17	−0.21	−0.26	0.02	−0.71	0.33	0.24	0.12	0.66
Total Fat and Total Protein	−0.52	−0.33	−0.04	−0.20	0.44	−0.81	−0.11	−0.17	−0.26	0.05	−0.69	0.40	0.32	0.12	0.61
Total Energy	−0.43	−0.37	−0.10	−0.17	0.12	−0.65	−0.07	−0.20	−0.23	0.23	−0.63	0.47	0.42	0.18	0.44
Raised Cholesterol (Men)	−0.40	−0.47	−0.14	−0.16	−0.04	−0.79	−0.16	−0.32	−0.45	−0.16	−0.61	0.45	0.29	0.10	0.62
Raised Blood Pressure (Men)						0.66	0.30	0.63	0.17	0.29	0.29	0.10	0.51	0.41	0.45
CVD Mortality (Men)	0.66	0.30	0.63	0.17	0.29						0.47	0.31	0.25	0.02	0.25
Raised Blood Glucose (Men)	0.29	0.10	0.51	0.41	0.45	0.47	0.31	0.25	0.02	0.25					
Mean BMI (Men)	−0.11	−0.36	0.27	−0.05	0.18	−0.38	0.00	0.14	−0.41	0.17	0.17	0.63	0.72	0.55	0.77
Obesity (Men)	−0.22	−0.31	0.26	−0.11	0.21	−0.64	−0.03	0.05	−0.33	0.13	−0.26	0.65	0.74	0.43	0.83
Life Expectancy (Men)	−0.72	−0.65	−0.45	−0.22	0.15	−0.96	−0.26	−0.67	−0.61	−0.19	−0.48	0.30	0.05	−0.03	0.18
Health Expenditure (2012)	−0.62	−0.50	−0.33	−0.50	−0.06	−0.80	−0.32	−0.52	−0.24	0.05	−0.60	0.14	0.07	0.00	0.65
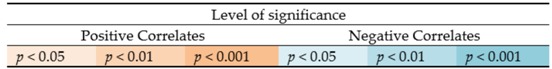															

Abbreviations: % CC energy = the mean proportion of carbohydrate energy from cereals; % SRC energy = the mean proportion of carbohydrate energy from starchy roots; % CA energy = the mean proportion of energy from carbohydrates and alcohol. The colors in the first column divide plant foods, animal foods, general food items and other variables.

**Table 3:** Pearson linear correlations in women: regional differences.

Variable	Raised Blood Pressure (Women)					CVD Mortality (Women)					Raised Blood Glucose (Women)				
Region	Europe	World Outside Europe	North Africa & Asia & Oceania	America	Sub–Saharan Africa	Europe	World Outside Europe	North Africa & Asia & Oceania	America	Sub–Saharan Africa	Europe	World Outside Europe	North Africa & Asia & Oceania	America	Sub–Saharan Africa
*n* (countries)	42	116	47	29	40	42	116	47	29	40	42	116	47	29	40
Fruits (Total)	−0.72	−0.47	−0.31	−0.17	−0.45	−0.72	−0.38	−0.44	−0.14	−0.01	−0.59	0.09	0.15	0.14	−0.19
Apples	−0.36	−0.50	−0.35	−0.55	−0.31	−0.44	−0.26	−0.25	−0.42	−0.19	−0.44	0.21	0.03	−0.06	0.72
Grapes	0.06	−0.22	0.05	−0.61	−0.37	0.13	0.11	0.19	−0.43	−0.21	0.14	0.35	0.24	−0.09	0.60
Oranges and Mandarins	−0.75	−0.52	−0.27	−0.16	−0.20	−0.74	−0.41	−0.43	−0.18	0.03	−0.57	0.23	0.23	0.17	0.47
Alcoholic Beverages (Total)	−0.49	−0.24	−0.73	−0.59	−0.15	−0.66	−0.29	−0.46	−0.43	0.06	−0.64	−0.31	−0.45	−0.37	0.05
Beer	−0.42	−0.57	−0.69	−0.55	−0.37	−0.54	−0.50	−0.47	−0.39	−0.14	−0.52	−0.17	−0.41	−0.31	0.62
Distilled Beverages	0.38	−0.48	−0.50	0.19	−0.18	0.20	−0.29	−0.22	0.29	−0.21	0.12	−0.09	−0.46	0.21	0.36
Fermented Beverages	−0.01	0.25	−0.52	−0.47	0.01	−0.22	0.10	−0.30	−0.24	0.12	−0.14	−0.24	−0.25	−0.32	−0.23
Wine	−0.48	−0.31	−0.41	−0.24	−0.14	−0.57	−0.27	−0.18	−0.28	−0.11	−0.55	−0.12	−0.26	−0.27	0.53
Coffee	−0.67	−0.59	−0.54	−0.28	−0.08	−0.72	−0.47	−0.51	−0.15	0.05	−0.69	−0.07	−0.18	−0.30	−0.03
Ref. Sugar and Sweeteners (Total)	−0.50	−0.64	−0.43	−0.10	−0.25	−0.46	−0.52	−0.46	−0.29	−0.25	−0.65	0.33	0.21	0.32	0.73
Refined Sugar	−0.49	−0.54	−0.27	0.22	−0.26	−0.39	−0.46	−0.35	−0.19	−0.26	−0.55	0.40	0.31	0.51	0.70
Vegetables (Total)	0.07	−0.38	−0.25	−0.51	−0.36	0.18	−0.05	−0.11	−0.29	0.00	0.17	0.33	0.20	−0.09	0.06
Tomatoes	−0.10	−0.17	0.12	−0.47	−0.33	0.09	0.04	0.10	−0.38	−0.07	0.15	0.47	0.49	−0.40	0.28
Legumes (Excluding Soybeans)	−0.01	0.22	0.16	0.40	0.09	−0.06	−0.12	−0.24	0.10	−0.09	−0.14	−0.11	0.32	−0.02	−0.36
Legumes (Including Soybeans)	−0.02	0.13	−0.10	0.37	0.06	−0.07	−0.18	−0.44	0.08	−0.10	−0.15	−0.15	0.17	−0.04	−0.36
Cereals (Total)	0.62	0.17	0.50	−0.03	0.31	0.67	0.33	0.43	−0.08	0.10	0.77	0.34	0.30	0.04	0.26
Maize	0.39	0.19	0.11	0.20	−0.11	0.35	−0.07	0.05	−0.16	−0.10	0.32	−0.13	−0.10	−0.09	0.26
Rice	−0.37	−0.13	−0.21	0.21	0.07	−0.35	−0.04	−0.34	0.45	0.32	−0.27	−0.16	−0.39	0.15	−0.20
Wheat	0.30	−0.15	0.39	−0.42	−0.31	0.47	0.23	0.49	−0.24	−0.36	0.62	0.55	0.49	0.00	0.62
Starchy Roots (Total)	0.19	0.25	−0.02	−0.28	−0.26	0.09	0.12	−0.04	0.03	0.14	0.03	−0.24	0.12	−0.45	−0.31
Potatoes	0.19	−0.35	−0.01	−0.81	−0.23	0.09	−0.08	0.14	−0.46	−0.07	0.03	0.09	0.04	−0.31	0.06
Oilcrops	−0.38	−0.14	−0.21	0.20	0.10	−0.22	−0.09	−0.26	0.60	0.07	−0.27	0.20	0.16	0.33	−0.27
Plant Oils	−0.61	−0.29	−0.20	−0.48	−0.11	−0.60	−0.25	−0.24	−0.37	−0.04	−0.63	0.10	0.23	−0.45	0.07
Sunflower Oil	0.36	−0.07	0.20	−0.07	−0.27	0.44	0.03	0.24	−0.16	−0.12	0.06	0.27	0.32	−0.22	0.61
Tree Nuts	−0.59	−0.26	−0.27	−0.45	−0.06	−0.50	−0.13	−0.32	−0.09	0.20	−0.43	0.12	−0.01	−0.03	−0.11
Plant Protein	0.38	0.17	0.21	−0.10	0.18	0.44	0.22	0.12	−0.10	0.10	0.56	0.32	0.40	−0.03	0.06
Plant fat	−0.67	−0.23	−0.25	−0.42	0.02	−0.67	−0.23	−0.32	−0.33	0.06	−0.66	0.18	0.30	−0.30	0.06
% CC Energy	0.73	0.36	0.48	0.40	0.36	0.79	0.39	0.36	0.35	0.10	0.91	0.04	−0.16	0.15	0.10
% SRC Energy	0.38	0.34	0.02	−0.10	−0.18	0.28	0.14	−0.03	0.19	0.11	0.22	−0.28	0.09	−0.38	−0.34
% CC Energy and % SRC Energy	0.77	0.62	0.48	0.33	0.24	0.82	0.50	0.34	0.41	0.31	0.92	−0.18	−0.12	−0.03	−0.35
% CA Energy	0.80	0.50	0.41	0.51	−0.22	0.83	0.29	0.16	0.44	0.06	0.88	−0.13	−0.06	0.25	−0.23
% Alcoholic Beverages Energy	−0.19	−0.31	−0.70	−0.20	−0.21	−0.44	−0.31	−0.38	−0.04	−0.03	−0.46	−0.31	−0.52	−0.19	0.12
% Plant Food Energy	0.63	0.62	0.47	0.48	0.12	0.74	0.35	0.10	0.37	0.27	0.83	−0.06	0.20	0.12	−0.56
Fish and Seafood	−0.54	−0.38	−0.53	−0.29	−0.29	−0.58	−0.24	−0.44	0.15	0.05	−0.45	0.01	−0.19	0.41	0.13
Pelagic Marine Fish	−0.28	−0.19	−0.29	−0.15	−0.19	−0.30	−0.15	−0.25	−0.26	0.04	−0.25	0.10	−0.01	0.50	0.15
Freshwater Fish	−0.46	0.00	−0.19	−0.46	−0.11	−0.44	−0.03	−0.24	−0.22	0.08	−0.40	−0.30	−0.40	−0.49	−0.24
Meat (Total)	−0.71	−0.65	−0.51	−0.54	−0.18	−0.81	−0.41	−0.28	−0.36	−0.19	−0.76	0.10	−0.07	−0.18	0.68
Beef	−0.58	−0.49	−0.38	−0.45	0.07	−0.56	−0.30	−0.04	−0.37	−0.10	−0.50	−0.11	−0.16	−0.53	0.50
Pork	−0.47	−0.64	−0.72	−0.53	−0.05	−0.63	−0.45	−0.41	−0.34	−0.16	−0.66	−0.30	−0.58	−0.35	0.14
Poultry	−0.48	−0.62	−0.42	−0.31	−0.46	−0.61	−0.47	−0.45	−0.14	−0.19	−0.50	0.31	0.16	0.40	0.61
Mutton and Goat Meat	−0.36	0.06	0.11	−0.05	0.25	−0.21	0.23	0.23	−0.10	−0.05	−0.11	0.29	0.22	0.20	0.34
Meat Fat	−0.69	−0.58	−0.44	−0.35	−0.11	−0.74	−0.29	−0.11	−0.30	−0.26	−0.71	0.08	−0.15	−0.12	0.58
Meat Protein	−0.73	−0.61	−0.46	−0.53	−0.17	−0.82	−0.38	−0.24	−0.37	−0.17	−0.75	0.10	−0.04	−0.21	0.68
Dairy (Total, Excluding Butter)	−0.63	−0.44	−0.06	−0.57	−0.06	−0.64	−0.20	0.14	−0.38	−0.26	−0.69	0.18	0.14	−0.32	0.44
Cheese	−0.76	−0.51	−0.33	−0.62	0.04	−0.77	−0.40	−0.34	−0.40	−0.11	−0.70	0.08	0.01	−0.16	0.39
Milk	0.23	−0.25	0.09	−0.26	−0.02	0.32	0.00	0.39	−0.27	−0.23	0.13	0.19	0.17	−0.31	0.39
Dairy Fat	−0.60	−0.36	0.01	−0.55	0.01	−0.51	−0.15	0.20	−0.41	−0.21	−0.58	0.21	0.19	−0.27	0.37
Dairy Protein	−0.60	−0.41	−0.03	−0.51	−0.02	−0.59	−0.18	0.15	−0.37	−0.24	−0.67	0.22	0.16	−0.23	0.42
Eggs (Total)	−0.29	−0.70	−0.63	−0.36	−0.43	−0.35	−0.48	−0.43	−0.58	−0.26	−0.55	0.10	−0.04	−0.46	0.46
Lard	−0.21	−0.51	−0.45	−0.32	0.16	−0.32	−0.41	−0.21	−0.26	−0.23	−0.40	−0.06	−0.32	0.06	0.16
Offal	−0.12	−0.25	−0.27	−0.21	0.07	−0.20	−0.13	−0.08	−0.15	−0.11	−0.23	0.00	−0.12	−0.25	0.50
Animal Fat	−0.74	−0.65	−0.47	−0.55	−0.06	−0.78	−0.36	−0.16	−0.44	−0.30	−0.84	0.14	−0.07	−0.16	0.56
Animal Protein	−0.77	−0.68	−0.59	−0.59	−0.21	−0.83	−0.41	−0.34	−0.38	−0.20	−0.80	0.14	−0.08	−0.16	0.64
Animal Fat and Animal Protein	−0.78	−0.68	−0.55	−0.58	−0.14	−0.83	−0.39	−0.26	−0.42	−0.26	−0.85	0.14	−0.08	−0.17	0.62
Total Fat	−0.82	−0.57	−0.46	−0.57	−0.02	−0.85	−0.37	−0.31	−0.45	−0.13	−0.88	0.20	0.15	−0.26	0.38
Total Protein	−0.69	−0.53	−0.40	−0.59	−0.03	−0.73	−0.25	−0.23	−0.39	−0.08	−0.64	0.30	0.17	−0.17	0.52
Total Fat and Animal Protein	−0.83	−0.64	−0.54	−0.59	−0.10	−0.87	−0.40	−0.34	−0.44	−0.17	−0.88	0.18	0.07	−0.23	0.52
Total Fat and Total Protein	−0.83	−0.58	−0.45	−0.60	−0.03	−0.86	−0.34	−0.29	−0.45	−0.12	−0.86	0.25	0.17	−0.23	0.51
Total Energy	−0.69	−0.53	−0.35	−0.51	−0.23	−0.74	−0.29	−0.31	−0.42	0.07	−0.74	0.36	0.32	−0.12	0.37
Raised Cholesterol (Women)	−0.67	−0.72	−0.52	−0.60	−0.56	−0.79	−0.45	−0.47	−0.66	−0.32	−0.78	0.38	0.19	−0.17	0.68
Raised Blood Pressure (Women)						0.86	0.61	0.68	0.48	0.45	0.71	0.06	0.53	0.43	−0.11
CVD Mortality (Women)	0.86	0.61	0.68	0.48	0.45						0.75	0.19	0.31	0.19	−0.05
Raised Blood Glucose (Women)	0.71	0.06	0.53	0.43	−0.11	0.75	0.19	0.31	0.19	−0.05					
Mean BMI (Women)	0.23	−0.36	0.12	−0.17	−0.26	0.36	−0.20	0.09	−0.27	−0.10	0.70	0.72	0.78	0.72	0.89
Obesity (Women)	0.31	−0.32	0.16	−0.19	−0.24	0.16	−0.21	0.01	−0.21	−0.01	0.38	0.72	0.80	0.62	0.85
Life Expectancy (Women)	−0.83	−0.80	−0.72	−0.52	−0.36	−0.93	−0.51	−0.62	−0.82	−0.43	−0.76	0.20	−0.15	−0.25	0.02
Health Expenditure (2012)	−0.86	−0.64	−0.69	−0.66	−0.36	−0.83	−0.45	−0.57	−0.34	−0.09	−0.75	−0.02	−0.16	−0.30	0.78
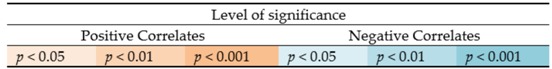															

**Table 4:** Pearson linear correlations in men: differences according to health expenditure per capita (constant 2011 international USD, 2012).

Variable	Raised Blood Pressure (Men)					CVD Mortality (Men)					Raised Blood Glucose (Men)				
Category	Total Sample	>500 USD	1000 USD	500–2000 USD	2000 USD	Total Sample	>500 USD	1000 USD	500–2000 USD	2000 USD	Total Sample	>500 USD	1000 USD	500–2000 USD	2000 USD
*n* (countries)	158	92	60	61	31	158	92	60	61	31	158	92	60	61	31
Fruits (Total)	−0.32	−0.35	−0.32	−0.39	−0.06	−0.38	−0.51	−0.38	−0.46	−0.34	−0.02	−0.12	−0.02	0.07	−0.29
Apples	0.00	0.10	0.07	0.47	0.08	−0.13	−0.12	−0.19	0.42	−0.22	−0.04	−0.34	−0.41	−0.04	−0.40
Grapes	−0.04	0.02	−0.04	0.12	0.01	0.13	0.04	−0.14	0.24	−0.04	0.17	−0.03	−0.10	0.11	−0.07
Oranges and Mandarins	−0.33	−0.34	−0.33	−0.31	−0.11	−0.41	−0.48	−0.44	−0.34	−0.28	0.05	−0.14	−0.11	0.14	−0.14
Alcoholic Beverages (Total)	0.08	0.12	0.13	0.59	0.16	−0.31	−0.34	−0.23	−0.01	0.07	−0.35	−0.62	−0.66	−0.58	−0.45
Beer	0.00	0.09	0.11	0.51	0.13	−0.31	−0.31	−0.17	−0.04	0.21	−0.26	−0.56	−0.57	−0.54	−0.34
Distilled Beverages	0.12	0.30	0.34	0.40	0.15	0.06	0.16	0.24	0.22	0.19	−0.16	−0.33	−0.28	−0.40	−0.14
Fermented Beverages	0.11	−0.06	−0.30	0.11	−0.45	−0.02	−0.11	−0.21	−0.08	−0.26	−0.18	−0.07	−0.13	−0.08	0.00
Wine	0.06	0.11	0.12	0.41	0.25	−0.28	−0.30	−0.30	0.07	−0.26	−0.24	−0.46	−0.50	−0.33	−0.36
Coffee	−0.10	−0.03	−0.02	0.24	0.26	−0.40	−0.45	−0.41	−0.14	−0.16	−0.23	−0.45	−0.48	−0.19	−0.40
Ref. Sugar and Sweeteners (Total)	−0.22	−0.15	−0.27	0.02	−0.26	−0.26	−0.32	−0.24	−0.21	−0.05	0.13	−0.21	−0.18	−0.06	−0.20
Refined Sugar	−0.18	−0.09	−0.14	−0.05	−0.04	−0.21	−0.22	−0.11	−0.19	0.01	0.18	−0.11	−0.08	0.02	−0.21
Vegetables (Total)	−0.14	−0.09	−0.17	0.09	−0.31	0.10	0.13	−0.03	0.37	−0.09	0.27	0.18	0.16	0.30	0.24
Tomatoes	−0.09	−0.09	−0.14	−0.05	−0.11	0.13	0.15	0.00	0.30	0.03	0.35	0.27	0.19	0.42	0.17
Legumes (Excluding Soybeans)	−0.06	−0.22	−0.16	−0.37	−0.21	−0.19	−0.13	0.03	−0.39	0.00	−0.05	0.22	0.29	0.07	0.41
Legumes (Including Soybeans)	−0.12	−0.31	−0.26	−0.42	−0.44	−0.22	−0.18	−0.04	−0.42	−0.15	−0.06	0.19	0.27	0.04	0.39
Cereals (Total)	0.06	0.16	0.20	0.05	0.10	0.42	0.55	0.45	0.48	0.21	0.35	0.47	0.35	0.41	0.30
Maize	0.02	0.02	−0.02	−0.07	−0.14	−0.08	0.06	0.01	−0.12	−0.29	−0.14	0.04	0.08	−0.10	−0.03
Rice	−0.31	−0.41	−0.36	−0.61	−0.31	−0.05	−0.17	−0.05	−0.43	−0.08	−0.04	0.28	0.45	0.11	0.50
Wheat	0.07	0.22	0.29	0.24	0.27	0.37	0.43	0.36	0.55	0.27	0.43	0.24	0.00	0.34	−0.04
Starchy Roots (Total)	0.20	0.34	0.28	0.50	0.06	0.04	0.20	0.12	0.33	0.06	−0.22	−0.40	−0.50	−0.42	−0.37
Potatoes	0.16	0.36	0.32	0.61	0.10	0.15	0.28	0.13	0.60	0.08	−0.12	−0.37	−0.45	−0.28	−0.36
Oilcrops	−0.28	−0.45	−0.49	−0.45	−0.52	0.01	−0.27	−0.19	−0.36	−0.12	0.17	0.13	0.20	0.15	0.10
Plant Oils	−0.14	−0.28	−0.32	−0.04	−0.32	−0.29	−0.32	−0.33	−0.01	−0.16	0.05	−0.15	−0.22	0.18	−0.10
Sunflower Oil	0.29	0.47	0.43	0.56	0.25	0.24	0.46	0.40	0.56	−0.03	−0.01	−0.17	−0.22	−0.26	−0.11
Tree Nuts	−0.24	−0.22	−0.30	−0.11	−0.17	−0.20	−0.21	−0.22	0.05	−0.17	0.02	−0.07	−0.05	0.27	−0.32
Plant Protein	0.02	0.02	0.01	0.03	−0.19	0.25	0.36	0.25	0.38	−0.01	0.34	0.40	0.29	0.41	0.28
Plant fat	−0.16	−0.31	−0.36	−0.12	−0.29	−0.32	−0.35	−0.38	−0.06	−0.18	0.11	−0.07	−0.13	0.33	−0.05
% CC Energy	0.00	0.03	0.08	−0.28	−0.03	0.35	0.49	0.45	0.22	0.18	0.17	0.57	0.56	0.43	0.49
% SRC Energy	0.18	0.23	0.34	0.23	0.17	0.01	0.14	0.22	0.07	0.17	−0.20	−0.27	−0.45	−0.40	−0.36
% CC Energy and % SRC Energy	0.11	0.10	0.15	−0.20	−0.01	0.34	0.53	0.51	0.25	0.23	0.03	0.48	0.49	0.27	0.47
% CA Energy	0.04	0.07	0.10	−0.33	−0.05	0.26	0.43	0.48	−0.04	0.30	0.04	0.47	0.49	0.21	0.42
% Alcoholic Beverages Energy	0.15	0.28	0.31	0.60	0.29	−0.21	−0.18	−0.06	0.10	0.17	−0.36	−0.62	−0.62	−0.60	−0.41
% Plant Food Energy	−0.03	−0.16	−0.17	−0.53	−0.33	0.18	0.30	0.30	−0.12	0.09	0.15	0.61	0.60	0.51	0.45
Fish & Seafood	−0.27	−0.25	−0.27	−0.19	−0.19	−0.26	−0.30	−0.25	−0.14	−0.44	0.01	−0.08	−0.04	0.08	−0.05
Pelagic Marine Fish	−0.12	−0.12	−0.13	−0.13	−0.08	−0.11	−0.10	−0.04	−0.09	−0.28	0.11	0.02	0.07	0.06	−0.05
Freshwater Fish	−0.13	−0.16	−0.12	−0.09	−0.07	−0.15	−0.15	−0.21	0.03	−0.13	−0.28	−0.23	−0.37	−0.11	−0.18
Meat (Total)	−0.12	−0.10	−0.15	0.25	−0.09	−0.30	−0.46	−0.44	−0.12	−0.16	−0.03	−0.35	−0.31	−0.14	−0.04
Beef	−0.16	−0.20	−0.31	0.04	−0.41	−0.20	−0.33	−0.36	−0.12	−0.38	−0.12	−0.39	−0.37	−0.28	−0.30
Pork	0.03	0.15	0.21	0.55	0.28	−0.26	−0.24	−0.18	0.20	0.16	−0.33	−0.60	−0.63	−0.52	−0.45
Poultry	−0.28	−0.24	−0.25	−0.19	−0.20	−0.30	−0.35	−0.15	−0.35	−0.08	0.30	0.25	0.47	0.34	0.46
Mutton and Goat Meat	0.06	−0.10	−0.13	−0.03	−0.06	0.17	−0.09	−0.12	0.10	−0.08	0.32	0.33	0.38	0.60	0.42
Meat Fat	−0.10	−0.04	−0.04	0.22	0.04	−0.21	−0.37	−0.38	−0.04	−0.24	−0.07	−0.38	−0.34	−0.23	−0.13
Meat Protein	−0.12	−0.11	−0.18	0.21	−0.13	−0.29	−0.46	−0.44	−0.13	−0.27	−0.01	−0.31	−0.26	−0.09	−0.04
Dairy (Total, Excluding Butter)	0.05	0.15	0.08	0.61	0.16	−0.12	−0.17	−0.29	0.38	−0.08	−0.11	−0.49	−0.55	−0.32	−0.39
Cheese	−0.08	−0.04	−0.03	0.42	0.18	−0.35	−0.39	−0.38	0.09	−0.03	−0.19	−0.45	−0.52	−0.05	−0.50
Milk	0.15	0.25	0.17	0.42	−0.12	0.21	0.24	0.09	0.37	−0.18	0.04	−0.24	−0.26	−0.32	−0.07
Dairy Fat	0.07	0.13	0.07	0.53	0.09	−0.09	−0.14	−0.25	0.36	−0.13	−0.08	−0.44	−0.50	−0.27	−0.34
Dairy Protein	0.08	0.19	0.14	0.60	0.26	−0.10	−0.15	−0.27	0.38	−0.07	−0.08	−0.46	−0.53	−0.29	−0.36
Eggs (Total)	−0.11	0.03	0.09	0.21	0.15	−0.19	−0.14	−0.10	0.17	0.15	−0.02	−0.41	−0.35	−0.35	−0.04
Lard	0.05	0.15	0.16	0.39	0.20	−0.19	−0.17	−0.10	0.06	0.19	−0.23	−0.47	−0.42	−0.36	−0.38
Offal	0.16	0.20	0.18	0.43	0.25	−0.05	−0.12	−0.12	0.24	−0.22	−0.06	−0.27	−0.16	−0.33	0.05
Animal Fat	−0.03	0.07	0.06	0.52	0.20	−0.21	−0.32	−0.36	0.20	−0.15	−0.12	−0.53	−0.56	−0.36	−0.44
Animal Protein	−0.10	−0.05	−0.13	0.35	0.04	−0.27	−0.42	−0.49	0.06	−0.45	−0.04	−0.44	−0.45	−0.21	−0.29
Animal Fat and Animal Protein	−0.06	0.02	−0.02	0.47	0.16	−0.24	−0.37	−0.44	0.15	−0.29	−0.09	−0.51	−0.55	−0.31	−0.43
Total Fat	−0.09	−0.10	−0.15	0.36	−0.04	−0.30	−0.41	−0.48	0.13	−0.27	−0.04	−0.43	−0.49	−0.09	−0.42
Total Protein	−0.08	−0.04	−0.13	0.35	−0.07	−0.15	−0.23	−0.38	0.36	−0.45	0.10	−0.22	−0.32	0.14	−0.13
Total Fat and Animal Protein	−0.10	−0.09	−0.15	0.40	−0.02	−0.30	−0.43	−0.51	0.12	−0.36	−0.04	−0.45	−0.50	−0.15	−0.41
Total Fat and Total Protein	−0.09	−0.08	−0.15	0.40	−0.06	−0.26	−0.37	−0.47	0.25	−0.37	0.01	−0.38	−0.46	0.00	−0.36
Total Energy	−0.13	−0.07	−0.15	0.26	−0.14	−0.17	−0.19	−0.32	0.32	−0.23	0.14	−0.18	−0.31	0.21	−0.20
Raised Cholesterol (Men)	−0.12	0.05	0.05	0.42	0.32	−0.24	−0.34	−0.39	0.18	−0.24	0.07	−0.35	−0.41	−0.05	−0.23
Raised Blood Pressure (Men)						0.42	0.60	0.65	0.60	0.57	0.04	0.08	0.10	−0.16	0.21
CVD Mortality (Men)	0.42	0.60	0.65	0.60	0.57						0.30	0.32	0.31	0.10	0.35
Raised Blood Glucose (Men)	0.04	0.08	0.10	−0.16	0.21	0.30	0.32	0.31	0.10	0.35					
Mean BMI (Men)	−0.10	0.06	0.11	0.14	0.20	−0.02	0.00	−0.04	0.19	0.37	0.40	0.22	0.32	0.36	0.44
Obesity (Men)	−0.09	0.09	0.15	0.21	0.23	−0.08	−0.11	0.00	0.10	0.26	0.39	0.18	0.31	0.40	0.31
Life Expectancy (Men)	−0.39	−0.40	−0.49	−0.29	−0.43	−0.33	−0.58	−0.75	−0.29	−0.84	0.08	−0.31	−0.37	0.01	−0.55
Health Expenditure (2012)	−0.27	−0.30	−0.38	0.17	−0.30	−0.43	−0.51	−0.53	−0.10	−0.33	−0.14	−0.39	−0.43	0.17	−0.35
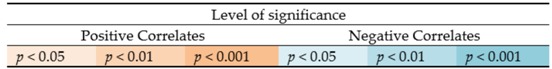															

**Table 5:** Pearson linear correlations in women: differences according to health expenditure per capita (constant 2011 international USD, 2012).

Variable	Raised Blood Pressure (Women)					CVD Mortality (Women)					Raised Blood Glucose (Women)				
Category	Total Sample	>500 USD	1000 USD	500–2000 USD	2000 USD	Total Sample	>500 USD	1000 USD	500–2000 USD	2000 USD	Total Sample	>500 USD	1000 USD	500–2000 USD	2000 USD
*n* (countries)	158	92	60	61	31	158	92	60	61	31	158	92	60	61	31
Fruits (Total)	−0.53	−0.41	−0.26	−0.38	−0.09	−0.46	−0.49	−0.31	−0.44	−0.29	−0.08	−0.15	−0.01	0.09	−0.25
Apples	−0.52	−0.30	−0.25	0.18	−0.04	−0.37	−0.26	−0.27	0.23	−0.25	−0.25	−0.47	−0.49	−0.16	−0.37
Grapes	−0.24	0.00	−0.05	0.21	0.14	0.02	0.08	−0.07	0.33	0.00	0.06	−0.07	−0.14	0.08	0.03
Oranges and Mandarins	−0.60	−0.49	−0.42	−0.33	−0.20	−0.53	−0.52	−0.44	−0.38	−0.27	−0.05	−0.22	−0.15	0.17	−0.19
Alcoholic Beverages (Total)	−0.41	−0.37	−0.31	0.13	0.00	−0.44	−0.46	−0.38	−0.14	−0.10	−0.53	−0.72	−0.73	−0.64	−0.46
Beer	−0.55	−0.37	−0.26	0.04	0.01	−0.50	−0.43	−0.30	−0.21	0.05	−0.44	−0.65	−0.63	−0.63	−0.32
Distilled Beverages	−0.32	−0.02	0.09	−0.01	0.04	−0.20	−0.04	0.06	−0.04	0.06	−0.22	−0.33	−0.26	−0.43	−0.22
Fermented Beverages	0.25	0.08	−0.34	0.34	−0.42	0.11	0.04	−0.25	0.15	−0.28	−0.14	0.01	−0.19	0.07	−0.04
Wine	−0.43	−0.31	−0.22	0.01	0.12	−0.41	−0.39	−0.37	−0.05	−0.30	−0.44	−0.57	−0.57	−0.43	−0.41
Coffee	−0.60	−0.50	−0.43	−0.13	−0.04	−0.55	−0.54	−0.48	−0.26	−0.24	−0.44	−0.61	−0.60	−0.35	−0.46
Ref. Sugar and Sweeteners (Total)	−0.65	−0.43	−0.37	−0.27	−0.39	−0.53	−0.45	−0.31	−0.41	−0.04	0.02	−0.26	−0.14	−0.07	−0.18
Refined Sugar	−0.57	−0.27	−0.14	−0.25	−0.13	−0.47	−0.34	−0.16	−0.38	0.04	0.10	−0.12	0.01	0.02	−0.15
Vegetables (Total)	−0.37	−0.06	−0.08	0.17	−0.10	−0.07	0.12	0.05	0.37	0.05	0.11	0.07	0.08	0.20	0.31
Tomatoes	−0.22	0.06	0.02	0.21	0.07	0.00	0.17	0.10	0.34	0.14	0.25	0.24	0.15	0.41	0.24
Legumes (Excluding Soybeans)	0.28	0.11	0.16	−0.14	0.08	−0.02	−0.01	0.14	−0.28	0.12	0.07	0.30	0.36	0.12	0.49
Legumes (Including Soybeans)	0.22	0.02	0.07	−0.19	−0.20	−0.06	−0.06	0.07	−0.30	−0.06	0.05	0.26	0.33	0.10	0.40
Cereals (Total)	0.26	0.48	0.47	0.37	0.26	0.41	0.59	0.53	0.52	0.26	0.40	0.52	0.41	0.42	0.36
Maize	0.27	0.27	0.18	0.14	−0.06	0.06	0.17	0.07	0.01	−0.29	0.03	0.19	0.19	0.03	0.01
Rice	0.02	−0.12	−0.02	−0.48	−0.16	0.05	−0.07	0.09	−0.36	0.06	0.05	0.31	0.48	0.14	0.46
Wheat	−0.19	0.24	0.26	0.35	0.34	0.16	0.38	0.35	0.52	0.26	0.31	0.18	−0.02	0.27	0.05
Starchy Roots (Total)	0.21	0.07	−0.03	0.23	−0.02	0.10	0.05	−0.09	0.15	−0.08	−0.21	−0.40	−0.50	−0.46	−0.33
Potatoes	−0.34	−0.06	−0.02	0.22	0.02	−0.16	0.01	−0.07	0.28	−0.05	−0.25	−0.43	−0.50	−0.36	−0.31
Oilcrops	−0.08	−0.25	−0.23	−0.33	−0.35	−0.04	−0.19	−0.11	−0.27	−0.05	0.23	0.12	0.22	0.16	0.15
Plant Oils	−0.42	−0.41	−0.38	−0.03	−0.19	−0.39	−0.39	−0.33	−0.11	−0.15	−0.17	−0.29	−0.31	0.06	−0.04
Sunflower Oil	−0.11	0.31	0.31	0.35	0.28	0.05	0.39	0.36	0.50	−0.05	−0.10	−0.17	−0.20	−0.31	−0.13
Tree Nuts	−0.40	−0.28	−0.24	0.02	−0.12	−0.28	−0.21	−0.15	0.08	−0.13	−0.12	−0.17	−0.11	0.18	−0.24
Plant Protein	0.20	0.31	0.24	0.36	0.02	0.25	0.40	0.34	0.45	0.07	0.33	0.39	0.28	0.40	0.35
Plant fat	−0.38	−0.40	−0.39	−0.01	−0.15	−0.39	−0.40	−0.34	−0.10	−0.13	−0.10	−0.22	−0.23	0.22	0.01
% CC Energy	0.50	0.56	0.51	0.25	0.16	0.51	0.63	0.57	0.42	0.24	0.34	0.70	0.66	0.54	0.50
% SRC Energy	0.35	0.19	0.11	0.13	0.07	0.16	0.12	0.02	0.04	0.00	−0.15	−0.20	−0.41	−0.38	−0.33
% CC Energy and % SRC Energy	0.69	0.61	0.55	0.32	0.19	0.58	0.66	0.60	0.45	0.26	0.22	0.64	0.60	0.39	0.48
% CA Energy	0.62	0.59	0.53	0.19	0.10	0.47	0.57	0.56	0.16	0.34	0.30	0.67	0.65	0.41	0.43
% Alcoholic Beverages Energy	−0.40	−0.23	−0.15	0.12	0.11	−0.39	−0.33	−0.23	−0.07	−0.03	−0.51	−0.69	−0.68	−0.64	−0.45
% Plant Food Energy	0.64	0.48	0.37	0.14	−0.04	0.45	0.47	0.44	0.12	0.21	0.36	0.75	0.71	0.64	0.50
Fish and Seafood	−0.43	−0.38	−0.32	−0.23	−0.34	−0.35	−0.34	−0.26	−0.17	−0.44	−0.12	−0.19	−0.10	0.02	−0.19
Pelagic Marine Fish	−0.20	−0.13	−0.07	−0.14	−0.25	−0.18	−0.13	−0.03	−0.13	−0.29	0.04	−0.02	0.07	0.03	−0.15
Freshwater Fish	−0.07	−0.30	−0.38	−0.12	−0.22	−0.10	−0.19	−0.31	0.03	−0.26	−0.28	−0.29	−0.44	−0.13	−0.24
Meat (Total)	−0.70	−0.58	−0.48	−0.31	−0.02	−0.53	−0.59	−0.50	−0.34	−0.09	−0.26	−0.54	−0.43	−0.31	0.01
Beef	−0.54	−0.46	−0.46	−0.27	−0.40	−0.38	−0.44	−0.46	−0.27	−0.41	−0.26	−0.43	−0.37	−0.30	−0.24
Pork	−0.56	−0.40	−0.26	−0.03	0.13	−0.48	−0.40	−0.29	−0.02	0.01	−0.54	−0.73	−0.71	−0.65	−0.47
Poultry	−0.59	−0.33	−0.09	−0.41	0.05	−0.48	−0.38	−0.08	−0.45	0.09	0.15	0.12	0.44	0.23	0.55
Mutton & Goat Meat	0.00	−0.05	−0.03	0.23	0.05	0.14	−0.02	0.00	0.23	0.12	0.23	0.21	0.28	0.55	0.39
Meat Fat	−0.65	−0.53	−0.39	−0.31	−0.04	−0.46	−0.51	−0.46	−0.23	−0.23	−0.28	−0.56	−0.45	−0.37	−0.18
Meat Protein	−0.67	−0.56	−0.47	−0.29	−0.06	−0.51	−0.58	−0.49	−0.33	−0.17	−0.24	−0.49	−0.36	−0.25	0.02
Dairy (Total, Excluding Butter)	−0.54	−0.36	−0.34	0.14	0.02	−0.37	−0.33	−0.40	0.17	−0.15	−0.31	−0.62	−0.64	−0.45	−0.36
Cheese	−0.59	−0.50	−0.43	0.00	0.00	−0.53	−0.51	−0.48	−0.13	−0.09	−0.40	−0.58	−0.61	−0.18	−0.43
Milk	−0.26	0.04	0.02	0.13	−0.14	−0.02	0.15	0.03	0.27	−0.19	−0.05	−0.26	−0.28	−0.38	−0.10
Dairy Fat	−0.49	−0.33	−0.33	0.12	−0.06	−0.32	−0.27	−0.35	0.20	−0.17	−0.26	−0.56	−0.60	−0.38	−0.34
Dairy Protein	−0.52	−0.32	−0.31	0.14	0.06	−0.35	−0.30	−0.37	0.18	−0.11	−0.28	−0.59	−0.62	−0.41	−0.34
Eggs Total	−0.66	−0.43	−0.26	−0.26	0.03	−0.47	−0.33	−0.18	−0.08	0.09	−0.25	−0.55	−0.46	−0.49	−0.04
Lard	−0.44	−0.28	−0.16	−0.08	0.12	−0.39	−0.31	−0.21	−0.13	0.07	−0.36	−0.54	−0.46	−0.46	−0.33
Offal	−0.30	−0.15	−0.06	0.07	0.17	−0.22	−0.19	−0.15	0.12	−0.14	−0.21	−0.36	−0.23	−0.40	0.03
Animal Fat	−0.67	−0.52	−0.43	−0.14	−0.04	−0.48	−0.49	−0.48	−0.06	−0.23	−0.35	−0.70	−0.68	−0.53	−0.46
Animal Protein	−0.71	−0.61	−0.57	−0.24	−0.17	−0.53	−0.58	−0.58	−0.20	−0.43	−0.29	−0.64	−0.60	−0.40	−0.33
Animal Fat & Animal Protein	−0.70	−0.58	−0.51	−0.19	−0.09	−0.51	−0.55	−0.55	−0.13	−0.33	−0.33	−0.70	−0.68	−0.50	−0.46
Total Fat	−0.66	−0.59	−0.53	−0.12	−0.15	−0.53	−0.56	−0.54	−0.12	−0.29	−0.30	−0.63	−0.63	−0.31	−0.39
Total Protein	−0.61	−0.44	−0.46	0.07	−0.15	−0.41	−0.37	−0.43	0.17	−0.38	−0.14	−0.44	−0.48	−0.05	−0.13
Total Fat & Animal Protein	−0.70	−0.62	−0.58	−0.18	−0.17	−0.54	−0.59	−0.59	−0.16	−0.37	−0.31	−0.66	−0.66	−0.38	−0.41
Total Fat & Total Protein	−0.66	−0.57	−0.54	−0.05	−0.17	−0.50	−0.52	−0.54	0.00	−0.36	−0.25	−0.59	−0.62	−0.23	−0.34
Total Energy	−0.61	−0.41	−0.40	0.07	−0.16	−0.43	−0.35	−0.36	0.11	−0.19	−0.09	−0.36	−0.43	0.05	−0.15
Raised Cholesterol (Women)	−0.73	−0.47	−0.35	−0.11	0.07	−0.52	−0.48	−0.35	−0.10	−0.23	−0.09	−0.48	−0.47	−0.14	−0.21
Raised Blood Press. (Women)						0.69	0.82	0.83	0.71	0.73	0.28	0.60	0.57	0.27	0.52
CVD Mortality (Women)	0.69	0.82	0.83	0.71	0.73						0.36	0.54	0.55	0.21	0.65
Raised Blood Glucose (Women)	0.28	0.60	0.57	0.27	0.52	0.36	0.54	0.55	0.21	0.65					
Mean BMI (Women)	−0.29	0.28	0.30	0.01	0.32	−0.13	0.24	0.28	−0.04	0.48	0.62	0.76	0.77	0.80	0.67
Obesity (Women)	−0.28	0.33	0.47	0.13	0.41	−0.17	0.16	0.37	−0.12	0.50	0.57	0.65	0.76	0.64	0.65
Life Expectancy (Women)	−0.79	−0.69	−0.68	−0.52	−0.54	−0.54	−0.60	−0.70	−0.34	−0.82	−0.12	−0.58	−0.67	−0.33	−0.78
Health Expenditure (2012)	−0.71	−0.67	−0.68	−0.18	−0.46	−0.59	−0.60	−0.58	−0.30	−0.38	−0.38	−0.56	−0.56	0.00	−0.38
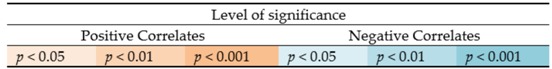															

**Table 6:** Partial correlations of raised blood pressure and CVD mortality with nutritional factors, adjusted for smoking and health expenditure per capita.

Variable	Raised Blood Pressure (Men)		CVD Mortality (Men)		Raised Blood Pressure (Women)		CVD Mortality (Women)	
Category	Total Sample	Health Exp. > 500 USD	Total Sample	Health Exp. > 500 USD	Total Sample	Health Exp. > 500 USD	Total Sample	Health Exp. > 500 USD
Adjustment	Health Expenditure per Capita, Current Smoking of any Tobacco Product				Health Expenditure per Capita			
*n* (countries)	115	74	115	74	158	92	158	92
Fruits (Total)	−0.25	−0.31	−0.19	−0.35	−0.35	−0.28	−0.28	−0.39
Apples	0.35	0.38	0.26	0.22	0.00	0.18	0.12	0.17
Grapes	0.02	−0.04	0.10	−0.06	−0.05	0.09	0.25	0.18
Oranges and Mandarins	−0.21	−0.22	−0.23	−0.28	−0.32	−0.23	−0.28	−0.30
Alcoholic Beverages (Total)	0.39	0.48	0.04	0.03	0.08	0.07	−0.10	−0.14
Beer	0.36	0.42	0.07	0.08	−0.08	0.03	−0.15	−0.14
Distilled Beverages	0.31	0.37	0.24	0.18	−0.15	0.05	−0.02	0.02
Fermented Beverages	0.05	−0.10	−0.02	−0.11	0.22	0.09	0.03	0.04
Wine	0.32	0.33	−0.06	−0.11	0.05	0.07	−0.06	−0.11
Coffee	0.29	0.40	−0.07	−0.09	−0.07	0.02	−0.15	−0.16
Ref. Sugar and Sweeteners (Total)	0.02	0.05	0.02	−0.03	−0.38	−0.13	−0.25	−0.20
Refined Sugar	0.03	0.04	0.01	−0.07	−0.36	−0.12	−0.25	−0.22
Vegetables (Total)	−0.16	−0.33	0.06	−0.18	−0.20	−0.03	0.16	0.18
Tomatoes	−0.09	−0.27	0.13	−0.07	−0.07	0.08	0.19	0.20
Legumes (Excluding Soybeans)	−0.13	−0.33	−0.29	−0.35	0.10	−0.09	−0.26	−0.22
Legumes (Including Soybeans)	−0.22	−0.46	−0.36	−0.45	0.03	−0.20	−0.29	−0.28
Cereals (Total)	−0.06	−0.10	0.14	0.25	0.12	0.32	0.33	0.47
Maize	−0.01	−0.03	−0.10	0.01	0.12	0.11	−0.13	0.00
Rice	−0.54	−0.59	−0.39	−0.39	−0.29	−0.43	−0.17	−0.31
Wheat	0.20	0.15	0.43	0.31	−0.01	0.31	0.39	0.46
Starchy Roots (Total)	0.26	0.41	0.18	0.34	0.20	0.18	0.05	0.12
Potatoes	0.44	0.51	0.44	0.47	−0.01	0.18	0.16	0.24
Oilcrops	−0.36	−0.59	−0.09	−0.50	−0.24	−0.41	−0.15	−0.30
Plant Oils	−0.07	−0.24	−0.15	−0.23	−0.02	−0.11	−0.08	−0.14
Sunflower Oil	0.40	0.41	0.34	0.37	0.05	0.31	0.20	0.40
Tree Nuts	−0.13	−0.20	−0.15	−0.23	−0.14	−0.07	−0.03	0.00
Plant Protein	−0.08	−0.23	0.06	0.05	0.16	0.23	0.23	0.35
Plant fat	−0.12	−0.29	−0.19	−0.30	−0.01	−0.12	−0.11	−0.16
% CC Energy	−0.33	−0.35	−0.12	0.08	0.12	0.20	0.23	0.38
% SRC Energy	0.16	0.28	0.06	0.21	0.24	0.11	0.01	0.03
% CC Energy and % SRC Energy	−0.22	−0.28	−0.08	0.16	0.34	0.25	0.27	0.42
% CA Energy	−0.30	−0.26	−0.12	0.08	0.16	0.19	0.04	0.23
% Alcoholic Beverages Energy	0.47	0.56	0.13	0.09	0.01	0.11	−0.08	−0.08
% Plant Food Energy	−0.46	−0.61	−0.29	−0.17	0.23	0.06	0.03	0.12
Fish and Seafood	−0.21	−0.18	−0.27	−0.30	−0.29	−0.30	−0.20	−0.24
Pelagic Marine Fish	−0.02	0.00	−0.05	−0.12	−0.18	−0.18	−0.14	−0.16
Freshwater Fish	−0.23	−0.18	−0.25	−0.05	−0.04	−0.13	−0.09	−0.01
Meat (Total)	0.18	0.18	0.10	−0.08	−0.32	−0.21	−0.15	−0.30
Beef	−0.02	−0.07	0.16	0.03	−0.19	−0.20	−0.04	−0.20
Pork	0.40	0.47	0.03	0.01	−0.11	−0.01	−0.11	−0.06
Poultry	−0.18	−0.23	−0.12	−0.20	−0.40	−0.23	−0.26	−0.30
Mutton and Goat Meat	0.05	−0.12	0.20	0.00	0.08	0.02	0.23	0.05
Meat Fat	0.15	0.18	0.12	−0.06	−0.29	−0.18	−0.06	−0.20
Meat Protein	0.16	0.12	0.10	−0.09	−0.29	−0.21	−0.13	−0.30
Dairy (Total, Excluding Butter)	0.46	0.50	0.31	0.25	−0.01	0.17	0.14	0.13
Cheese	0.28	0.27	−0.07	−0.12	−0.04	−0.01	−0.10	−0.13
Milk	0.31	0.29	0.43	0.39	−0.04	0.15	0.23	0.27
Dairy Fat	0.41	0.42	0.30	0.25	−0.02	0.12	0.14	0.15
Dairy Protein	0.47	0.52	0.30	0.25	−0.01	0.18	0.14	0.14
Eggs (Total)	0.16	0.15	0.08	0.00	−0.36	−0.19	−0.13	−0.07
Lard	0.33	0.38	0.12	0.11	−0.10	0.01	−0.10	−0.08
Offal	0.31	0.30	0.21	0.08	−0.06	−0.01	0.01	−0.07
Animal Fat	0.41	0.51	0.25	0.15	−0.21	−0.04	−0.01	−0.07
Animal Protein	0.30	0.33	0.14	−0.03	−0.33	−0.21	−0.11	−0.25
Animal Fat and Animal Protein	0.38	0.47	0.21	0.08	−0.28	−0.12	−0.06	−0.16
Total Fat	0.25	0.19	0.06	−0.11	−0.17	−0.12	−0.09	−0.17
Total Protein	0.22	0.14	0.16	0.00	−0.17	−0.03	0.06	0.03
Total Fat and Animal Protein	0.28	0.26	0.10	−0.08	−0.25	−0.17	−0.10	−0.22
Total Fat and Total Protein	0.25	0.19	0.11	−0.07	−0.19	−0.09	−0.10	−0.10
Total Energy	0.11	0.03	0.10	−0.02	−0.18	0.03	0.01	0.06
Raised Cholesterol	0.18	0.38	0.08	−0.03	−0.43	−0.06	−0.17	−0.14
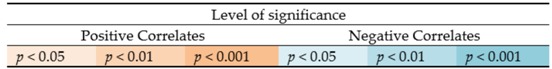								

**Table 7:** Relationship between men’s raised blood pressure, CVD mortality and the examined variables, including smoking prevalence (115 countries).

Raised Blood Pressure (Men)			CVD Mortality (Men)		
**Positive Correlates**	**Mean**	**Correlation (*p*-Values)**	**Positive Correlates**	**Mean**	**Correlation (*p*-Values)**
CVD Mortality	301.3	0.50 (*p* < 0.001)	Current smoking of any tobacco product (%)	34.1	0.53 (*p* < 0.001)
Sunflower oil	5.9	0.34 (*p* < 0.001)	Daily smoking of any tobacco product (%)	27.3	0.52 (*p* < 0.001)
Potatoes	116.3	0.26 (*p* = 0.005)	Current smoking of cigarettes (%)	28.3	0.52 (*p* < 0.001)
Starchy roots	199.5	0.25 (*p* = 0.008)	Raised blood pressure (%)	28.1	0.50 (*p* < 0.001)
Distilled beverages	8.7	0.22 (*p* = 0.020)	Daily smoking of cigarettes (%)	23.1	0.49 (*p* < 0.001)
% Alcoholic beverages energy	3.0	0.21 (*p* = 0.024)	Cereals (total)	378.1	0.42 (*p* < 0.001)
Milk	185.7	0.21 (*p* = 0.026)	Wheat	201.9	0.33 (*p* < 0.001)
% SRC energy	5.6	0.20 (*p* = 0.036)	Raised blood glucose (%)	8.7	0.33 (*p* < 0.001)
**Daily smoking of any tobacco product (%)**	27.3	0.18 (*p* = 0.054)	% CC energy and % SRC energy	41.3	0.33 (*p* < 0.001)
Offals	8.4	0.17 (*p* = 0.07)	% CC energy	35.7	0.32 (*p* < 0.001)
**Negative Correlates**	**Mean**	**Correlation (*p*-Values)**	**Negative Correlates**	**Mean**	**Correlation (*p*-Values)**
Rice	78.6	−0.37 (*p* < 0.001)	Oranges and Mandarins	41.0	−0.48 (*p* < 0.001)
Fruits total	208.2	−0.36 (*p* < 0.001)	Health Expenditure	1472.3	−0.47 (*p* < 0.001)
Life Expectancy	69.6	−0.36 (*p* < 0.001)	Fruits (total)	208.2	−0.44 (*p* < 0.001)
Oranges and Mandarins	41.0	−0.35 (*p* < 0.001)	Coffee	6.3	−0.41 (*p* < 0.001)
Fish and Seafood	44.8	−0.30 (*p* < 0.001)	Plant Fat	43.5	−0.39 (*p* < 0.001)
Poultry	39.7	−0.30 (*p* = 0.001)	Life Expectancy	69.6	−0.39 (*p* < 0.001)
Oilcrops	15.3	−0.30 (*p* = 0.001)	Cheese	13.7	−0.38 (*p* < 0.001)
Health Expenditure	1472.3	−0.30 (*p* = 0.001)	Fish and Seafood	44.8	−0.38 (*p* < 0.001)
Plant fat	43.5	−0.26 (*p* = 0.005)	Plant Oils	30.5	−0.37 (*p* < 0.001)
Plant oils	30.5	−0.23 (*p* = 0.013)	Poultry	39.7	−0.35 (*p* < 0.001)
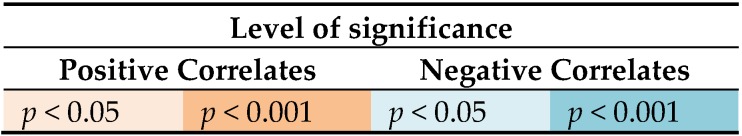					

Abbreviations: % CC energy = the mean proportion of carbohydrate energy from cereals; % SRC energy = the mean proportion of carbohydrate energy from starchy roots.

**Table 8:** Frequencies of significant correlates of CVD indicators (*p* < 0.05) across five pre-defined regions in both sexes (see Table 2 and Table 3).

Positive Correlates					
Frequency	Raised Blood Pressure	Frequency	CVD Mortality	Frequency	Raised Blood Glucose
8	CVD mortality	8	Raised blood pressure	9	Mean BMI; Obesity
6	% CA energy; % CC energy;	6	Cereals (total);	8	Refined sugar; Wheat
	Raised blood glucose		% CC energy and % SRC energy;	6	Plant protein; Poultry;
5	% CC energy & % SRC energy;		Wheat		Raised blood pressure;
	% Plant food energy	5	% CC energy;		Ref. sugar and Sweeteners (total);
4	% SRC energy		Raised blood glucose		Total energy
3	Mutton and Goat meat; Wheat	4	Plant protein; % CA energy; Milk	5	Cereals (total); CVD mortality; Tomatoes; Total fat and Total protein; Total protein; Sunflower oil
				
				
**Negative Correlates**					
**Frequency**	**Raised Blood Pressure**	**Frequency**	**CVD Mortality**	**Frequency**	**Raised Blood Glucose**
9	Health expenditure	9	Life expectancy	7	Freshwater fish
8	Life expectancy	7	Alcoholic beverages (total); Beer; Cheese;	6	Alcoholic beverages (total);
7	Beer; Fruits (total);		Eggs (total); Raised cholesterol		% Alcoholic beverages energy; Pork
	Raised cholesterol	6	Coffee; Fruits (total); Health expenditure; Oranges and Mandarins; Pork; Poultry; Ref. sugar and Sweeteners total	5	Starchy roots (total)
6	Animal fat; Animal protein; Animal fat and Animal protein; Apples; Beef meat; Cheese; Fish and seafood; Meat (total); Meat protein; Pork; Total fat; Total fat and Animal protein; Total fat and Total protein; Total protein; Total energy; Tree nuts			4	% SRC energy
				3	Beef meat; Beer; % CA energy; Eggs (total); Lard; Legumes (including Soybeans); Plant oils
	
		5	% Alcoholic beverages energy; Animal protein; Fish and Seafood; Total fat; Total fat and Animal protein Total fat and Total protein; Total energy		
	
			
			

Abbreviations: % CC energy = the mean proportion of carbohydrate energy from cereals; % SRC energy = the mean proportion of carbohydrate energy from starchy roots; % CA energy = the mean proportion of energy from carbohydrates and alcohol.

**Table 9:** Relationship between CVD mortality and independent variables in 31 countries with health expenditure above 2000 USD per capita.

CVD Mortality (Men)			CVD Mortality (Women)		
**Positive Correlates**	**Mean**	**Correlation (*p*-Values)**	**Positive Correlates**	**Mean**	**Correlation (*p*-Values)**
Raised blood pressure	25.8	0.57 (*p* < 0.001)	Raised blood pressure	16.9	0.73 (*p* < 0.001)
Raised blood glucose	8.0	0.35 (*p* = 0.056)	Raised blood glucose	6.0	0.65 (*p* < 0.001)
% CA energy	52.0	0.30 (*p* = 0.10)	% CA energy	52.0	0.34 (*p* = 0.06)
Wheat	249.2	0.27 (*p* = 0.14)	Cereals (total)	320.0	0.26 (*p* = 0.16)
% CC energy & % SRC energy	26.1	0.23 (*p* = 0.22)	Wheat	249.2	0.26 (*p* = 0.16)
**Negative Correlates**	**Mean**	**Correlation (*p*-Values)**	**Negative Correlates**	**Mean**	**Correlation (*p*-Values)**
Life expectancy	78.3	−0.84 (*p* < 0.001)	Life expectancy	83.2	−0.82 (*p* < 0.001)
Animal protein	62.2	−0.45 (*p* = 0.011)	Fish and Seafood	77.3	−0.44 (*p* = 0.013)
Total protein	103.7	−0.45 (*p* = 0.011)	Animal protein	62.2	−0.43 (*p* = 0.016)
Fish and Seafood	77.3	−0.44 (*p* = 0.014)	Beef meat	55.7	−0.41 (*p* = 0.022)
Beef meat	55.7	−0.38 (*p* = 0.036)	Health expenditure	3895.4	−0.38 (*p* = 0.036)
Total fat and Total protein	236.8	−0.37 (*p* = 0.043)	Total protein	103.7	−0.38 (*p* = 0.036)
Total fat and Animal protein	195.3	−0.36 (*p* = 0.048)	Total fat and Animal protein	195.3	−0.37 (*p* = 0.042)
Fruits (total)	290.4	−0.34 (*p* = 0.062)	Total fat and Total protein	236.8	−0.36 (*p* = 0.048)
Health expenditure	3895.4	−0.33 (*p* = 0.071)	Animal fat and Animal protein	134.9	−0.33 (*p* = 0.068)
Maize	19.1	−0.29 (*p* = 0.11)	Wine	53.1	−0.30 (*p* = 0.11)
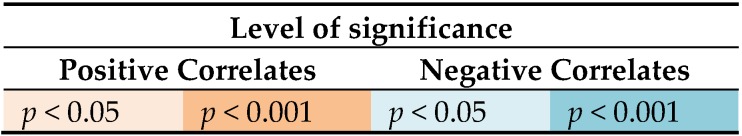					

Abbreviations: % CC energy = the mean proportion of carbohydrate energy from cereals; % SRC energy = the mean proportion of carbohydrate energy from starchy roots; % CA energy = the mean proportion of energy from carbohydrates and alcohol.

**Table 10:** Results of three penalized regression models (ridge, LASSO, elastic net) of women’s CVD indicators. Variables are sorted according to the sign of their *beta* coefficients in the models (positive/negative), and their frequency among the top 10 variables with the highest *beta* coefficients (in parentheses).

Direction of *beta* Coefficients	Total Sample (158 Countries)	Countries with Health Expenditure above 500 USD per Capita (92 Countries)
Women’s Raised Blood Pressure		
Positive	Cereals (total) (2)	Mean BMI; Sunflower oil (3)
Negative	Poultry; Eggs total; Fish and Seafood; Fruits (total); Oranges and Mandarins; Oilcrops (3) Health expenditure; Meat fat; Raised cholesterol (2)	Health expenditure; Fish and Seafood; Oranges and Mandarins; Plant oils (3) Beef; Eggs; Lard (2)
Women’s CVD Mortality		
Positive	% SRC energy; Sunflower oil; Wheat (3)	Sunflower oil (3)
	% CC energy and % SRC energy (2)	Cereals (total) (2)
Negative	Beef; Health Expenditure (3) Refined sugar; Legumes (including Soybeans) (2)	Fish and Seafood; Oranges and Mandarins; Pelagic marine fish; Refined sugar; Wine (3) Plant oils (2)
Women’s Raised Blood Glucose		
Positive	Mean BMI; Pelagic marine fish (3) % CC energy; Obesity (2)	Mean BMI; % CA energy (3)
Negative	Pork (3)	Beer; Coffee; Lard (3)
	Alcoholic beverages (total), % Alcoholic beverages energy; Coffee; Total fat and Animal protein (2)	Animal fat and Animal protein; Beef; Distilled beverages; Eggs; Total protein (2)

Abbreviations: % CC energy = the mean proportion of carbohydrate energy from cereals; % SRC energy = the mean proportion of carbohydrate energy from starchy roots; % CA energy = the mean proportion of energy from carbohydrates and alcohol. Note: For more detailed results, see Supplementary dataset, Sheets S4–S6.

## Supplementary Materials

The following are available online at http://www.mdpi.com/2072-6643/10/4/411/s1. Figure S1: Correlation between the prevalence of men’s raised pressure (%) by WHO (for 2010) and by Nichols et al. (for 2008, including people using blood pressure medications), Figure S2: Correlation between the prevalence of women’s raised pressure by WHO (for 2010) and by Nichols et al. (for 2008, including people using blood pressure medications), Figure S3: Correlation between the prevalence of men’s raised pressure (%) by WHO (for 2010, excluding people using medications) and the mean consumption of total fat & animal protein (g/day per capita; FAOSTAT, 1993–2011), Figure S4: Correlation between the prevalence of men’s raised pressure by Nichols et al. (for 2008, including people using medications) and the mean consumption of total fat and animal protein (g/day per capita; FAOSTAT, 1993–2011), Figure S5: Correlation between the prevalence of women’s raised pressure (%) by WHO (for 2010, excluding people using medications) and the mean consumption of total fat and animal protein (g/day per capita; FAOSTAT, 1993–2011), Figure S6: Correlation between the prevalence of women’s raised pressure by Nichols et al. (for 2008, including people using medications) and the mean consumption of total fat and animal protein (g/day per capita; FAOSTAT, 1993–2011), Figure S7: Correlation between men’s CVD mortality by WHO (2012) and the actual men’s CVD mortality in by Nichols et al. in 42 European countries (per 100,000 population), Figure S8: Correlation between women’s CVD mortality by WHO (2012) and the actual women’s CVD mortality in by Nichols et al. in 42 European countries (per 100,000 population), Figure S9: Correlation between the prevalence of men’s raised glucose (%) by WHO (for 2010) and by Nichols et al. (for 2008) in 42 European countries, Figure S10: Correlation between the prevalence of women’s raised glucose by WHO (for 2010) and by Nichols et al. (for 2008) in 42 European countries, Figure S11: Relationship between life expectancy in men and women (WHO, 2012), Figure S12: Relationship between the prevalence of men’s raised blood pressure (%; WHO, 2010) and the mean proportion of energy from alcoholic beverages (%; FAOSTAT, 1993–2011), Figure S13: Relationship between the prevalence of men’s raised blood pressure (%; WHO, 2010) and the mean consumption of distilled beverages (g/day per capita; (FAOSTAT, 1993–2011), Figure S14: Relationship between the prevalence of women’s raised blood pressure (%; WHO, 2010) and the mean consumption of wheat (g/day per capita; FAOSTAT, 1993–2011), Figure S15: Relationship between the prevalence of women’s raised blood pressure (%; WHO, 2010) and the mean consumption of maize (g/day per capita; FAOSTAT, 1993–2011), Figure S16: Relationship between the prevalence of men’s raised blood pressure (%; WHO, 2010) and men‘s life expectancy (World Bank, 2012), Figure S17: Relationship between the prevalence of men’s raised blood pressure (%; WHO, 2010) and health expenditure per capita for 2012 (in USD), Figure S18: Relationship between men’s CVD mortality (WHO, 2012) and the mean proportion of energy from alcoholic beverages (%; FAOSTAT, 1993–2011), Figure S19: Relationship between men’s CVD mortality (WHO, 2012) and the mean consumption of distilled beverages (g/day per capita; (FAOSTAT, 1993–2011), Figure S20: Relationship between men’s CVD mortality (WHO, 2012) and the prevalence of men’s raised cholesterol (%; WHO, 2010), Figure S21: Relationship between women’s CVD mortality (WHO, 2012) and the prevalence of women’s raised cholesterol (%; WHO, 2010), Figure S22: Relationship between men’s CVD mortality (WHO, 2012) and men’s life expectancy (World Bank, 2012), Figure S23: Relationship between women’s CVD mortality (WHO, 2012) and women’s life expectancy (World Bank, 2012), Figure S24: Relationship between the prevalence of women’s raised blood glucose (%; WHO, 2010) and the mean consumption of pork (g/day per capita; FAOSTAT, 1993–2011), Figure S25: Relationship between the prevalence of women’s raised blood glucose (%; WHO, 2010) and the mean consumption of alcoholic beverages (g/day per capita, FAOSTAT, 1993–2011), Figure S26: Relationship between the prevalence of women’s raised blood glucose (%; WHO, 2010) and the mean proportion of carbohydrate energy from cereals (% CC energy) in the diet (FAOSTAT, 1993–2011), Figure S27: Relationship between the prevalence of women’s raised blood glucose (%; WHO, 2010) and the mean proportion of carbohydrate energy from cereals (% CC energy) in the diet (FAOSTAT, 1993–2011), Figure S28: Relationship between the prevalence of women’s raised blood glucose (%; WHO, 2010) and the mean consumption of animal fat (g/day per capita; FAOSTAT, 1993–2011), Figure S29: Relationship between the prevalence of women’s raised blood glucose (%; WHO, 2010) and the mean consumption of animal fat (g/day per capita; FAOSTAT, 1993–2011), Figure S30: Relationship between the prevalence of men’s raised blood pressure (WHO, 2008) and the prevalence of daily smoking of any tobacco product in men, Figure S31: Relationship between the prevalence of women’s raised blood pressure (WHO, 2008) and the prevalence of daily smoking of any tobacco product in women, Figure S32: Relationship between the prevalence of men’s CVD mortality (WHO, 2012) and the prevalence of current smoking of any tobacco product in men, Figure S33: Relationship between the prevalence of women’s CVD mortality (WHO, 2012) and the prevalence of current smoking of any tobacco product in women, Figure S34: Relationship between the self-reported prevalence of physical activity (WHO, 2010) and men’s CVD mortality (WHO, 2012), Figure S35: Correlation between the self-reported prevalence of physical activity and women‘s CVD mortality (WHO, 2012), Figure S36: Relationship between the prevalence of men’s raised blood pressure (%; WHO, 2010) and the mean consumption of sunflower oil (FAOSTAT, 1993–2011), Figure S37: Relationship between the prevalence of women’s raised blood pressure (%; WHO, 2010) and the mean consumption of sunflower oil (FAOSTAT, 1993–2011), Figure S38: Relationship between men’s CVD mortality (WHO, 2012) and the mean consumption of sunflower oil (FAOSTAT, 1993–2011), Figure S39: Relationship between women’s CVD mortality (WHO, 2012) and the mean consumption of sunflower oil (FAOSTAT, 1993–2011), Figure S40: Factor analysis including 75 variables in 92 countries with health expenditure above 500 USD per capita explaining 36.1% variability (Factor 1 vs. Factor 2), Figure S41: Factor analysis including 75 variables in 92 countries with health expenditure above 500 USD per capita explaining 35.2% variability (Factor 1 vs. Factor 3), Figure S42: Factor analysis including 75 variables in 61 countries with health expenditure 500–2000 USD per capita explaining 30.5% variability, Figure S43: Factor analysis including 77 variables (plus smoking) in 115 countries explaining 42.1% variability. Smoking in this plot represents ‘Current smoking of any tobacco product’, Figure S44: Factor analysis including 75 variables in 116 non-European countries explaining 38.1% variability, Figure S45: Factor analysis including 75 variables in 51 non-European countries with health expenditure above 500 USD per capita explaining 35.3% variability, Figure S46: Relationship between the prevalence of men’s raised blood pressure (%; WHO, 2010) and the mean consumption of potatoes (FAOSTAT, 1993–2011), Figure S47: Relationship between the prevalence of women’s raised blood pressure (%; WHO, 2010) and the mean consumption of dairy (FAOSTAT, 1993–2011), Figure S48: Relationship between men’s CVD mortality (WHO, 2012) and the mean consumption of alcoholic beverages (FAOSTAT, 1993–2011), Figure S49: Relationship between women’s CVD mortality (WHO, 2012) and the mean consumption of distilled beverages (FAOSTAT, 1993–2011), Figure S50: Temporal changes in correlation coefficients (*r*-values) between men’s raised blood pressure (2010) and 10 food items, Figure S51: Temporal changes in correlation coefficients (*r*-values) between women’s raised blood pressure (2010) and 10 food items, Table S1: Relationship between raised blood pressure and the examined variables (total sample of 158 countries), Table S2: Relationship between raised blood pressure and the examined variables (the world outside Europe, 116 countries), Table S3: Relationship between raised cholesterol and the examined variables (total sample of 158 countries), Table S4: Relationship between raised cholesterol and the examined variables (the world outside Europe, 116 countries), Table S5: Relationship between CVD mortality and the examined variables (total sample of 158 countries), Table S6: Relationship between CVD mortality and the examined variables (the world outside Europe, 116 countries), Table S7: Relationship between raised blood glucose and the examined variables (total sample of 158 countries), Table S8: Relationship between raised blood glucose and the examined variables (the world outside Europe, 116 countries), Table S9: Correlation between smoking and health indicators. All countries (*n* = 115), Table S10: Correlation between smoking and health indicators. Non-European countries (*n* = 76), Table S11: Correlation between physical activity and health indicators in 123 countries, Supplementary dataset, sheet S1: Plant food, sheet S2: Animal food, Total, sheet S3: Health statistics, sheet S4: Raised blood pressure (women), sheet S5: CVD mortality (women), sheet S6: Raised blood glucose (women).
