# Supplementary material for: Global Correlates of Cardiovascular Risk: A Comparison of 158 Countries
Source: Nutrients. 2018 Mar 26;10(4):411. doi: 10.3390/nu10040411 (PMC5946196; doi:10.3390/nu10040411)
Supplement: Supplementary file 1 [file nutrients-10-00411-s001.zip › Supplementary tables and figures_Global correlates of CVD risk.pdf]

Supplementary tables and figures

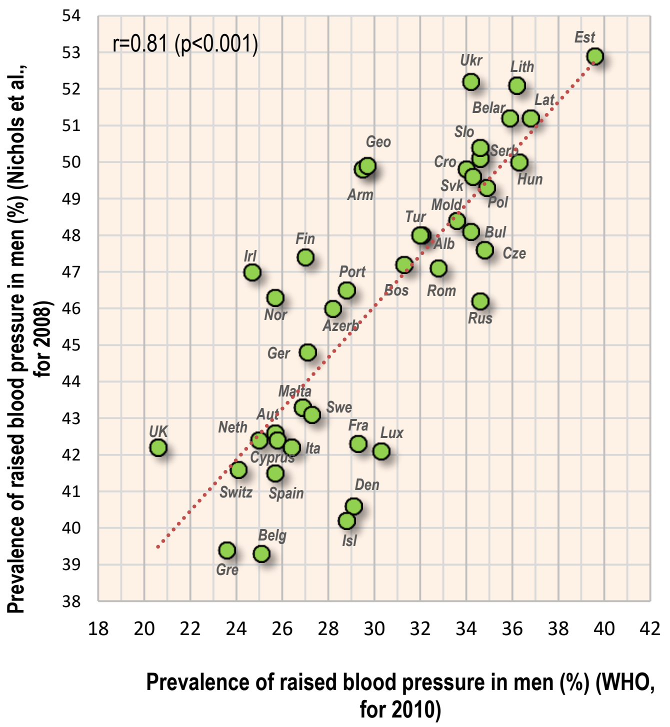

Figure S1. Correlation between the prevalence of men's raised pressure (%) by WHO (for 2010) and by Nichols et al. (for 2008, including people using blood pressure medications).

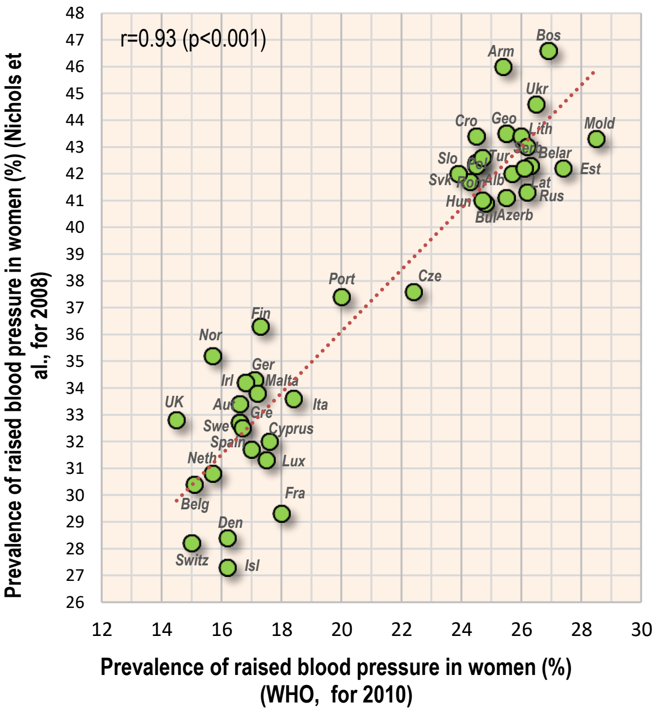

Figure S2. Correlation between the prevalence of women's raised pressure by WHO (for 2010) and by Nichols et al. (for 2008, including people using blood pressure medications)

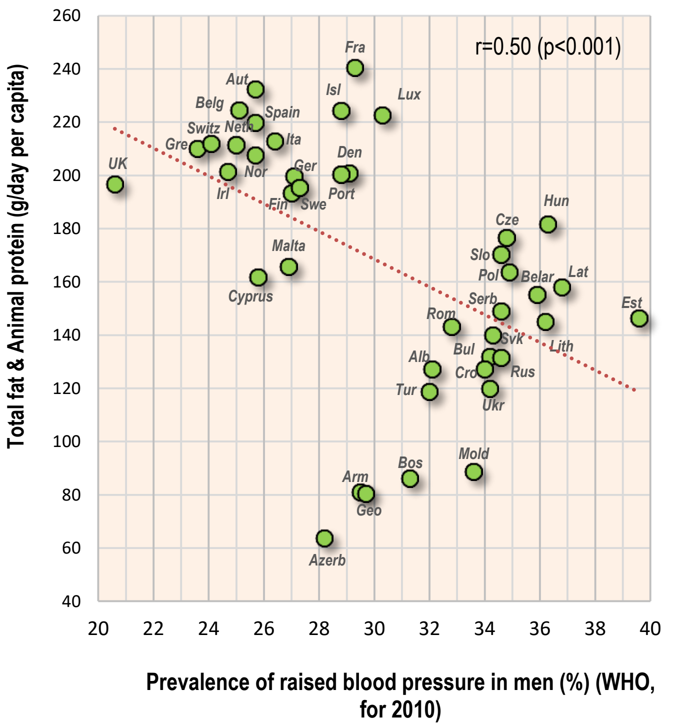

__Figure S3.__ Correlation between the prevalence of men's raised pressure (%) by WHO (for 2010, ***excluding people using medications***) and the mean consumption of total fat & animal protein (g/day per capita; FAOSTAT, 1993-2011).

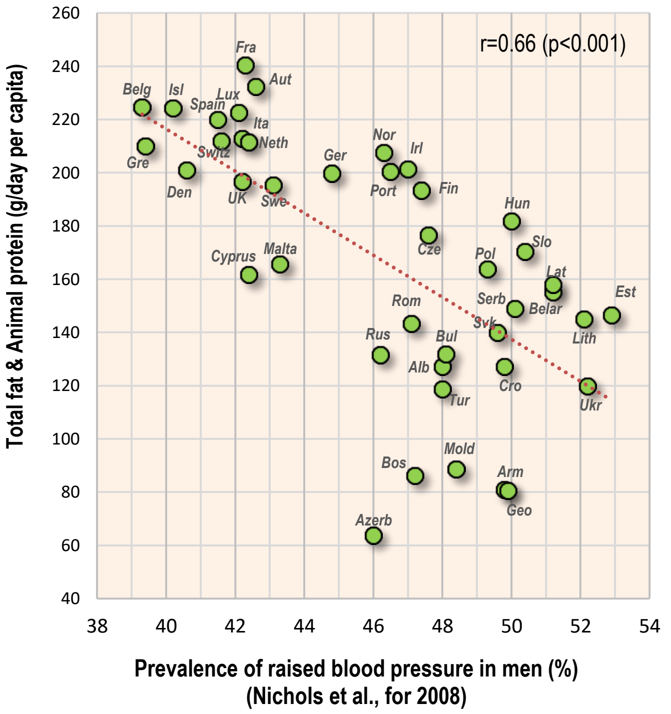

__Figure S4__. Correlation between the prevalence of men's raised pressure by Nichols et al. (for 2008, ***including people using medications***) and the mean consumption of total fat & animal protein (g/day per capita; FAOSTAT, 1993-2011).

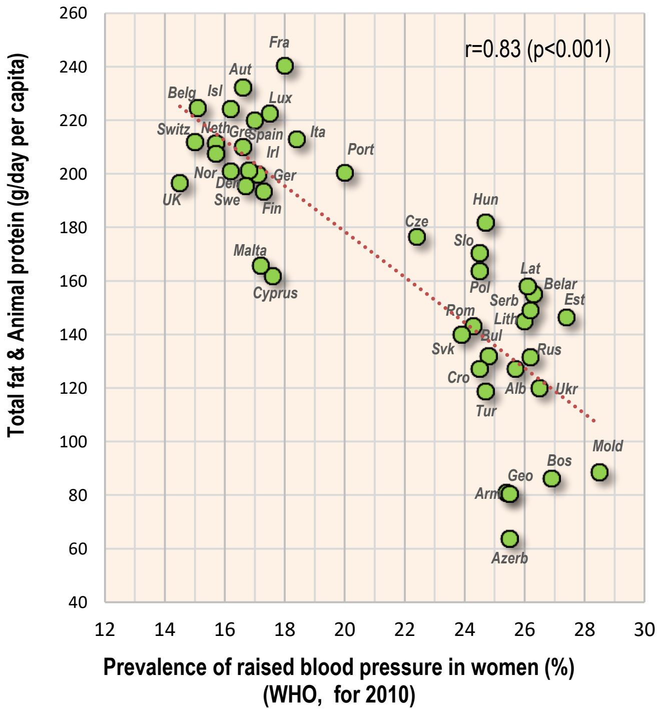

__Figure S5.__ Correlation between the prevalence of women's raised pressure (%) by WHO (for 2010, ***excluding people using medications***) and the mean consumption of total fat & animal protein (g/day per capita; FAOSTAT, 1993-2011).

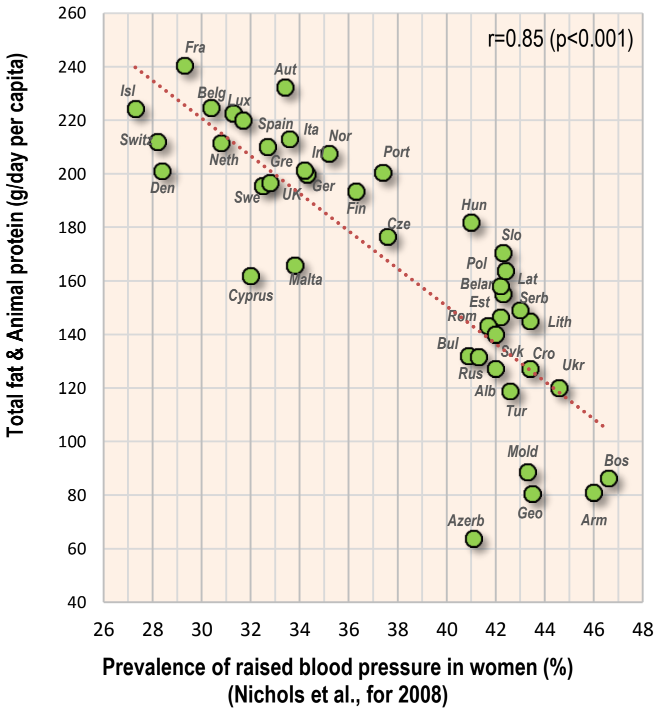

__Figure S6__. Correlation between the prevalence of women's raised pressure by Nichols et al. (for 2008, ***including people using medications***) and the mean consumption of total fat & animal protein (g/day per capita; FAOSTAT, 1993-2011).

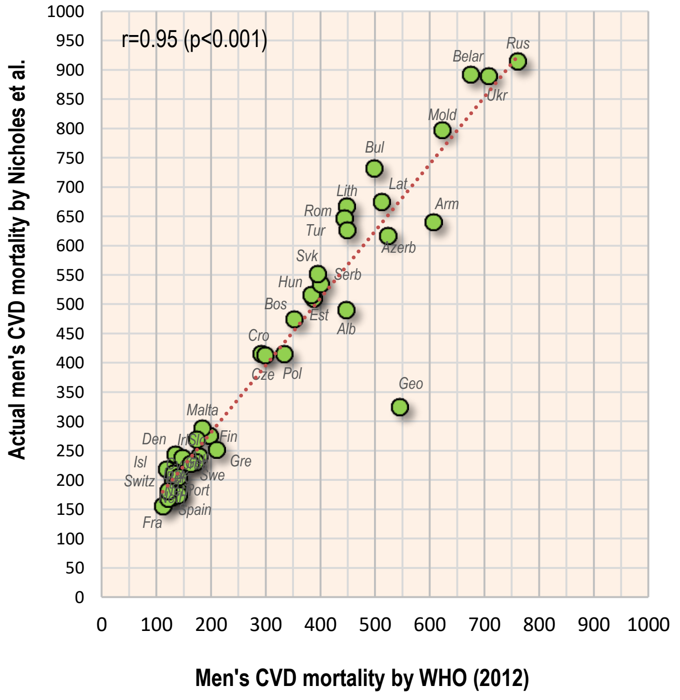

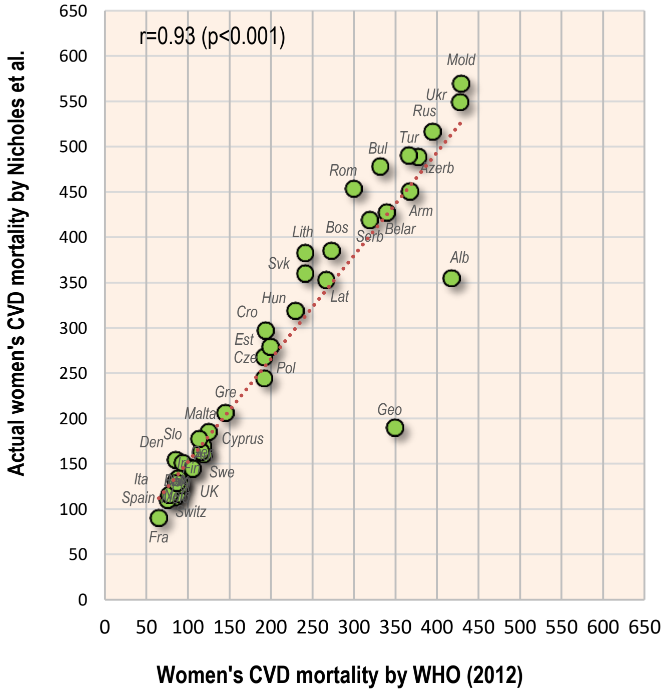

Figure S7. Correlation between men's CVD mortality by WHO (2012) and the actual men's CVD mortality in by Nichols et al. in 42 European countries (per 100,000 population).

Figure S8. Correlation between women's CVD mortality by WHO (2012) and the actual women's CVD mortality in by Nichols et al. in 42 European countries (per 100,000 population).

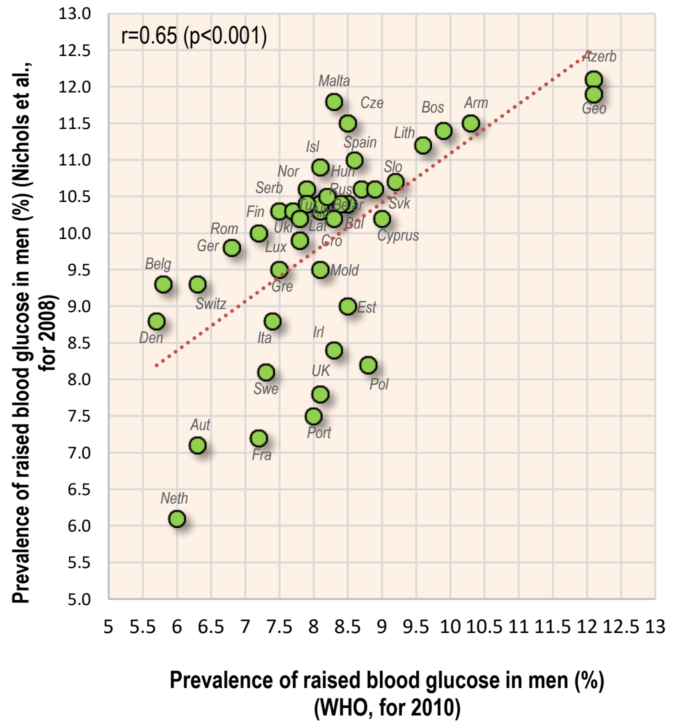

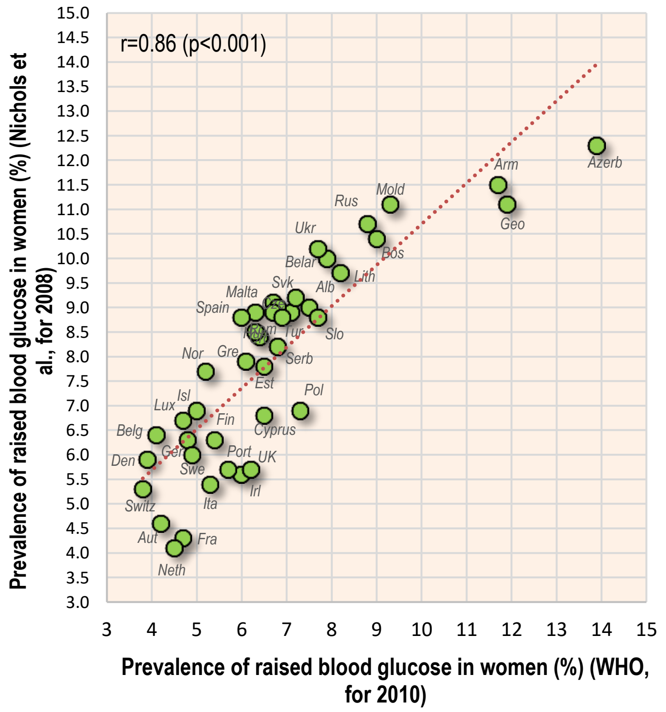

Figure S9. Correlation between the prevalence of men's raised glucose (%) by WHO (for 2010) and by Nichols et al. (for 2008) in 42 European countries.

Figure S10. Correlation between the prevalence of women's raised glucose by WHO (for 2010) and by Nichols et al. (for 2008) in 42 European countries.

Raised blood pressure

Table S1. Relationship between raised blood pressure and the examined variables (total sample of 158 countries).

Raised blood pressure (Men)			Raised blood pressure (Women)		
**Positive correlates**	**Mean**	**Correlation & p-values**	**Positive correlates**	**Mean**	**Correlation & p-values**
CVD MORTALITY	*308.0*	0.42 (p<0.001)	*% CC energy & % SRC energy*	*42.6*	0.69 (p<0.001)
Sunflower oil	5.2	0.29 (p=0.013)	*CVD MORTALITY*	*241.7*	0.69 (p<0.001)
Starchy roots total	201.0	0.20 (p=0.010	*% Plant food energy*	*79.8*	0.64 (p<0.001)
% SRC energy	*6.2*	0.18 (p=0.023)	*% CA energy*	*64.1*	0.62 (p<0.001)
Potatoes	98.7	0.16 (p=0.039)	*% CC energy*	*36.4*	0.50 (p<0.001)
Offals	7.6	0.16 (p=0.041)	*% SRC energy*	*6.2*	0.35 (p<0.001)
Milk	169.0	0.15 (p=0.053)	Legumes (excl. Soybeans)	16.0	0.28 (p<0.001)
% Alcoholic beverages energy	168.9	0.15 (p=0.06)	*RAISED BLOOD GLUCOSE (%)*	*8.8*	0.28 (p<0.001)
Distilled beverages	7.7	0.12 (p=0.14)	Maize	60.2	0.27 (p<0.001)
% CC energy & % SRC energy	*42.6*	0.11 (p=0.16)	Cereals total	373.7	0.26 (p<0.001)
**Negative correlates**	**Mean**	**Correlation & p-values**	**Negative correlates**	**Mean**	**Correlation & p-values**
LIFE EXPECTANCY	*68.2*	-0.39 (p<0.001)	*LIFE EXPECTANCY*	*73.0*	-0.79 (p<0.001)
Oranges & mandarins	38.9	-0.33 (p<0.001)	*RAISED CHOLESTEROL (%)*	*40.5*	-0.73 (p<0.001)
Fruits total	204.9	-0.32 (p<0.001)	Animal protein	32.8	-0.71 (p<0.001)
Rice	79.7	-0.31 (p<0.001)	*HEALTH EXPENDITURE*	*1254.9*	-0.71 (p<0.001)
Poultry	38.4	-0.28 (p<0.001)	Animal fat & Animal protein	68.6	-0.70 (p<0.001)
Oilcrops	16.5	-0.28 (p<0.001)	Total fat & Animal protein	111.3	-0.70 (p<0.001)
HEALTH EXPENDITURE	*1254.9*	-0.27 (p<0.001)	Meat total	117.7	-0.70(p<0.001)
Fish & seafood	43.2	-0.27 (p<0.001)	Meat protein	14.9	-0.67 (p<0.001)
Tree nuts	5.2	-0.24 (p=0.002)	Animal fat	35.9	-0.67 (p<0.001)
Ref. sugar & sweeteners total	80.1	-0.21 (p=0.006)	Total fat & Total protein	154.4	-0.66 (p<0.001)

Table S2. Relationship between raised blood pressure and the examined variables (the world outside Europe, 116 countries).

Raised blood pressure (Men)			Raised blood pressure (Women)		
**Positive correlates**	**Mean**	**Correlation & p-values**	**Positive correlates**	**Mean**	**Correlation & p-values**
% CC energy & % SRC energy	*46.7*	0.33 (p<0.001)	*% CC energy & % SRC energy*	*11.6*	0.62 (p<0.001)
CVD MORTALITY	*305.5*	0.30 (p<0.001)	*% Plant food energy*	*8.2*	0.62 (p<0.001)
% Plant food energy	*84.4*	0.30 (p<0.001)	*CVD MORTALITY*	*114.0*	0.61 (p<0.001)
% SRC energy	*7.0*	0.28 (p=0.004)	*% CA energy*	*6.8*	0.50 (p<0.001)
Mutton & goat meat	9.9	0.23 (p=0.015)	*% CC energy*	*11.5*	0.36 (p<0.001)
% CA energy	*67.1*	0.21 (p=0.021)	*% SRC energy*	*9.2*	0.34 (p<0.001)
Starchy roots total	196.4	0.21 (p=0.027)	Fermented beverages	68.6	0.25 (p=0.006)
Fermented beverages	22.0	0.20 (p=0.032)	Starchy roots	215.7	0.25 (p=0.007)
% CC energy	*39.7*	0.12 (p=0.21)	Legumes (excl. Soybeans)	14.3	0.22 (p=0.019)
Legumes (excl. Soybeans)	19.2	0.12 (p=0.21)	Maize	87.5	0.19 (p=0.040)
**Negative correlates**	**Mean**	**Correlation & p-values**	**Negative correlates**	**Mean**	**Correlation & p-values**
LIFE EXPECTANCY	*65.9*	-0.65 (p<0.001)	*LIFE EXPECTANCY*	*70.2*	-0.80 (p<0.001)
Eggs total	12.6	-0.52 (p<0.001)	*RAISED CHOLESTEROL (%)*	*36.0*	-0.72 (p<0.001)
Pork	14.8	-0.51 (p<0.001)	Eggs total	12.6	-0.70 (p<0.001)
HEALTH EXPENDITURE	*767.0*	-0.50 (p<0.001)	Animal fat & Animal protein	50.6	-0.68 (p<0.001)
RAISED CHOLESTEROL (%)	*32.7*	-0.47 (p<0.001)	Animal protein	25.5	-0.68 (p<0.001)
Coffee	3.2	-0.45 (p<0.001)	Animal fat	25.1	-0.65 (p<0.001)
Ref. sugar & sweeteners total	71.2	-0.44 (p<0.001)	Meat total	93.3	-0.65 (p<0.001)
Beer	43.9	-0.39 (p<0.001)	Pork	14.8	-0.64 (p<0.001)
Poultry	36.3	-0.38 (p<0.001)	Ref. sugar & sweeteners total	71.2	-0.64 (p<0.001)
Apples	13.4	-0.37 (p<0.001)	Total fat & Animal protein	91.1	-0.64 (p<0.001)

Level of significance:

Positive correlates		Negative correlates	
p≥0.001	p<0.001	p≥0.001	p<0.001

Abbreviations: % CC energy = the mean proportion of carbohydrate energy from cereals; % SRC energy = the mean proportion of carbohydrate energy from starchy roots; % CA energy = the mean proportion of energy from carbohydrates and alcohol.

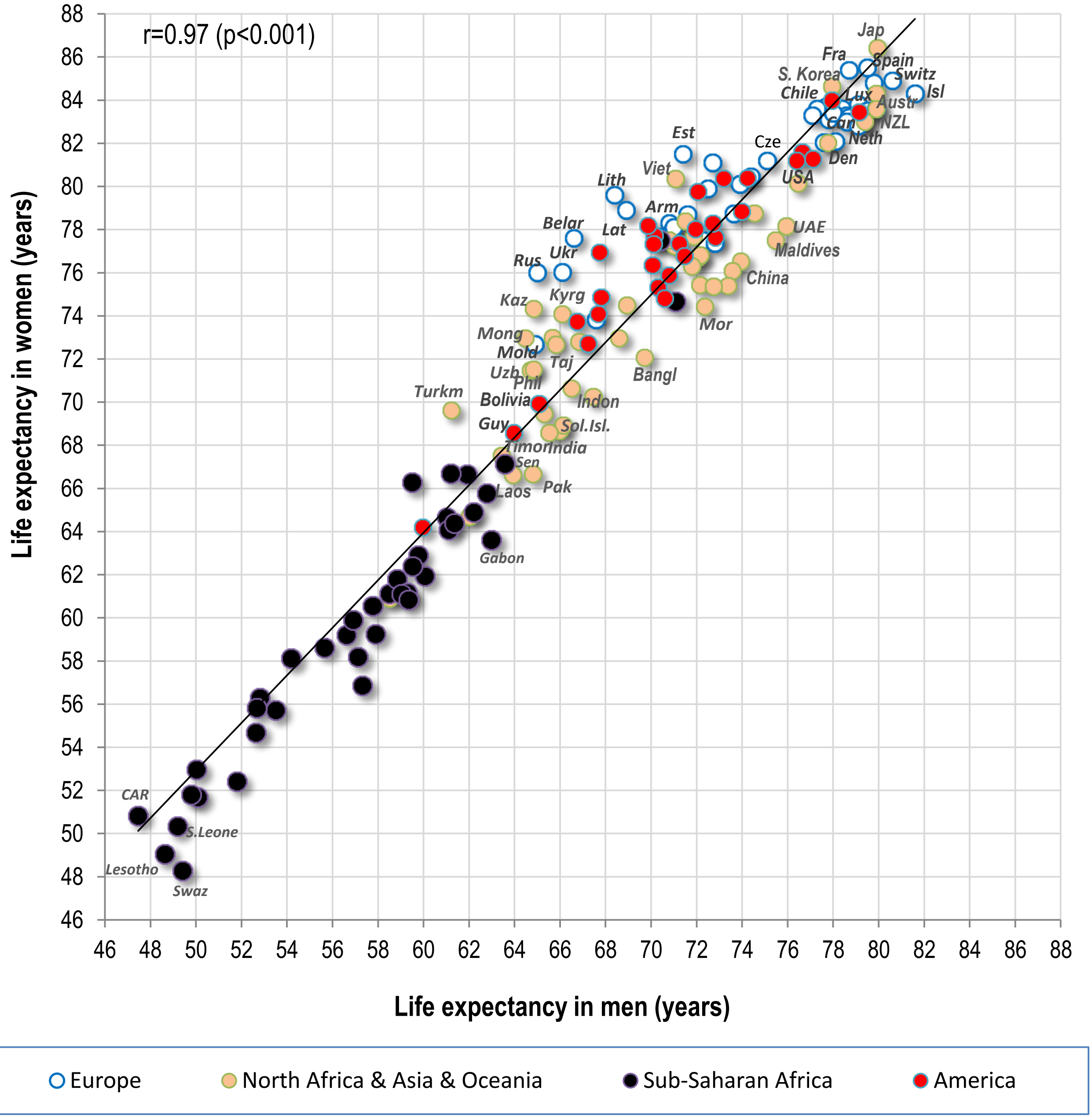

Figure S11. Relationship between life expectancy in men and women (WHO, 2012).

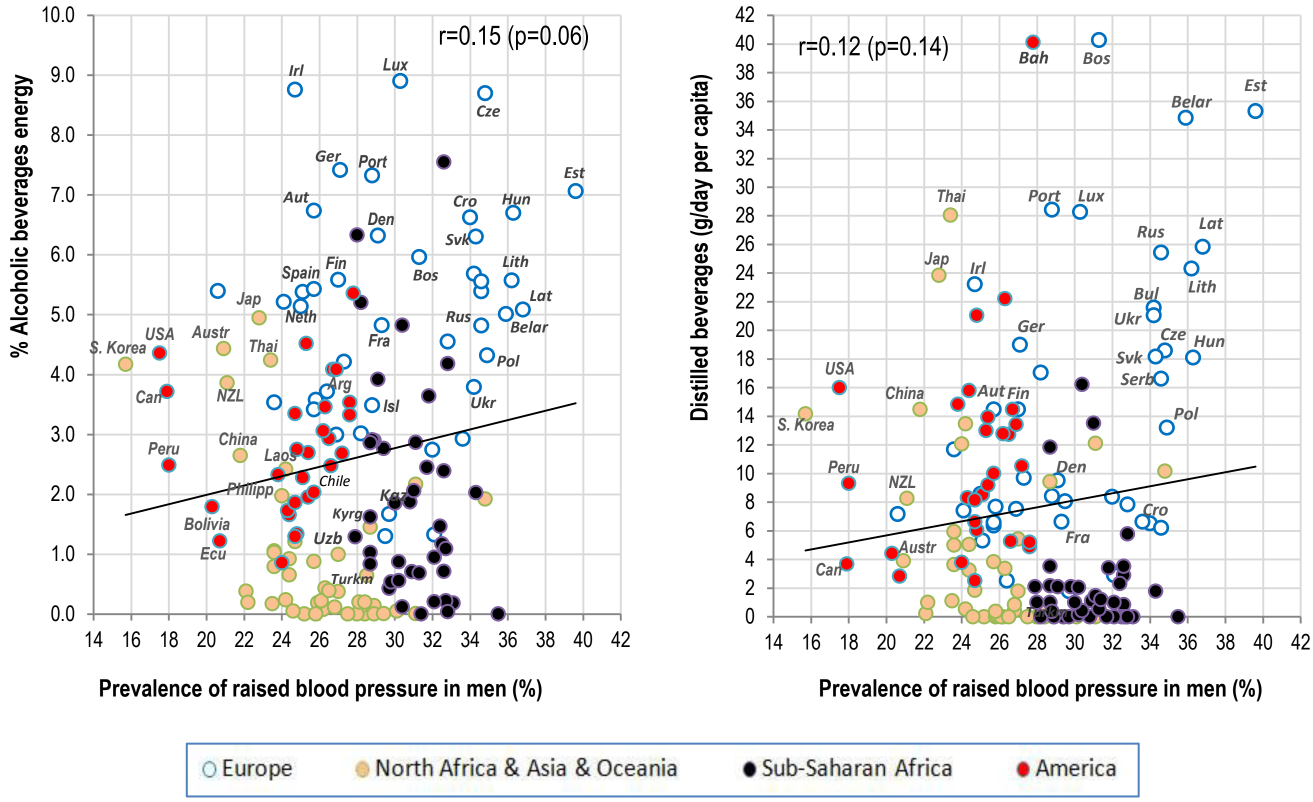

Figure S12. Relationship between the prevalence of men's raised blood pressure (%; WHO, 2010) and the mean proportion of energy from alcoholic beverages (%; FAOSTAT, 1993-2011).

Figure S13. Relationship between the prevalence of men's raised blood pressure (%; WHO, 2010) and the mean consumption of distilled beverages (g/day per capita; (FAOSTAT, 1993-2011).

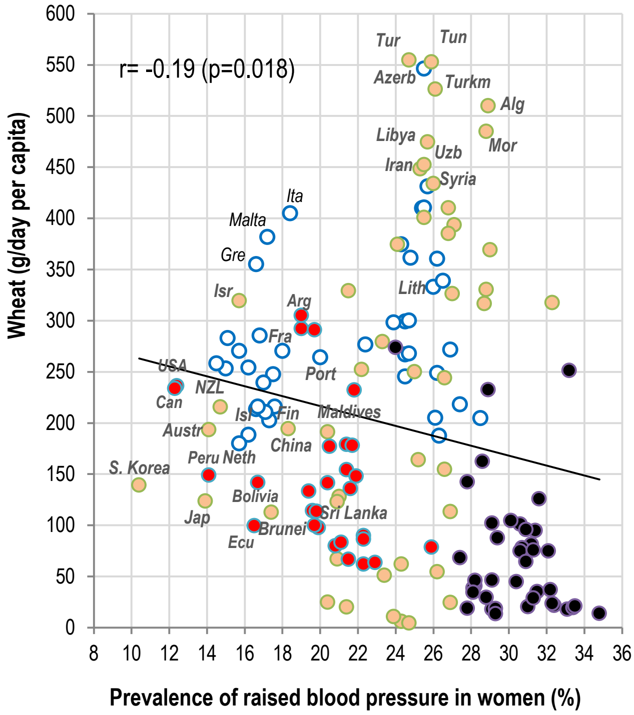

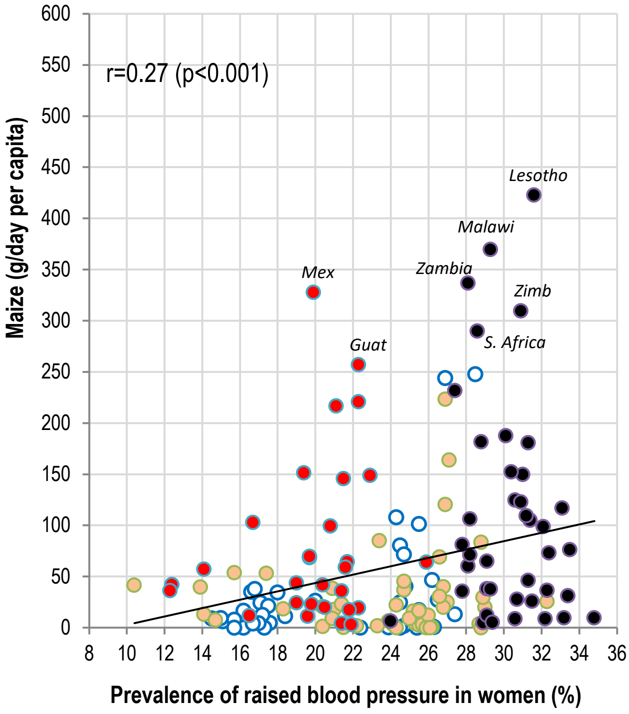

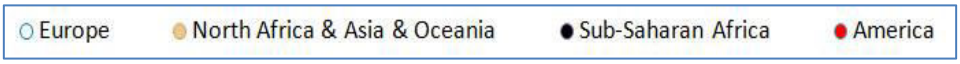

Figure S14. Relationship between the prevalence of women's raised blood pressure (%; WHO, 2010) and the mean consumption of wheat (g/day per capita; FAOSTAT, 1993-2011).

Figure S15. Relationship between the prevalence of women's raised blood pressure (%; WHO, 2010) and the mean consumption of maize (g/day per capita; FAOSTAT, 1993-2011).

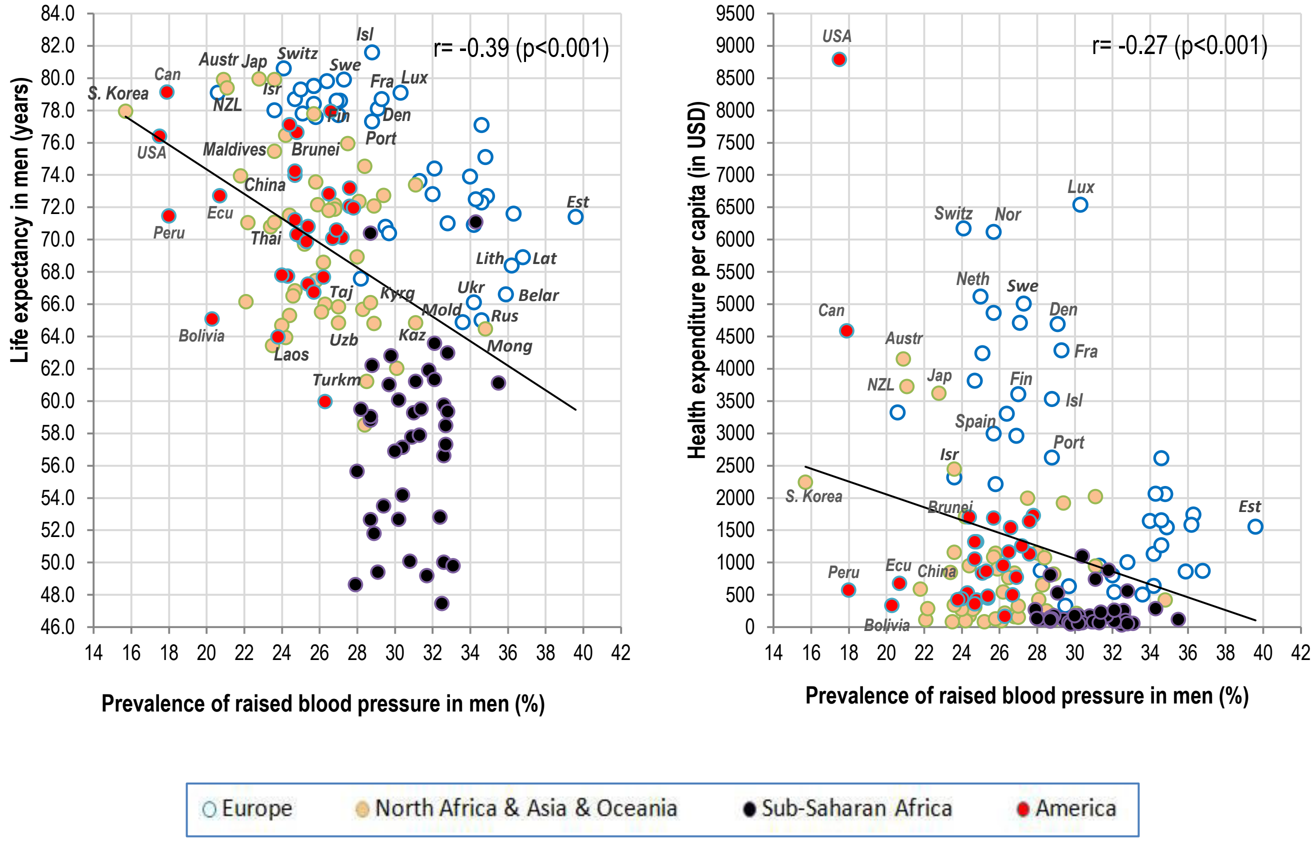

Figure S16. Relationship between the prevalence of men's raised blood pressure (%; WHO, 2010) and men's life expectancy (World Bank, 2012).

Figure S17. Relationship between the prevalence of men's raised blood pressure (%; WHO, 2010) and health expenditure per capita for 2012 (in USD).

Raised cholesterol

Table S3. Relationship between raised cholesterol and the examined variables (total sample of 158 countries).

Raised cholesterol (Men)			Raised cholesterol (Women)		
**Positive correlates**	**Mean**	**Correlation & p-values**	**Positive correlates**	**Mean**	**Correlation & p-values**
Animal fat & Animal protein	68.6	0.89 (p<0.001)	Animal fat & Animal protein	68.6	0.85 (p<0.001)
Animal protein	32.8	0.88 (p<0.001)	Animal protein	32.8	0.85 (p<0.001)
Animal fat	35.9	0.87 (p<0.001)	Total fat & Animal protein	111.3	0.84 (p<0.001)
Total fat & Animal protein	111.3	0.87 (p<0.001)	*LIFE EXPECTANCY*	*73.0*	0.83 (p<0.001)
Total fat & Total protein	154.4	0.86 (p<0.001)	Total fat & Total protein	154.4	0.83 (p<0.001)
Total fat	78.6	0.84 (p<0.001)	Animal fat	35.9	0.83 (p<0.001)
Meat total	117.7	0.82 (p<0.001)	Total protein	75.8	0.80 (p<0.001)
Total protein	75.8	0.82 (p<0.001)	Meat total	117.7	0.80 (p<0.001)
Meat protein	14.9	0.81 (p<0.001)	Total fat	78.6	0.80 (p<0.001)
LIFE EXPECTANCY	*68.2*	0.80 (p<0.001)	Eggs total	17.1	0.79 (p<0.001)
**Negative correlates**	**Mean**	**Correlation & p-values**	**Negative correlates**	**Mean**	**Correlation & p-values**
% Plant food energy	*79.8*	-0.84 (p<0.001)	*% Plant food energy*	*79.8*	-0.80 (p<0.001)
% CC energy & % SRC energy	*42.6*	-0.79 (p<0.001)	*% CC energy & % SRC energy*	*42.6*	-0.79 (p<0.001)
% CA energy	*64.1*	-0.79 (p<0.001)	*% CA energy*	*64.1*	-0.75 (p<0.001)
% CC energy	*36.4*	-0.59 (p<0.001)	*RAISED BLOOD PRESSURE (%)*	*24.0*	-0.73 (p<0.001)
Legumes (excl. Soybeans)	16.0	-0.41 (p<0.001)	*% CC energy*	*36.4*	-0.57 (p<0.001)
Legumes (incl. Soybeans)	17.2	-0.40 (p<0.001)	*CVD MORTALITY*	*241.7*	-0.52 (p<0.001)
% SRC energy	*6.2*	-0.39 (p<0.001)	*% SRC energy*	*6.2*	-0.42 (p<0.001)
Maize	60.2	-0.36 (p<0.001)	Legumes (excl. Soybeans)	16.0	-0.39 (p<0.001)
Rice	79.7	-0.30 (p<0.001)	Legumes (incl. Soybeans)	17.2	-0.38 (p<0.001)
Fermented beverages	16.9	-0.29 (p<0.001)	Maize	60.2	-0.35 (p<0.001)

Table S4. Relationship between raised cholesterol and the examined variables (the world outside Europe, 116 countries).

Raised cholesterol (Men)			Raised cholesterol (Women)		
**Positive correlates**	**Mean**	**Correlation & p-values**	**Positive correlates**	**Mean**	**Correlation & p-values**
Animal fat & Animal protein	50.6	0.78 (p<0.001)	*LIFE EXPECTANCY*	*70.2*	0.78 (p<0.001)
Animal protein	25.5	0.78 (p<0.001)	Animal protein	25.5	0.77 (p<0.001)
Total fat & Animal protein	91.1	0.76 (p<0.001)	Animal fat & Animal protein	50.6	0.76 (p<0.001)
LIFE EXPECTANCY	*65.9*	0.75 (p<0.001)	Total fat & Animal protein	91.1	0.74 (p<0.001)
Animal fat	25.1	0.74 (p<0.001)	Total fat & Total protein	134.2	0.73 (p<0.001)
Total fat & Total protein	134.2	0.74 (p<0.001)	Eggs total	12.6	0.73 (p<0.001)
Meat total	93.3	0.73 (p<0.001)	Poultry	36.3	0.72 (p<0.001)
Meat protein	12.1	0.72(p<0.001)	Meat total	93.3	0.72 (p<0.001)
Eggs total	12.6	0.71 (p<0.001)	Animal fat	25.1	0.71 (p<0.001)
OBESITY (%)	*10.6*	0.71 (p<0.001)	Total protein	68.6	0.71 (p<0.001)
**Negative correlates**	**Mean**	**Correlation & p-values**	**Negative correlates**	**Mean**	**Correlation & p-values**
% Plant food energy	*84.4*	-0.67 (p<0.001)	*RAISED BLOOD PRESSURE (%)*	*25.0*	-0.72 (p<0.001)
% CC energy & % SRC energy	*46.7*	-0.65 (p<0.001)	*% CC energy & % SRC energy*	*46.7*	-0.68 (p<0.001)
% CA energy	*67.1*	-0.61 (p<0.001)	*% Plant food energy*	*84.4*	-0.64 (p<0.001)
RAISED BLOOD PRESSURE (%)	*27.3*	-0.47 (p<0.001)	*% CA energy*	*67.1*	-0.59 (p<0.001)
% SRC energy	*7.0*	-0.41 (p<0.001)	*CVD MORTALITY*	*256.1*	-0.45 (p<0.001)
Starchy roots total	196.4	-0.34 (p<0.001)	*% SRC energy*	*7.0*	-0.43 (p<0.001)
% CC energy	*39.7*	-0.33 (p<0.001)	Starchy roots	196.4	-0.35 (p<0.001)
Fermented beverages	22.0	-0.30 (p=0.001)	*% CC energy*	*39.7*	-0.34 (p<0.001)
Maize	70.8	-0.26 (p=0.006)	Fermented beverages	22.0	-0.32 (p<0.001)
Legumes (excl. Soybeans)	19.2	-0.26 (p=0.007)	Legumes (excl. Soybeans)	19.2	-0.25 (p=0.007)

Level of significance:

**Positive correlates**		**Negative correlates**	
p < 0.05	p < 0.001	p < 0.05	p < 0.001

Abbreviations: % CC energy = the mean proportion of carbohydrate energy from cereals; % SRC energy = the mean proportion of carbohydrate energy from starchy roots; % CA energy = the mean proportion of energy from carbohydrates and alcohol.

CVD mortality

Table S5. Relationship between CVD mortality and the examined variables (total sample of 158 countries).

CVD mortality (Men)			CVD mortality (Women)		
**Positive correlates**	**Mean**	**Correlation (p-values)**	**Positive correlates**	**Mean**	**Correlation (p-values)**
Cereals total	373.7	0.42 (p<0.001)	*RAISED BLOOD PRESSURE (%)*	*24.0*	0.69 (p<0.001)
RAISED BLOOD PRESSURE (%)	28.1	0.42 (p<0.001)	*% CC energy & % SRC energy*	*42.6*	0.58 (p<0.001)
Wheat	197.0	0.37 (p<0.001)	*% CC energy*	*36.4*	0.51 (p<0.001)
% CC energy	*36.4*	0.35 (p<0.001)	*% CA energy*	*64.1*	0.47 (p<0.001)
% CC energy & % SRC energy	*42.6*	0.34 (p<0.001)	*% Plant food energy*	*79.8*	0.45 (p<0.001)
RAISED BLOOD GLUCOSE (%)	9.1	0.30 (p<0.001)	Cereals total	373.7	0.41 (p<0.001)
% CA energy	*64.1*	0.26 (p=0.001)	*RAISED BLOOD GLUCOSE (%)*	*8.8*	0.36 (p<0.001)
Plant protein	43.0	0.25 (p=0.002)	Plant protein	43.0	0.25 (p=0.001)
Sunflower oil	5.2	0.24 (p=0.002)	*% SRC energy*	*6.2*	0.16 (p=0.040)
Milk	169.0	0.21 (p=0.009)	Wheat	197.0	0.16 (p=0.049)

**Negative correlates**	**Mean**	**Correlation (p-values)**	**Negative correlates**	**Mean**	**Correlation (p-values)**
HEALTH EXPENDITURE	*1254.9*	-0.43 (p<0.001)	*HEALTH EXPENDITURE*	*1254.9*	-0.59 (p<0.001)
Oranges & mandarins	38.9	-0.41 (p<0.001)	Coffee	5.6	-0.55 (p<0.001)
Coffee	5.6	-0.40 (p<0.001)	Total fat & Animal protein	111.3	-0.54 (p<0.001)
Fruits total	204.9	-0.38 (p<0.001)	*LIFE EXPECTANCY*	*73.0*	-0.54 (p<0.001)
Cheese	11.5	-0.35 (p<0.001)	Meat total	117.7	-0.53 (p<0.001)
LIFE EXPECTANCY	*68.2*	-0.33 (p<0.001)	Total fat	78.6	-0.53 (p<0.001)
Plant fat	42.7	-0.32 (p<0.001)	Oranges & Mandarins	38.9	-0.53 (p<0.001)
Beer	77.6	-0.31 (p<0.001)	Ref. sugar & sweeteners total	80.1	-0.53 (p<0.001)
Alcoholic beverages total	118.8	-0.31 (p<0.001)	Animal protein	32.8	-0.53 (p<0.001)
Total fat	78.6	-0.30 (p<0.001)	Cheese	11.5	-0.53 (p<0.001)

Table S6. Relationship between CVD mortality and the examined variables (the world outside Europe, 116 countries).

CVD mortality (Men)			CVD mortality (Women)		
**Positive correlates**	**Mean**	**Correlation (p-values)**	**Positive correlates**	**Mean**	**Correlation (p-values)**
Wheat	164.4	0.44 (p<0.001)	*RAISED BLOOD PRESSURE (%)*	*25.0*	0.61 (p<0.001)
Cereals total	377.6	0.37 (p<0.001)	*% CC energy & % SRC energy*	*46.7*	0.50 (p<0.001)
Mutton & Goat meat	9.9	0.34 (p<0.001)	*% CC energy*	*39.7*	0.39 (p<0.001)
RAISED BLOOD GLUCOSE (%)	*9.4*	0.31 (p<0.001)	*% Plant food energy*	*84.4*	0.35 (p<0.001)
% CC energy	*39.7*	0.31 (p<0.001)	Cereals total	377.6	0.33 (p<0.001)
RAISED BLOOD PRESSURE (%)	*27.3*	0.30 (p=0.001)	*% CA energy*	*67.1*	0.29 (p=0.001)
% CC energy & % SRC energy	*46.7*	0.29 (p=0.002)	Mutton & Goat meat	9.9	0.23 (p=0.008)
Grapes	9.1	0.25 (p=0.006)	Wheat	164.4	0.23 (p=0.015)
Plant protein	43.1	0.22 (p=0.016)	Plant protein	43.1	0.22 (p=0.017)
Milk	134.1	0.20 (p=0.027)	*RAISED BLOOD GLUCOSE (%)*	*9.6*	0.19 (p=0.020)

**Negative correlates**	**Mean**	**Correlation (p-values)**	**Negative correlates**	**Mean**	**Correlation (p-values)**
Beer	43.9	-0.38 (p<0.001)	Ref. sugar & sweeteners total	71.2	-0.52 (p<0.001)
Coffee	3.2	-0.36 (p<0.001)	*LIFE EXPECTANCY*	*70.2*	-0.51 (p<0.001)
HEALTH EXPENDITURE	*767.0*	-0.32 (p<0.001)	Beer	43.9	-0.50 (p<0.001)
Pork	14.8	-0.30 (p<0.001)	Eggs total	12.6	-0.48 (p<0.001)
Ref. sugar & sweeteners total	71.2	-0.30 (p=0.001)	Poultry	36.3	-0.47 (p<0.001)
Poultry	36.3	-0.29 (p=0.001)	Coffee	3.2	-0.47 (p<0.001)
Fruits total	192.2	-0.29 (p=0.001)	Refined sugar	59.4	-0.46 (p<0.001)
Eggs total	12.6	-0.29 (p=0.001)	Pork	14.8	-0.45 (p<0.001)
Alcoholic beverages total	76.2	-0.29 (p=0.002)	*HEALTH EXPENDITURE*	*767.0*	-0.45 (p<0.001)
Cheese	5.1	-0.27 (p=0.004)	*RAISED CHOLESTEROL (%)*	*36.0*	-0.45 (p<0.001)

Level of significance:

**Positive correlates**		**Negative correlates**	
**p < 0.05**	**p < 0.001**	**p < 0.05**	**p < 0.001**

Abbreviations: % CC energy = the mean proportion of carbohydrate energy from cereals; % SRC energy = the mean proportion of carbohydrate energy from starchy roots; % CA energy = the mean proportion of energy from carbohydrates and alcohol.

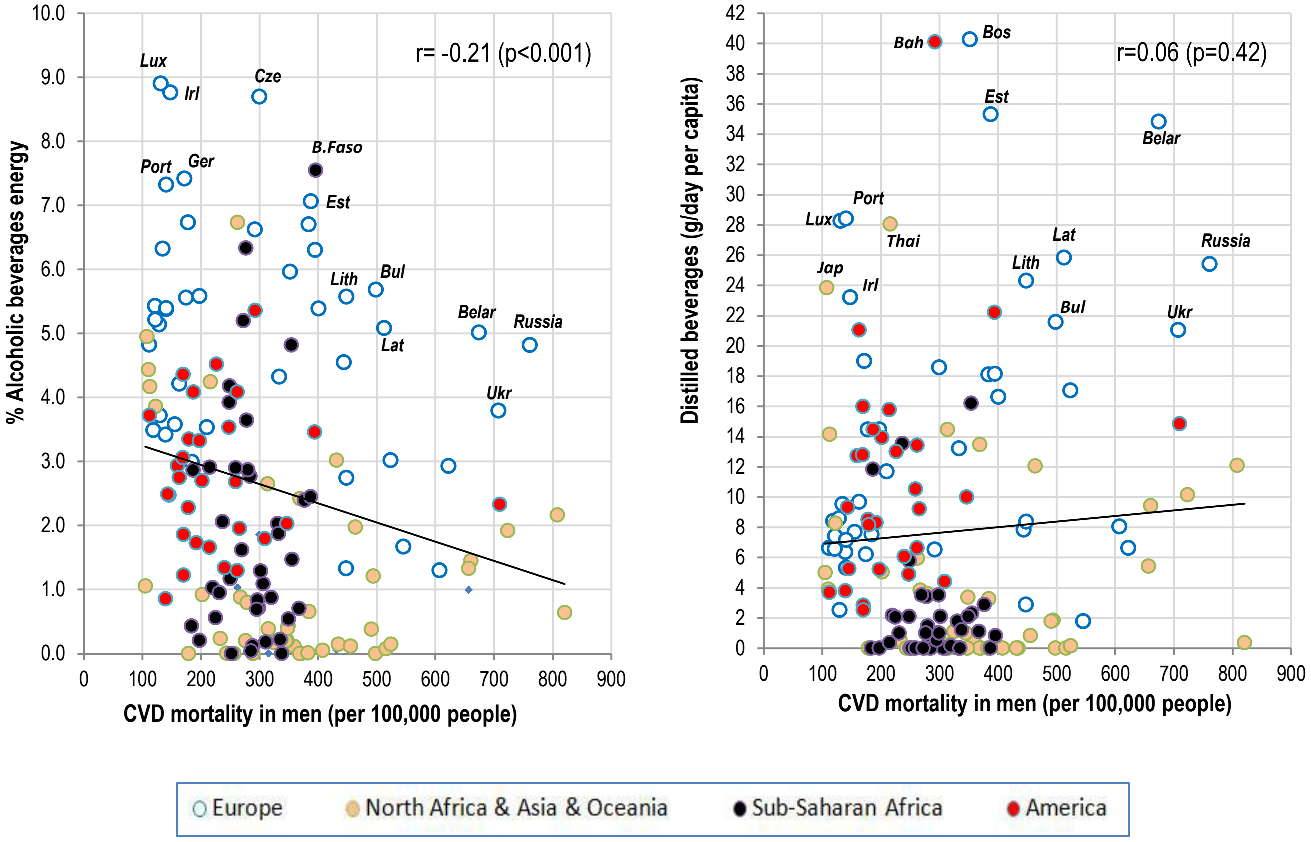

Figure S18. Relationship between men's CVD mortality (WHO, 2012) and the mean proportion of energy from alcoholic beverages (%; FAOSTAT, 1993-2011).

Figure S19. Relationship between men's CVD mortality (WHO, 2012) and the mean consumption of distilled beverages (g/day per capita; (FAOSTAT, 1993-2011).

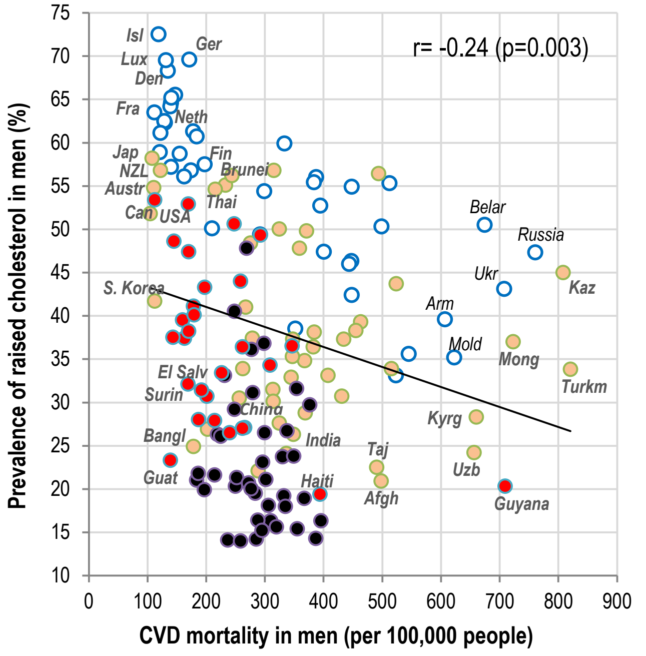

Figure S20. Relationship between men's CVD mortality (WHO, 2012) and the prevalence of men's raised cholesterol (%; WHO, 2010).

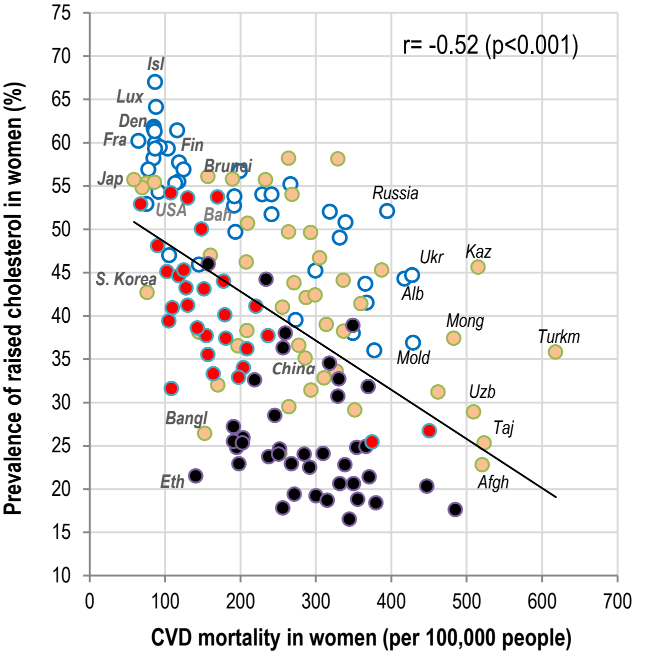

Figure S21. Relationship between women's CVD mortality (WHO, 2012) and the prevalence of women's raised cholesterol (%; WHO, 2010).

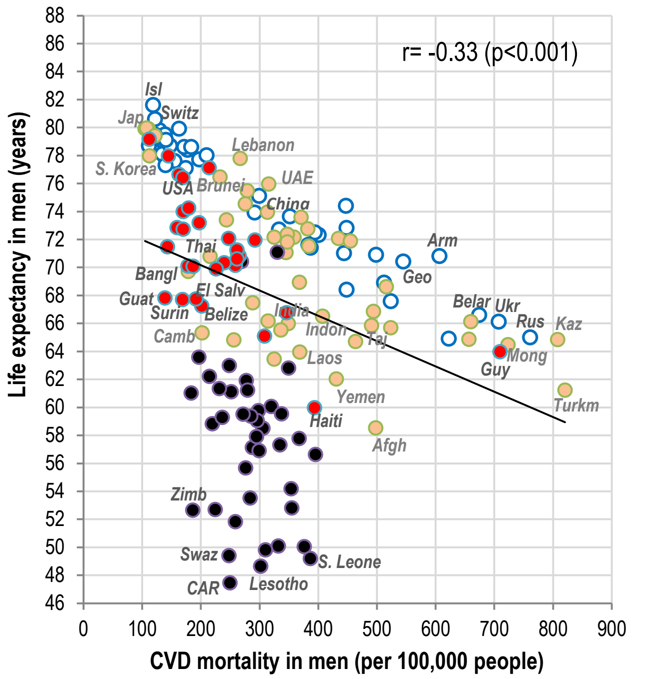

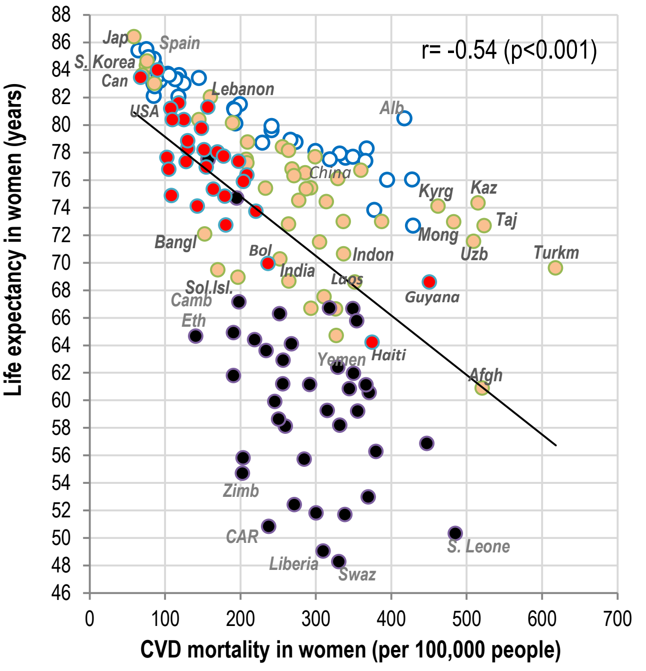

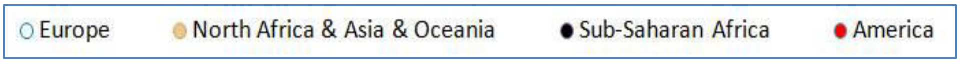

Figure S22. Relationship between men's CVD mortality (WHO, 2012) and men's life expectancy (World Bank, 2012).

Figure S23. Relationship between women's CVD mortality (WHO, 2012) and women's life expectancy (World Bank, 2012).

Raised blood glucose

Table S7. Relationship between raised blood glucose and the examined variables (total sample of 158 countries).

Raised blood glucose (Men)			Raised blood glucose (Women)		
**Positive correlates**	**Mean**	**Correlation (p-values)**	**Positive correlates**	**Mean**	**Correlation (p-values)**
Wheat	197.0	0.43 (p<0.001)	*MEAN BMI*	25.5	0.62 (p<0.001)
MEAN BMI	*24.8*	0.40 (p<0.001)	*OBESITY (%)*	19.6	0.57 (p<0.001)
OBESITY (%)	*12.9*	0.39 (p<0.001)	Cereals total	373.7	0.40 (p<0.001)
Cereals total	373.7	0.35 (p<0.001)	*CVD MORTALITY*	241.7	0.36 (p<0.001)
Tomatoes	50.1	0.35 (p<0.001)	*% Plant food energy*	79.8	0.36 (p<0.001)
Plant protein	43.0	0.34 (p<0.001)	*% CC energy*	36.4	0.34 (p<0.001)
Mutton & Goat meat	9.5	0.32 (p<0.001)	Plant protein	43.0	0.33 (p<0.001)
CVD MORTALITY	*308.0*	0.30 (p<0.001)	*Wheat*	197.0	0.31 (p<0.001)
Poultry	38.4	0.30 (p<0.001)	*% CA energy*	64.1	0.30 (p<0.001)
Vegetables total	224.8	0.27 (p<0.001)	*RAISED BLOOD PRESSURE (%)*	24.0	0.28 (p<0.001)
**Negative correlates**	**Mean**	**Correlation (p-values)**	**Negative correlates**	**Mean**	**Correlation (p-values)**
% Alcoholic beverages energy	*2.6*	-0.36 (p<0.001)	Pork	33.6	-0.54 (p<0.001)
Alcoholic beverages total	118.8	-0.35 (p<0.001)	Alcoholic beverages total	118.8	-0.53 (p<0.001)
Pork	33.6	-0.33 (p<0.001)	*% Alcoholic beverages energy*	*2.6*	-0.51 (p<0.001)
Freshwater fish	6.7	-0.28 (p<0.001)	Beer	77.6	-0.44 (p<0.001)
Beer	77.6	-0.26 (p=0.001)	Coffee	5.6	-0.44 (p<0.001)
Wine	16.7	-0.24 (p=0.002)	Wine	16.7	-0.44 (p<0.001)
Coffee	5.6	-0.23 (p=0.004)	Cheese	11.5	-0.40 (p<0.001)
Lard	5.5	-0.23 (p=0.004)	*HEALTH EXPENDITURE*	*1254.9*	-0.38 (p<0.001)
Starchy roots total	201.0	-0.22 (p=0.006)	Lard	5.5	-0.36 (p<0.001)
% SRC energy	*6.2*	-0.20 (p=0.011)	Animal fat	35.9	-0.35 (p<0.001)

Table S8. Relationship between raised blood glucose and the examined variables (the world outside Europe, 116 countries).

Raised blood glucose (Men)			Raised blood glucose (Women)		
**Positive correlates**	**Mean**	**Correlation (p-values)**	**Positive correlates**	**Mean**	**Correlation (p-values)**
OBESITY (%)	*10.6*	0.65 (p<0.001)	*OBESITY (%)*	*19.0*	0.72 (p<0.001)
MEAN BMI	*24.2*	0.63 (p<0.001)	*MEAN BMI*	*25.4*	0.72 (p<0.001)
Wheat	164.4	0.59 (p<0.001)	Wheat	164.4	0.55 (p<0.001)
Tomatoes	41.8	0.52 (p<0.001)	Tomatoes	41.8	0.47 (p<0.001)
Total energy	2550.7	0.47 (p<0.001)	Refined sugar	59.4	0.40 (p<0.001)
RAISED CHOLESTEROL (%)	*32.7*	0.45 (p<0.001)	*RAISED CHOLESTEROL (%)*	*36.0*	0.38 (p<0.001)
Vegetables total	191.5	0.44 (p<0.001)	Total energy	2550.7	0.36 (p<0.001)
Total protein	68.6	0.43 (p<0.001)	Grapes	9.1	0.35 (p<0.001)
Grapes	9.1	0.40 (p<0.001)	Cereals total	377.6	0.34 (p<0.001)
Total fat & Total protein	134.2	0.40 (p<0.001)	Ref. sugar & sweeteners total	71.2	0.33 (p<0.001)
**Negative correlates**	**Mean**	**Correlation (p-values)**	**Negative correlates**	**Mean**	**Correlation (p-values)**
% Alcoholic beverages energy	*1.7*	-0.30 (p=0.001)	*% Alcoholic beverages energy*	*1.7*	-0.31 (p<0.001)
Freshwater fish	6.8	-0.29 (p=0.001)	Alcoholic beverages total	76.2	-0.31 (p<0.001)
% CA energy	*67.1*	-0.28 (p=0.002)	Freshwater fish	6.8	-0.30 (p<0.001)
% SRC energy	*7.0*	-0.27 (p=0.004)	Pork	14.8	-0.30 (p=0.001)
Alcoholic beverages total	76.2	-0.25 (p=0.007)	*% SRC energy*	*7.0*	-0.28 (p=0.002)
Maize	70.8	-0.24 (p=0.009)	Starchy roots	196.4	-0.24 (p=0.008)
% CC energy & % SRC energy	*46.7*	-0.24 (p=0.010)	Fermented beverages	22.0	-0.24 (p=0.011)
Fermented beverages	22.0	-0.23 (p=0.012)	*% CC energy & % SRC energy*	*46.7*	-0.18 (p=0.049)
Starchy roots total	196.4	-0.23 (p=0.014)	Beer	43.9	-0.17 (p=0.07)
Pork	14.8	-0.20 (p=0.029)	Rice	104.6	-0.16 (p=0.09)

Level of significance:

**Positive correlates**		**Negative correlates**	
**p < 0.05**	**p < 0.001**	**p < 0.05**	**p < 0.001**

Abbreviations: % CC energy = the mean proportion of carbohydrate energy from cereals; % SRC energy = the mean proportion of carbohydrate energy from starchy roots; % CA energy = the mean proportion of energy from carbohydrates and alcohol.

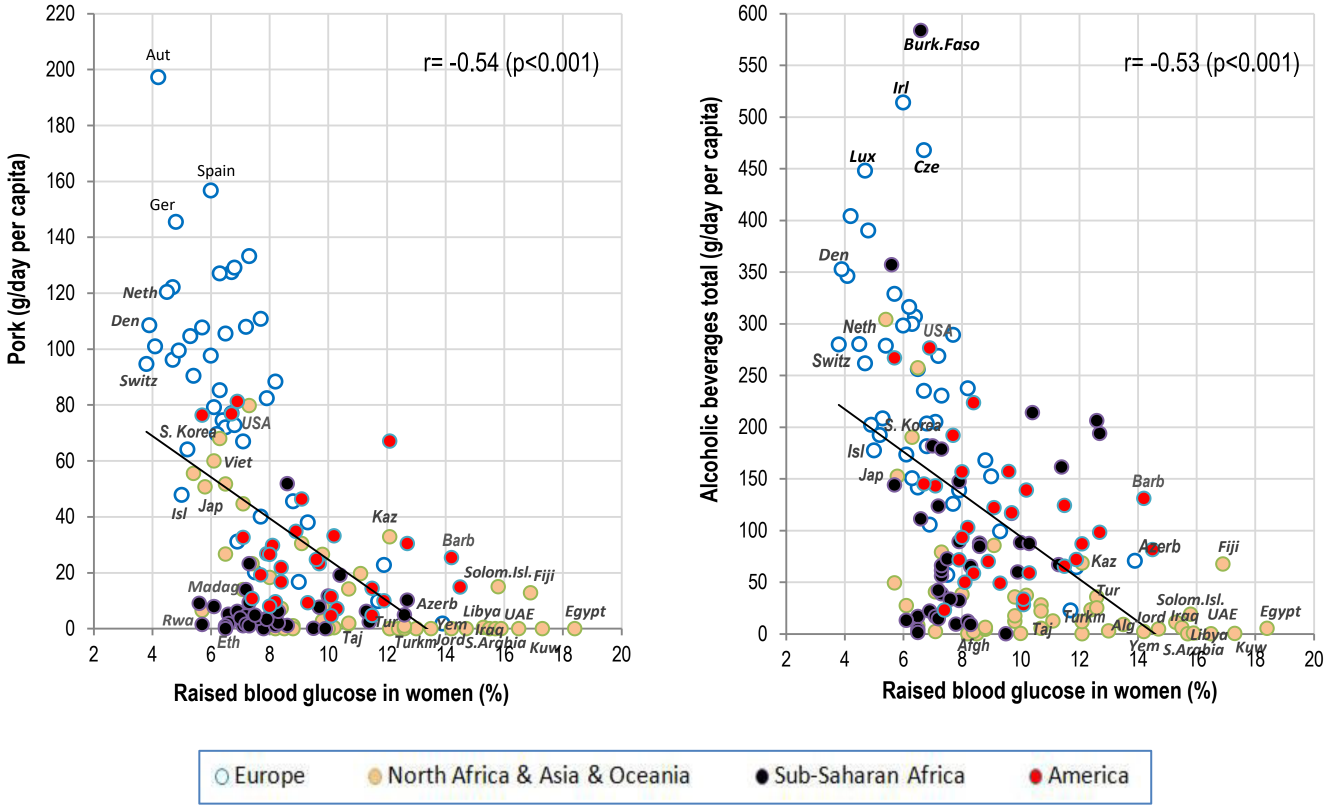

Figure S24. Relationship between the prevalence of women's raised blood glucose (%; WHO, 2010) and the mean consumption of pork (g/day per capita; FAOSTAT, 1993-2011).

Figure S25. Relationship between the prevalence of women's raised blood glucose (%; WHO, 2010) and the mean consumption of alcoholic beverages (g/day per capita, FAOSTAT, 1993-2011).

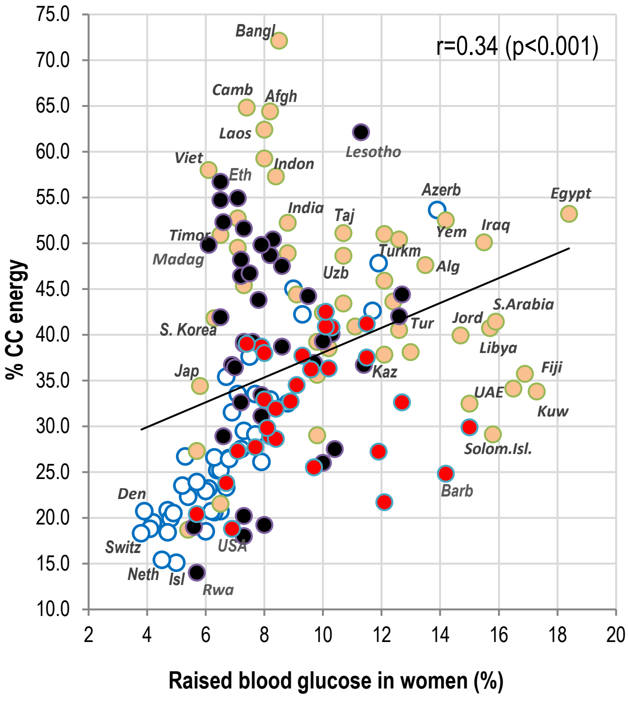

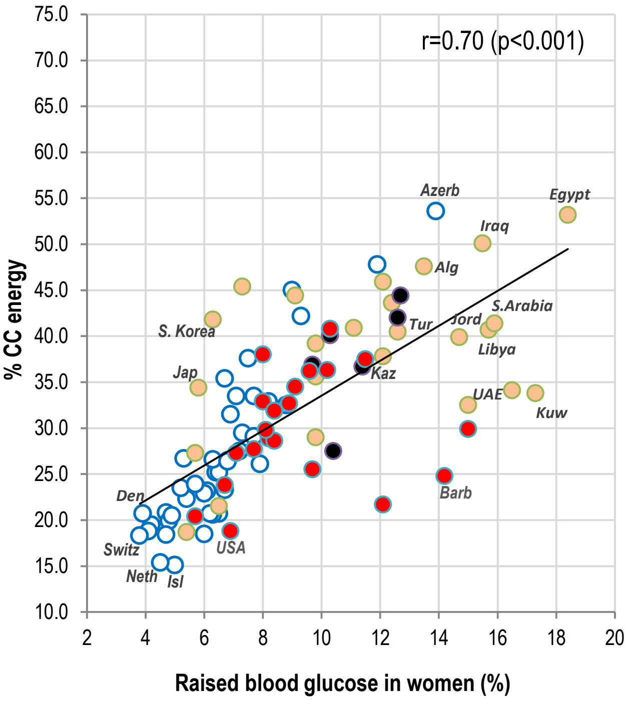

Figure S26. Relationship between the prevalence of women's raised blood glucose (%; WHO, 2010) and the mean proportion of carbohydrate energy from cereals (% CC energy) in the diet (FAOSTAT, 1993-2011).
Total sample (n=158).

Figure S27. Relationship between the prevalence of women's raised blood glucose (%; WHO, 2010) and the mean proportion of carbohydrate energy from cereals (% CC energy) in the diet (FAOSTAT, 1993-2011).
Countries with health expenditure above 500 USD (n=92).

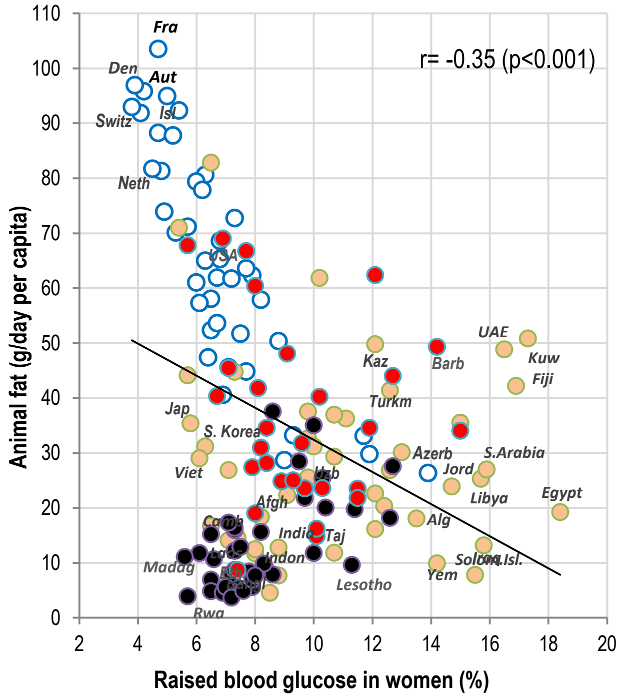

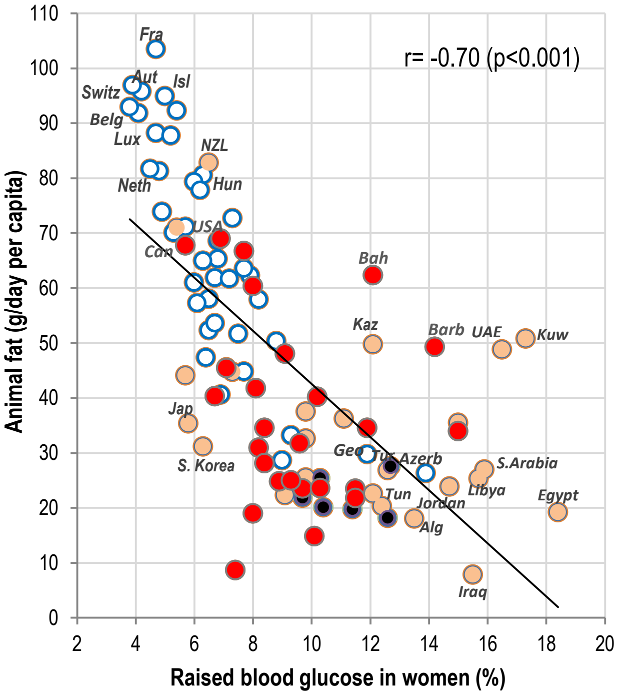

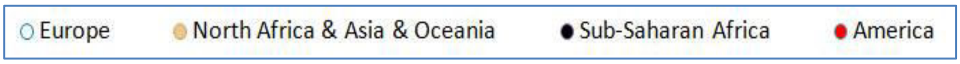

Figure S28. Relationship between the prevalence of women's raised blood glucose (%; WHO, 2010) and the mean consumption of animal fat (g/day per capita; FAOSTAT, 1993-2011).
Total sample (n=158).

Figure S29. Relationship between the prevalence of women's raised blood glucose (%; WHO, 2010) and the mean consumption of animal fat (g/day per capita; FAOSTAT, 1993-2011).
Countries with health expenditure above 500 USD (n=92).

Smoking

Table S9. Correlation between smoking and health indicators**.** All countries (n=115).

	Current smoking of any tobacco product		Daily smoking of any tobacco product		Current smoking of cigarettes		Daily smoking of cigarettes	
	Men	Women	Men	Women	Men	Women	Men	Women
Raised blood pressure (Men)	0.17		0.18		0.11		0.13	
Raised blood pressure (Women)		-0.42		-0.43		-0.49		-0.48
CVD mortality (Men)	0.53		0.52		0.52		0.50	
CVD mortality (Women)		-0.37		-0.38		-0.40		-0.40
Raised cholesterol (Men)	-0.03		0.01		0.09		0.13	
Raised cholesterol (Women)		0.62		0.63		0.67		0.66
Raised blood glucose (Men)	0.19		0.19		0.17		0.13	
Raised blood glucose (Women)		-0.44		-0.46		-0.43		-0.47
Health expenditure	-0.26	0.55	-0.23	0.57	-0.16	0.59	-0.13	0.60
Life expectancy	-0.07	0.57	-0.04	0.58	0.04	0.63	0.06	0.61

Table S10. Correlation between smoking and health indicators. Non-European countries (n=76).

	Current smoking of any tobacco product		Daily smoking of any tobacco product		Current smoking of cigarettes		Daily smoking of cigarettes	
	Men	Women	Men	Women	Men	Women	Men	Women
Raised blood pressure (Men)	-0.05		-0.07		-0.17		-0.19	
Raised blood pressure (Women)		-0.44		-0.46		-0.56		-0.57
CVD mortality (Men)	0.36		0.34		0.32		0.29	
CVD mortality (Women)		-0.35		-0.36		-0.42		-0.42
Raised cholesterol (Men)	0.06		0.06		0.20		0.18	
Raised cholesterol (Women)		0.41		0.41		0.51		0.49
Raised blood glucose (Men)	0.15		0.17		0.15		0.12	
Raised blood glucose (Women)		-0.13		-0.15		-0.11		-0.18
Health expenditure	-0.17	0.44	-0.14	0.48	-0.05	0.52	-0.03	0.58
Life expectancy	0.01	0.43	0.02	0.44	0.13	0.55	0.12	0.52

Level of significance:

**Positive correlates**			**Negative correlates**		
**p<0.05**	**p<0.01**	**p<0.001**	**p<0.05**	**p<0.01**	**p<0.001**

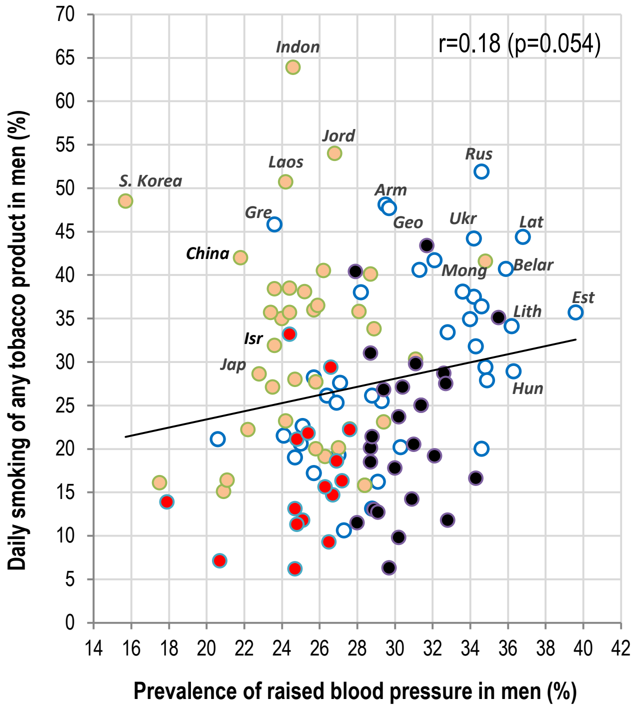

Figure S30. Relationship between the prevalence of men's raised blood pressure (WHO, 2008) and the prevalence of daily smoking of any tobacco product in men.

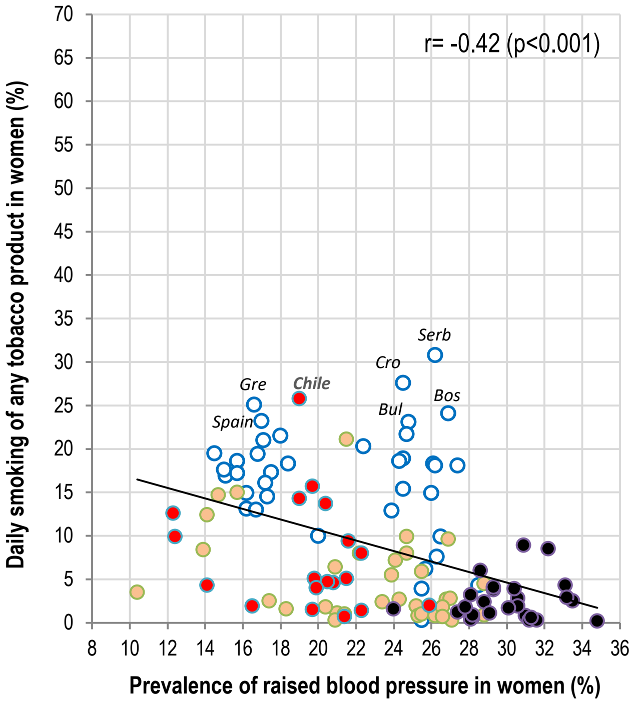

Figure S31. Relationship between the prevalence of women's raised blood pressure (WHO, 2008) and the prevalence of daily smoking of any tobacco product in women.

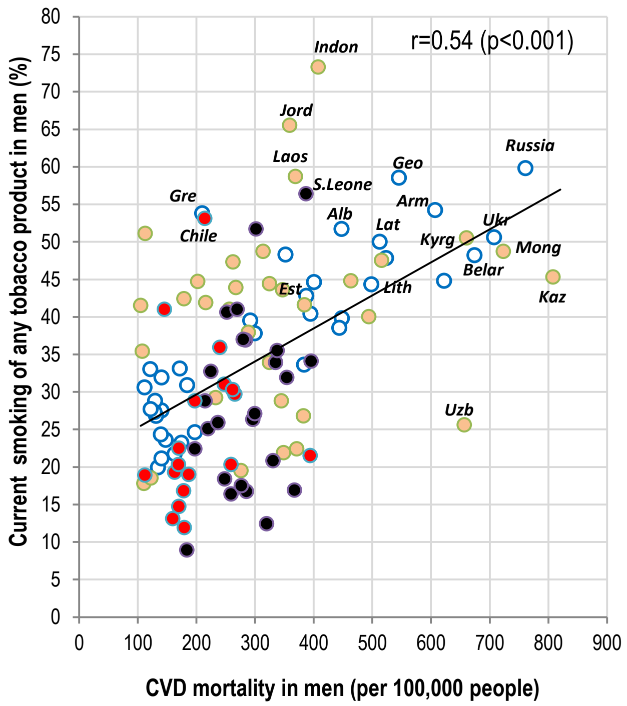

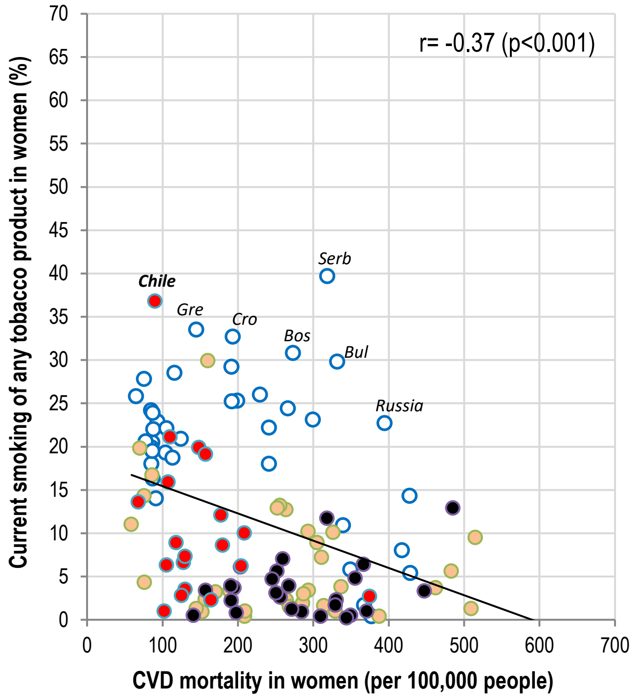

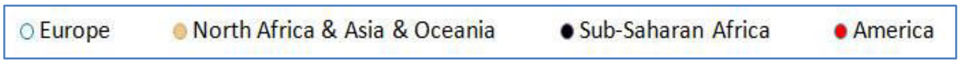

Figure S32. Relationship between the prevalence of men's CVD mortality (WHO, 2012) and the prevalence of current smoking of any tobacco product in men.

Figure S33. Relationship between the prevalence of women's CVD mortality (WHO, 2012) and the prevalence of current smoking of any tobacco product in women.

Physical activity

Table S11. Correlation between physical activity and health indicators in 123 countries.

	% Insufficiently active adults					
	Total sample (n=123)		Europe (n=35)		Non-European countries (n=88)	
	Men	Women	Men	Women	Men	Women
Raised blood pressure (Men)	-0.17		-0.50		-0.08	
Raised blood pressure (Women)		-0.26		-0.55		-0.27
CVD mortality (Men)	-0.18		-0.55		-0.07	
CVD mortality (Women)		-0.25		-0.53		-0.23
Raised cholesterol (Men)	0.31		0.43		0.46	
Raised cholesterol (Women)		0.31		0.43		0.48
Raised blood glucose (Men)	0.45		-0.09		0.52	
Raised blood glucose (Women)		0.35		-0.40		0.46

Level of significance:

Positive correlates			Negative correlates		
p<0.05	p<0.01	p<0.001	p<0.05	p<0.01	p<0.001

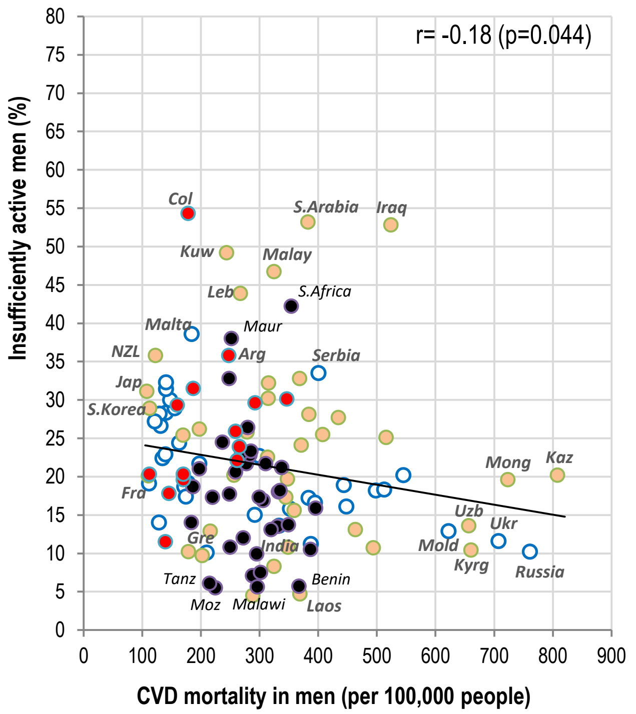

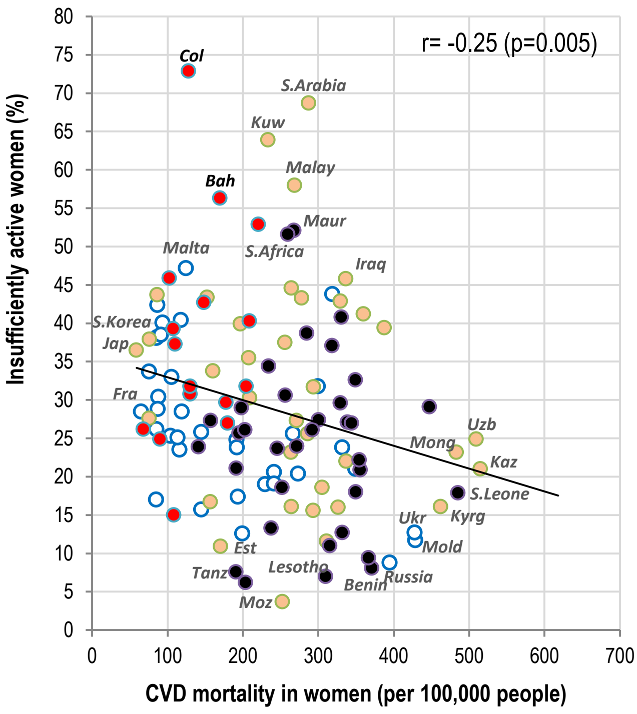

Europe · North Africa & Asia & Oceania · Sub-Saharan Africa · America

Figure S34. Relationship between the self-reported prevalence of physical activity (WHO, 2010) and men's CVD mortality (WHO, 2012).

Figure S35. Correlation between the self-reported prevalence of physical activity and women's CVD mortality (WHO, 2012).

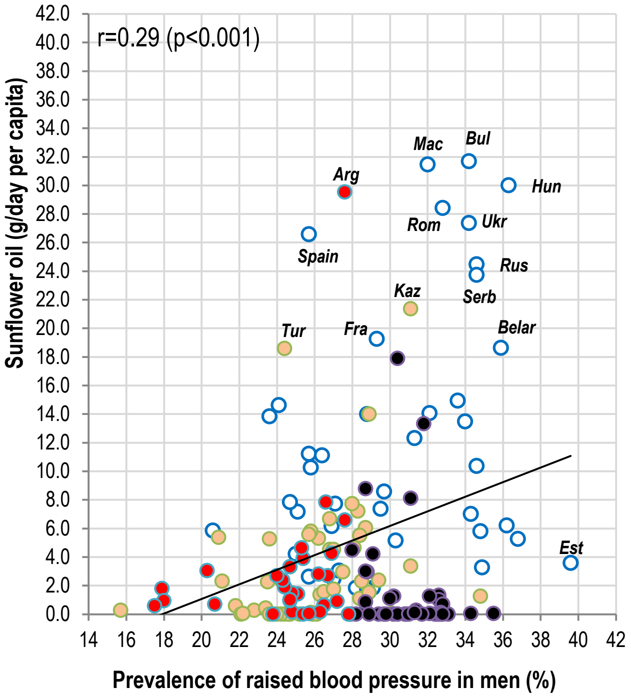

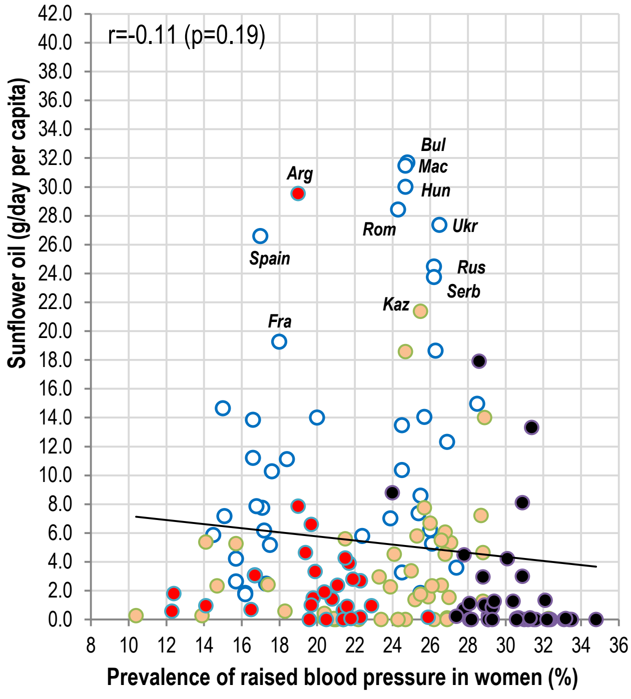

Figure S36. Relationship between the prevalence of men's raised blood pressure (%; WHO, 2010) and the mean consumption of sunflower oil (FAOSTAT, 1993-2011).

Figure S37. Relationship between the prevalence of women's raised blood pressure (%; WHO, 2010) and the mean consumption of sunflower oil (FAOSTAT, 1993-2011).

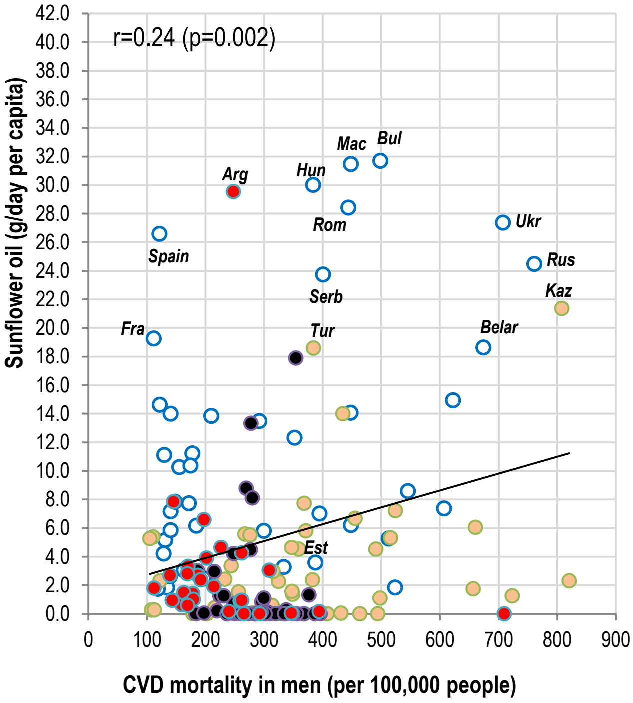

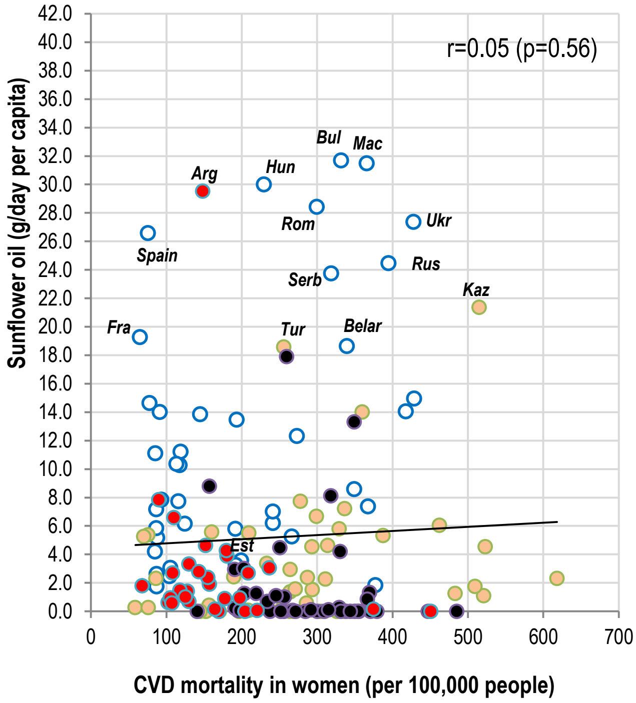

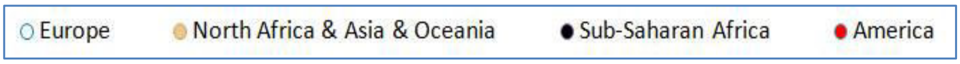

Figure S38. Relationship between men's CVD mortality (WHO, 2012) and the mean consumption of sunflower oil (FAOSTAT, 1993-2011).

Figure S39. Relationship between women's CVD mortality (WHO, 2012) and the mean consumption of sunflower oil (FAOSTAT, 1993-2011).

Factor analyses

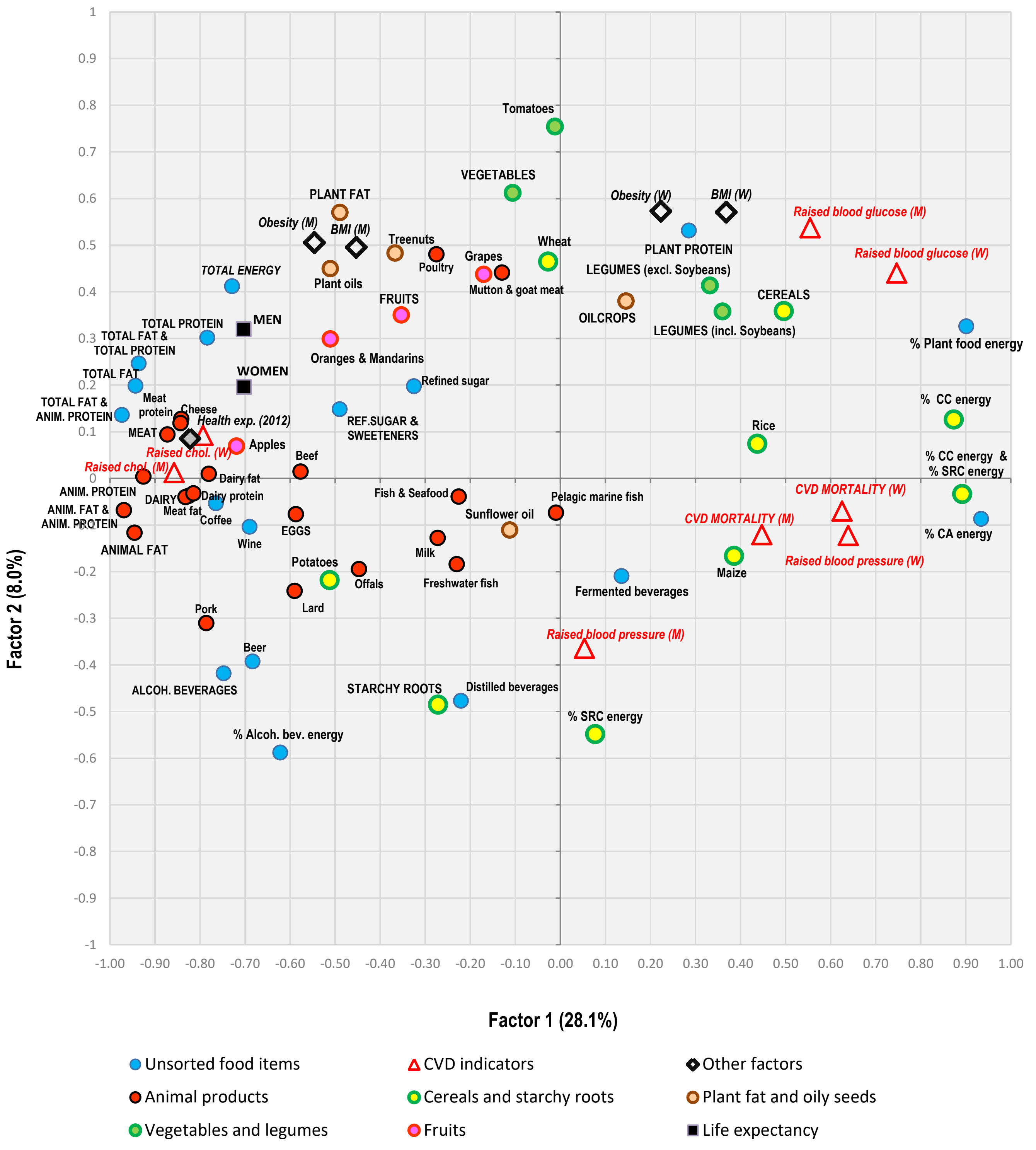

Figure S40. Factor analysis including 75 variables in 92 countries with health expenditure above 500 USD per capita explaining 36.1% variability (Factor 1 vs. Factor 2).

Abbreviations: % CC energy = the mean proportion of carbohydrate energy from cereals; % SRC energy = the mean proportion of carbohydrate energy from starchy roots; % CA energy = the mean proportion of energy from carbohydrates and alcohol.

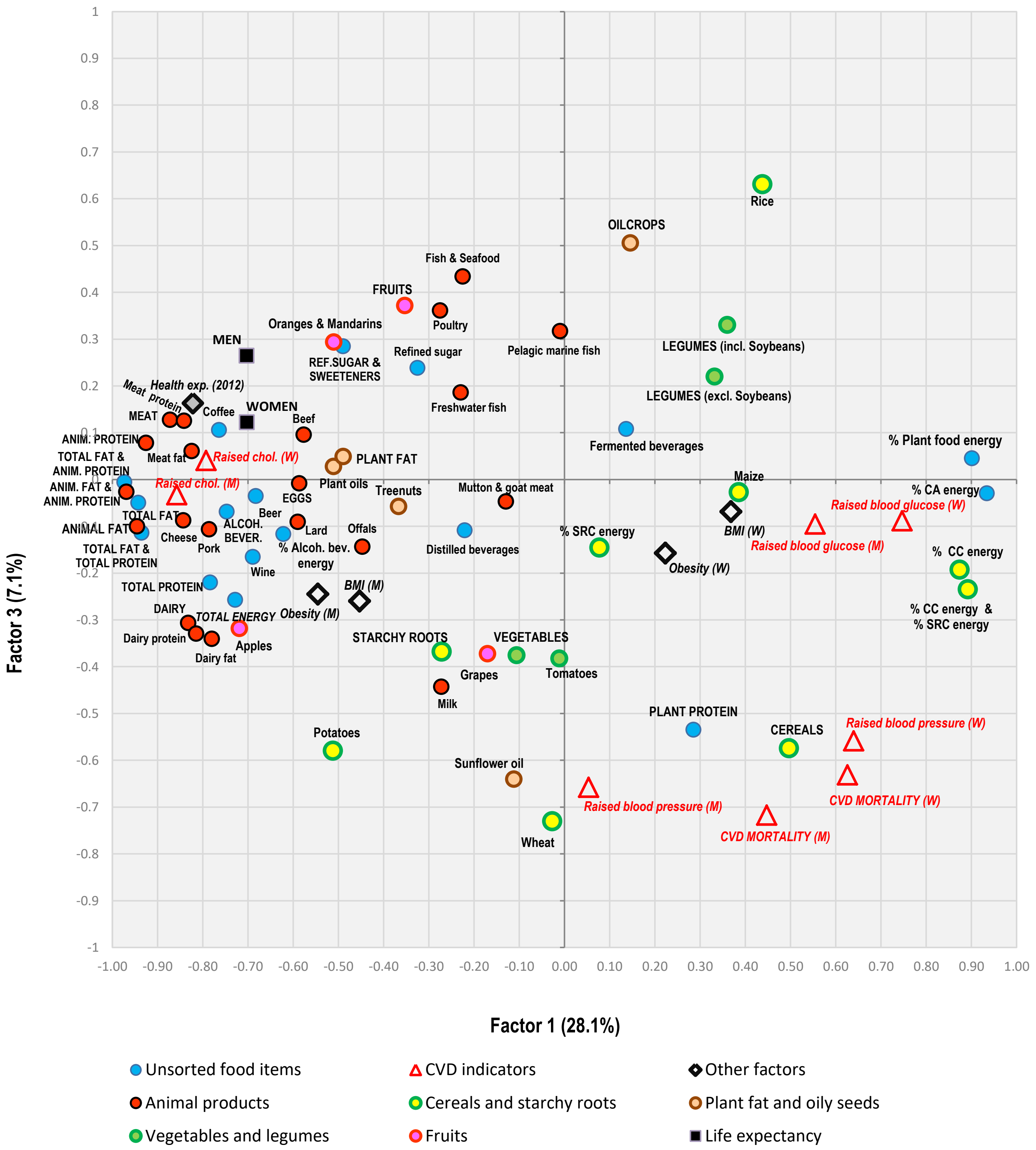

Figure S41. Factor analysis including 75 variables in 92 countries with health expenditure above 500 USD per capita explaining 35.2% variability (Factor 1 vs. Factor 3).

Abbreviations: % CC energy = the mean proportion of carbohydrate energy from cereals; % SRC energy = the mean proportion of carbohydrate energy from starchy roots; % CA energy = the mean proportion of energy from carbohydrates and alcohol.

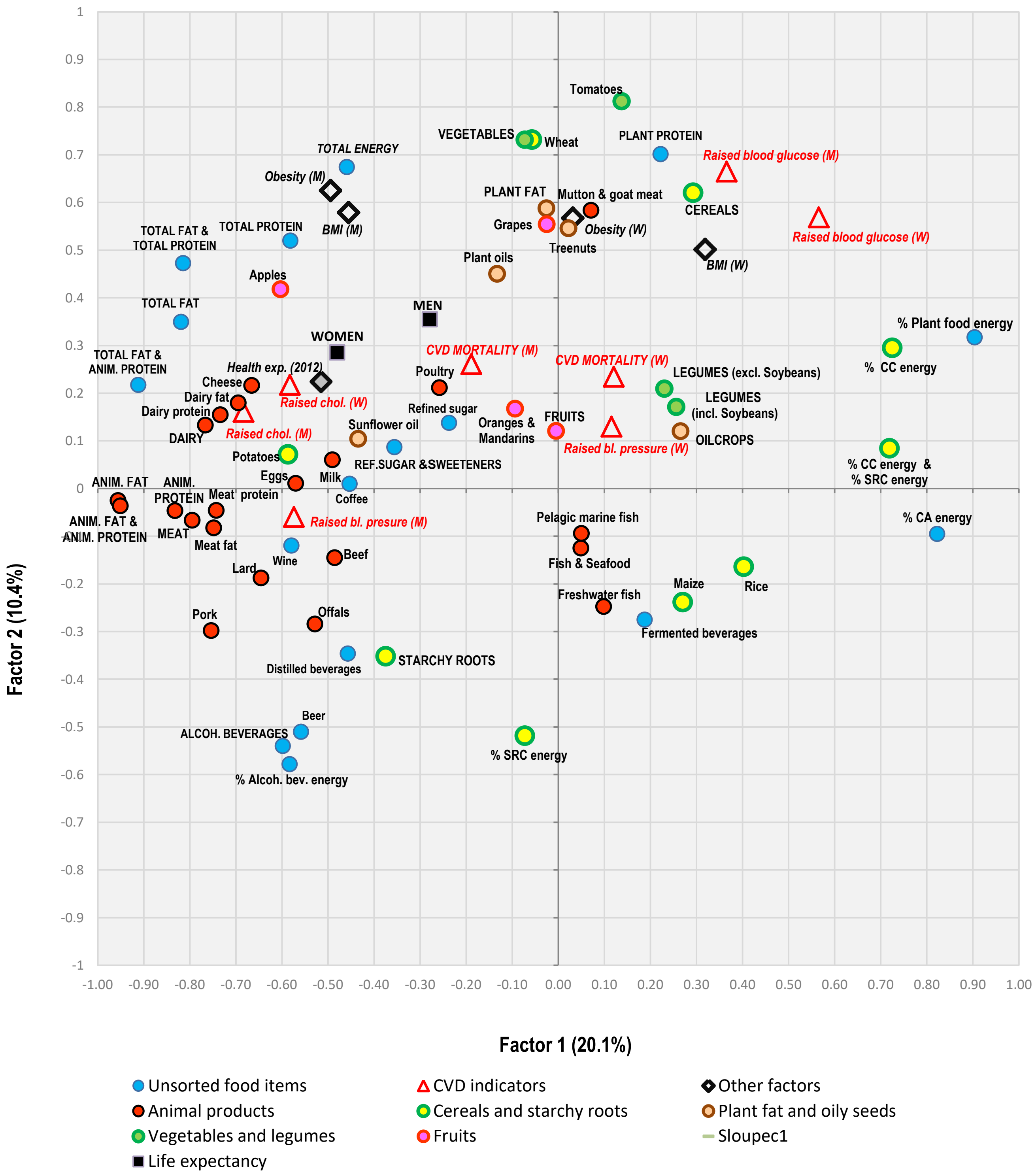

Figure S42. Factor analysis including 75 variables in 61 countries with health expenditure 500-2000 USD per capita explaining 30.5% variability.

Abbreviations: % CC energy = the mean proportion of carbohydrate energy from cereals; % SRC energy = the mean proportion of carbohydrate energy from starchy roots; % CA energy = the mean proportion of energy from carbohydrates and alcohol.

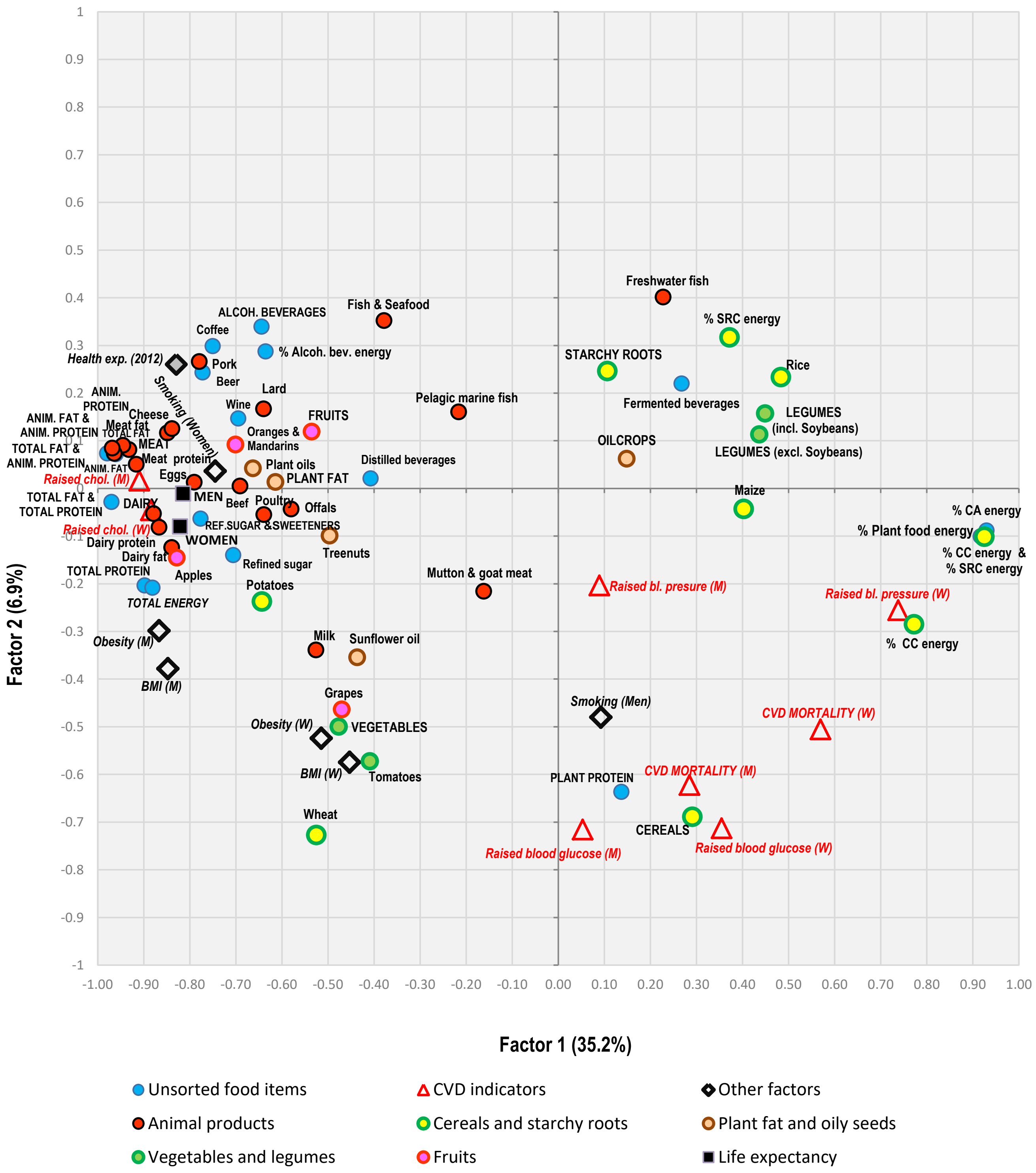

Figure S43. Factor analysis including 77 variables (plus smoking) in 115 countries explaining 42.1% variability. Smoking in this plot represents 'Current smoking of any tobacco product'.

Abbreviations: % CC energy = the mean proportion of carbohydrate energy from cereals; % SRC energy = the mean proportion of carbohydrate energy from starchy roots; % CA energy = the mean proportion of energy from carbohydrates and alcohol.

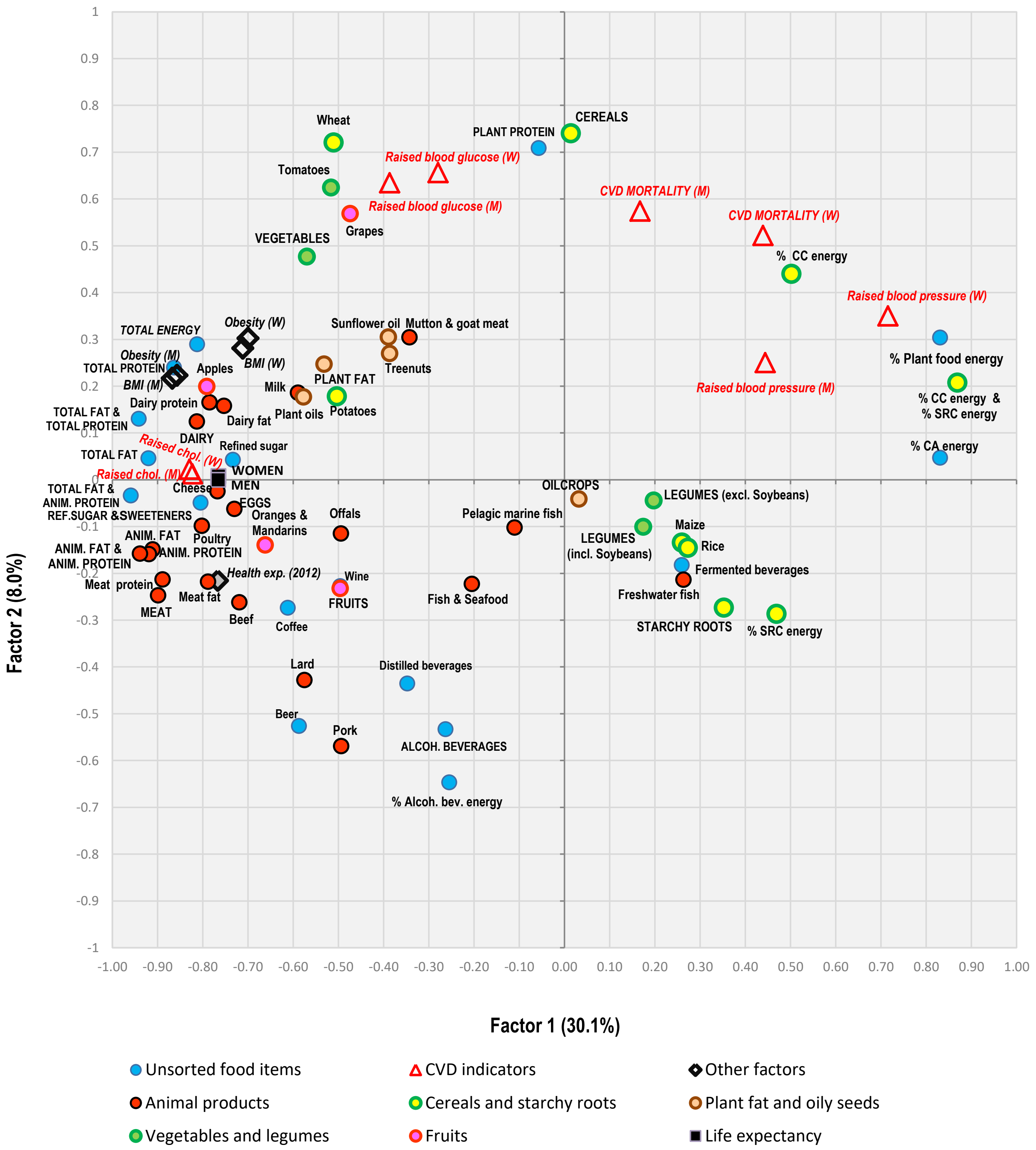

Figure S44. Factor analysis including 75 variables in 116 non-European countries explaining 38.1% variability.

Abbreviations: % CC energy = the mean proportion of carbohydrate energy from cereals; % SRC energy = the mean proportion of carbohydrate energy from starchy roots; % CA energy = the mean proportion of energy from carbohydrates and alcohol.

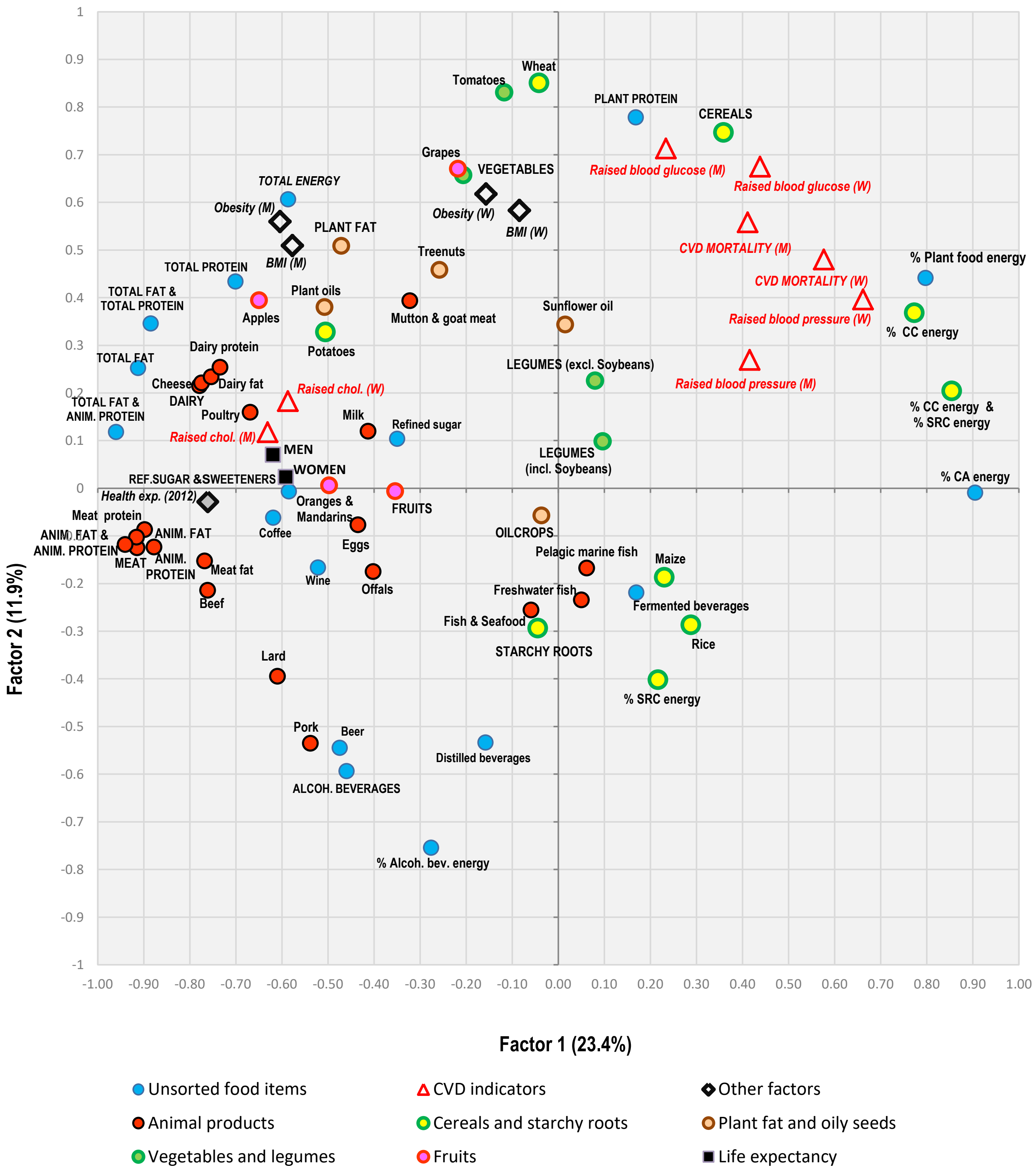

Figure S45. Factor analysis including 75 variables in 51 non-European countries with health expenditure above 500 USD per capita explaining 35.3% variability.

Abbreviations: % CC energy = the mean proportion of carbohydrate energy from cereals; % SRC energy = the mean proportion of carbohydrate energy from starchy roots; % CA energy = the mean proportion of energy from carbohydrates and alcohol.

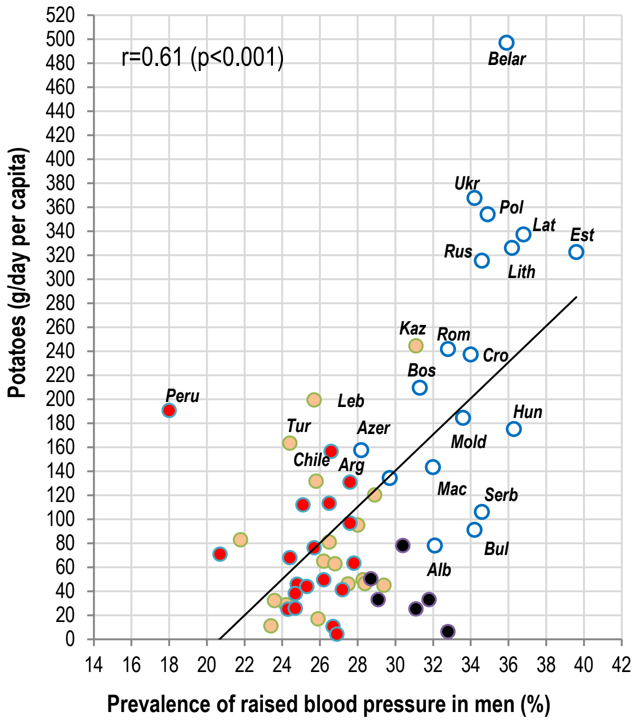

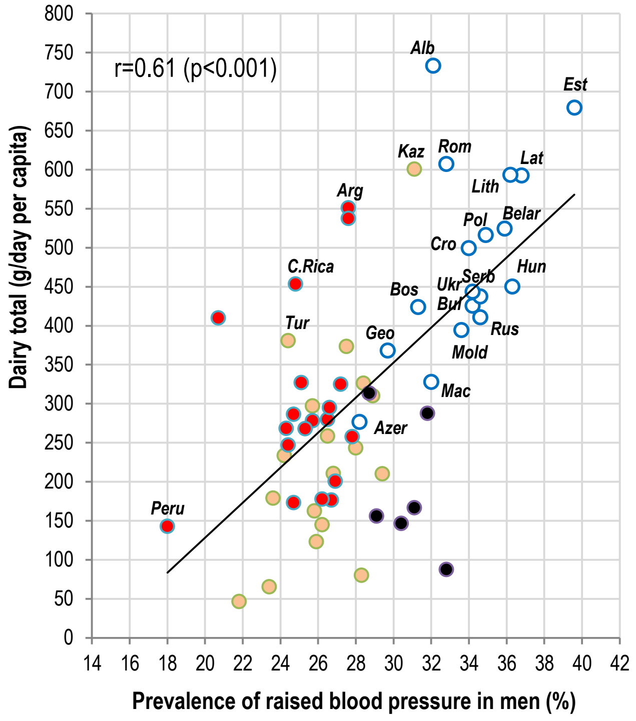

Figure S46. Relationship between the prevalence of men's raised blood pressure (%; WHO, 2010) and the mean consumption of potatoes (FAOSTAT, 1993-2011).
Countries with health expenditure between 500-2000 USD per capita (n=61).

Figure S47. Relationship between the prevalence of women's raised blood pressure (%; WHO, 2010) and the mean consumption of dairy (FAOSTAT, 1993-2011).
Countries with health expenditure between 500-2000 USD per capita (n=61).

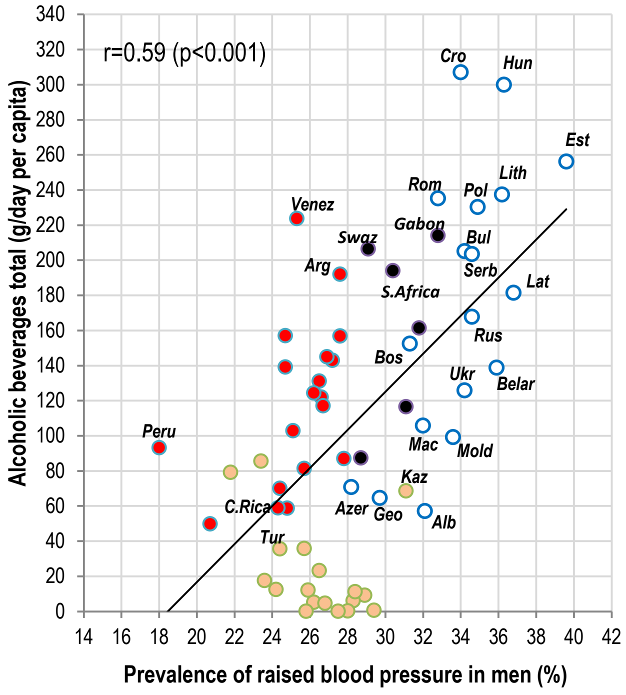

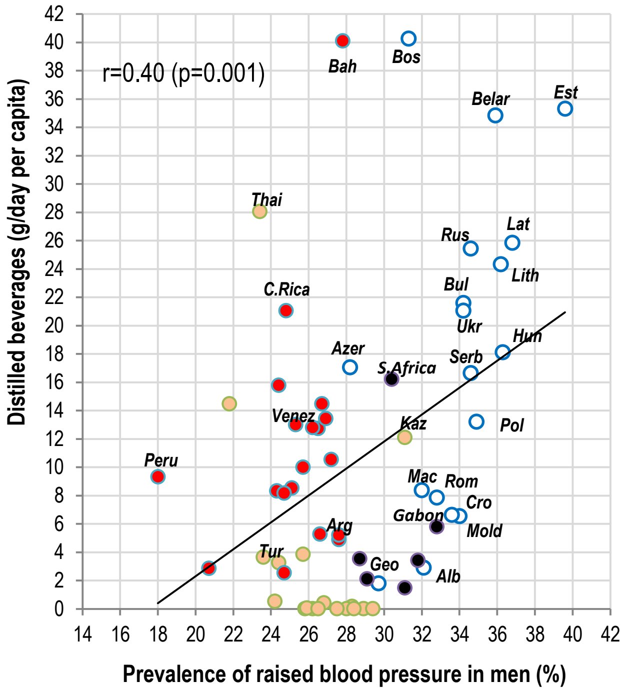

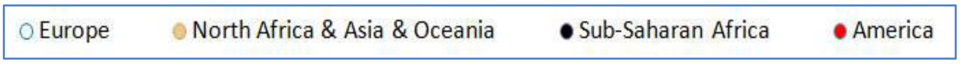

Figure S48. Relationship between men's CVD mortality (WHO, 2012) and the mean consumption of alcoholic beverages (FAOSTAT, 1993-2011).
Countries with health expenditure between 500-2000 USD per capita (n=61).

Figure S49. Relationship between women's CVD mortality (WHO, 2012) and the mean consumption of distilled beverages (FAOSTAT, 1993-2011).
Countries with health expenditure between 500-2000 USD per capita (n=61).

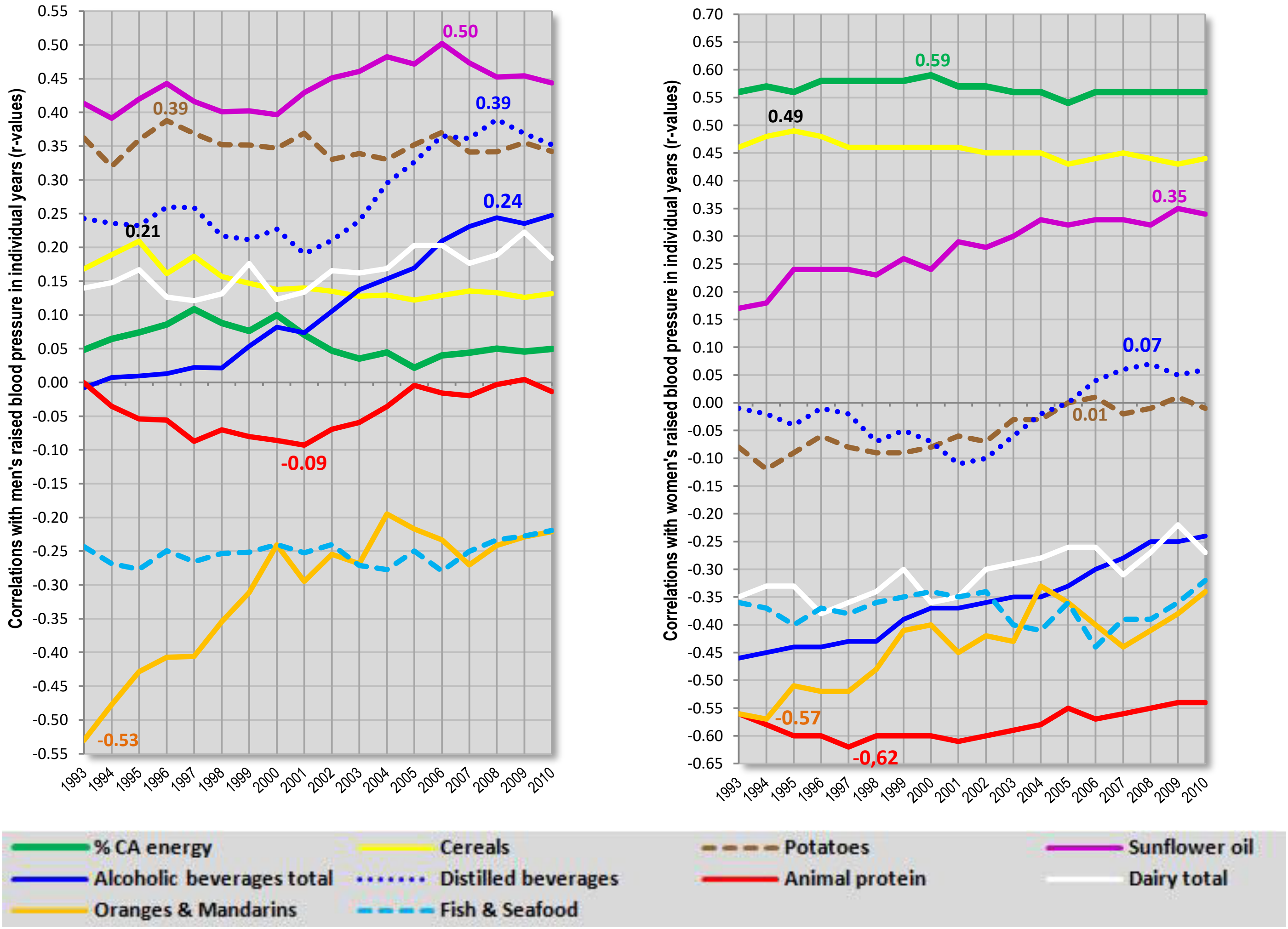

Figure S50. Temporal changes in correlation coefficients (r-values) between men's raised blood pressure (2010) and 10 food items. **Countries with health expenditure above 500 USD per capita (n=91).**

Figure S51. Temporal changes in correlation coefficients (r-values) between women's raised blood pressure (2010) and 10 food items. **Countries with health expenditure above 500 USD per capita (n=91).**

Abbreviations: % CA energy = the mean proportion of energy from carbohydrates and alcohol.
